# Polypeptide-engineered lipid nanoparticles for mRNA delivery with limited immunogenicity

**DOI:** 10.1038/s41467-026-73698-6

**Published:** 2026-05-29

**Authors:** Jin-Yue Zeng, Yue Zhang, Gui Zhao, Brandon Yi Loong Seow, Nithiyaa Bala Krishnan, Jin Hao Goh, Yi Yan Yang

**Affiliations:** Bioprocessing Technology Institute (BTI), Agency for Science, Technology and Research (A*STAR), 20 Biopolis Way, The Centros #06-01, 138668 Singapore, Singapore

**Keywords:** Transfection, Drug delivery, Nanobiotechnology

## Abstract

Lipid nanoparticles (LNPs) have shown great potential for mRNA delivery, with polyethylene glycol (PEG) lipids playing a critical role in modulating particle size, stability and biodistribution. However, most PEGylated LNPs induce anti-PEG antibodies, leading to hypersensitivity and diminished efficacy upon repeated administration. Here we report hydrophilic, nonionic and biodegradable poly(D, L-serine) (pDLS) lipids as PEG-lipid alternatives in LNP formulations. Through systematic structural screening, we identify optimal lipid architectures that yield colloidally stable pDLS-LNPs with high mRNA encapsulation and transfection efficiency. Compared to the clinically approved BNT162b2 formulation (ALC-LNP), pDLS-LNPs loaded with SARS-CoV-2 spike mRNA achieve superior mRNA delivery, and elicit robust cellular and humoral immune responses in mice, without inducing systemic toxicity. Notably, repeated dosing with pDLS-LNPs triggers minimal anti-pDLS IgM production, unlike PEG-based counterparts. Furthermore, pDLS-LNPs remain stable under frozen storage for over 6 months. These findings establish polypeptide-based pDLS-LNPs as promising, immunologically inert alternatives to PEGylated LNPs for safe and effective mRNA delivery.

## Abstract (web summary)

PEG is an important component in lipid nanoparticles but can cause anti-PEG antibody responses. Here, the authors develop biodegradable poly-DL-serine lipids as PEG replacements and demonstrate mRNA delivery with limited immunogenicity, demonstrating improved safety.

## Introduction

Messenger RNA (mRNA)-based therapeutics provide promising avenues for the prevention and treatment of a wide array of diseases, such as infectious diseases^1–3^, cancers^4–6^, autoimmune diseases^7,8^, and cardiovascular disorders^9,10^. Effective delivery of mRNA is essential to realize its therapeutic potential, as naked mRNA is highly susceptible to degradation and requires a carrier to facilitate cellular uptake^11–13^. Lipid nanoparticles (LNPs) have emerged as the leading delivery vehicles of mRNA, exemplified by their important role in BioNTech/Pfizer’s BNT162b2 and Moderna’s mRNA-1273 COVID-19 vaccines^14,15^. In general, LNPs consist of an ionizable lipid to bind mRNA and enable endosomal escape of mRNA into cytoplasm, cholesterol to stabilize the LNP structure, a helper lipid to promote cell binding and membrane fusion, and a polyethylene glycol (PEG) lipid to maintain colloidal stability and reduce opsonization^16,17^. Notably, both BNT162b2 and mRNA-1273 incorporate PEG lipid to stabilize their LNPs^18^. However, recent studies revealed that PEGylated mRNA-LNPs can induce or amplify anti-PEG antibody production in humans^19–21^. Such anti-PEG antibodies can cause accelerated blood clearance of PEGylated mRNA-LNPs and adverse effects^22^. Moreover, pre-existing anti-PEG antibodies have been detected in 98-99% of unvaccinated individuals, with approximately 3–4% of the population exhibiting markedly elevated levels^23^. These antibodies likely arise from widespread exposure to PEG-containing products, including foods, cosmetics, and pharmaceuticals^24,25^. While the clinical implications of pre-existing anti-PEG antibodies are still under investigation, studies have shown that individuals with high anti-PEG antibody levels could be at increased risk for hypersensitivity reactions, including rare allergic responses to PEGylated mRNA-LNP vaccination^23,26^. The immunogenicity of PEG has therefore promoted growing interest in the development of PEG alternatives for mRNA therapeutics, particularly for applications requiring repeated dosing^27,28^.

Considerable research efforts are underway to explore viable alternatives. Various hydrophilic, biocompatible polymers have emerged as potential PEG substitutes, including polysaccharides^29,30^, polyglycerols^31^, poly(zwitterions)^32,33^, poly(vinylpyrrolidone)^34^, poly(2-oxazoline)s^35,36^, and polypeptoids^37–39^. Among these emerging alternatives, synthetic polypeptides, especially polysarcosine, have shown particular promise for mRNA delivery due to their PEG-like physicochemical characteristics and intrinsic biocompatibility^40–42^. However, most conventional polypeptides derived from essential amino acids frequently possess inherent charges or excessive hydrophobicity, which may destabilize mRNA-loaded LNPs when used as PEG replacements^43^. Despite these significant advances in PEG-alternative development, substantial optimization is still required to achieve scalable production, therapeutic efficacy, safety, and long-term stability comparable to conventional PEGylated LNP for mRNA delivery^44–48^. Therefore, the development of PEG-free LNPs that simultaneously satisfy the requirements of biosafety, delivery efficacy, and long-term storage stability for nucleic acid therapeutics remains a significant unmet challenge.

**Fig. 1:**
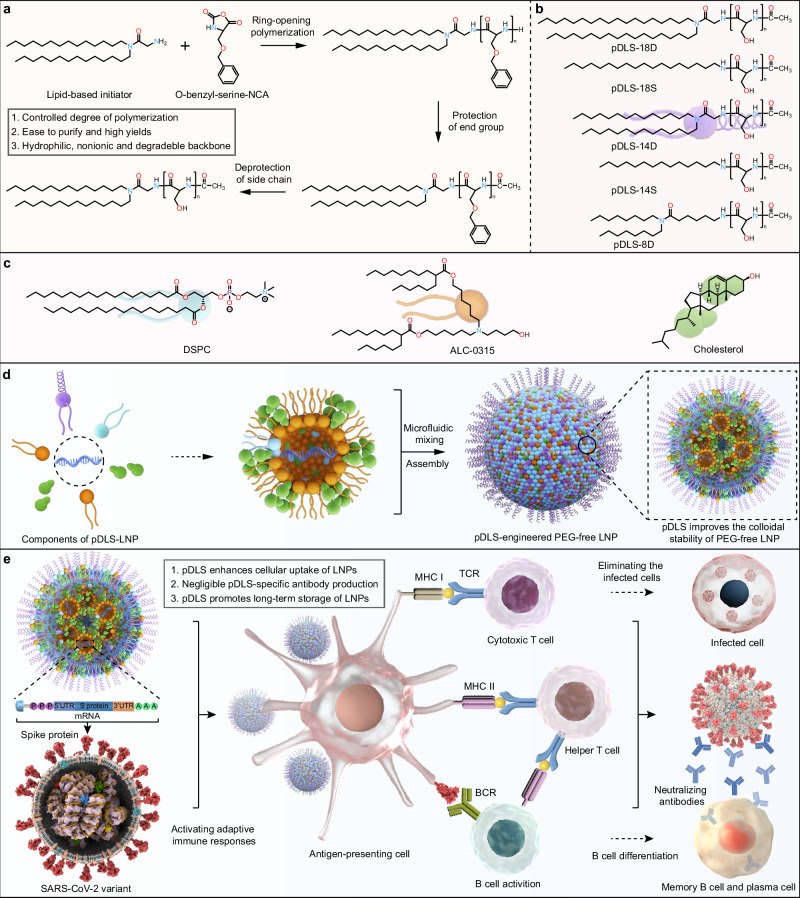
Design, synthesis, and application of amphiphilic pDLS lipids to formulate PEG-free LNPs for effective mRNA delivery. **a** Synthesis of amphiphilic pDLS lipids. **b** Chemical structures of the synthesized pDLS lipids. **c** Structures of formulation components used in PEG-free pDLS-LNPs: helper lipid DSPC, ionizable lipid ALC-0315, and cholesterol. **d** Formulation strategy of mRNA-loaded pDLS-LNP. **e** Schematic illustration of SARS-CoV-2 spike mRNA-loaded pDLS-LNP inducing cellular and humoral immune responses.

Herein, we report a series of hydrophilic, nonionic, and biodegradable poly(D, L-serine) (pDLS) lipids synthesized via the controlled ring-opening polymerization (ROP) of serine N-carboxyanhydride (NCA) to formulate PEG-free LNPs (Fig. 1a, b). This synthetic approach exhibits unique advantages, including ease of purification, high yields, and control over the degree of polymerization. Although sarcosine itself is a naturally occurring metabolite in mammalian systems. However, polysarcosine is a synthetic peptoid polymer whose N-substituted backbone is not produced biologically and is generally resistant to proteolytic degradation. In contrast, polyserine is a true polypeptide composed of a canonical amino acid and is therefore more likely to undergo enzymatic processing into metabolizable products. The co-assembly of pDLS lipids with ionizable lipid, helper lipid, cholesterol, and mRNA yields colloidally stable PEG-free pDLS-LNPs (Fig. 1c, d), which exhibit good size distribution, near-neutral zeta potential, and comparable mRNA encapsulation efficiency. Systematic formulation screening of structurally diverse pDLS lipids allows for the identification of optimal lipid tail structures and pDLS chain length. The resulting pDLS-LNPs show improved cellular uptake and endosomal escape, resulting in a significant increment in mRNA transfection efficiency compared with the approved ALC-LNP used in the BNT162b2 vaccine. In vivo bioluminescence imaging demonstrates effective delivery of luciferase-encoding mRNA to lymph nodes, a key site for inducing and regulating adaptive immune responses. Moreover, SARS-CoV-2 spike mRNA-loaded pDLS-LNPs elicit strong cellular and humoral immune responses without inducing systemic toxicity in mice (Fig. 1e). Importantly, repeated dosing of the leading pDLS-LNPs does not trigger the production of anti-pDLS IgM responses, in contrast to the strong anti-PEG IgM responses observed with ALC-LNPs. Finally, the leading pDLS-LNP formulation maintains its physicochemical properties and in vivo transfection efficiency after storage at -80 °C for at least 6 months, demonstrating excellent storage stability. Taken together, these findings highlight the potential of pDLS-LNPs as a promising, immunologically inert alternative to PEGylated LNPs for safe and effective mRNA delivery.

## Results and discussion

### Design and synthesis of amphiphilic pDLS lipids

Due to their chiral and peptidic backbones, both poly(L-serine) and poly(D-serine) tend to adopt ordered β-sheet secondary structures, which contribute to strong hydrophobicity^49^. To mitigate β-sheet formation and enhance hydrophilicity, amphiphilic pDLS lipids were synthesized via the random copolymerization of serine NCA monomers of opposite chirality, followed by deprotection. Specifically, o-benzyl-D-serine NCA (D-serine NCA) and o-benzyl-L-serine NCA (L-serine NCA) were first synthesized (Supplementary Fig. 1). The successful synthesis of NCA monomers was verified by ^1^H and ^13^C NMR spectra (Supplementary Figs. 2–5). The amphiphilic pDLS lipids were then made by ROP of D-serine NCA and L-serine NCA in a random form using primary amine-containing lipids of different lengths (C8-C18) and tail structures (a single or double alkyl chain) as an initiator (Supplementary Fig. 6 and Table 1). Acetic anhydride was used to cap the end group (primary amine), followed by deprotection to release the hydroxyl group on the side chain of polyserine. To explore the impact of pDLS length on mRNA delivery, we synthesized a series of pDLS lipids (pDLS1-pDLS5) using a double C14-chain initiator, achieving degrees of polymerization (DP) ranging from 18 to 45. Furthermore, to study the effect of lipid tail structure, pDLS variants were synthesized using primary amine-containing initiators with C8, C14, and C18 chains in either single (S) or double (D) chain configurations: pDLS8D, pDLS14D (same as pDLS3), pDLS18D, pDLS14S, and pDLS18S. The structures of intermediates and final pDLS lipids were confirmed by ^1^H NMR spectra (Supplementary Figs. 7–22). DPs of serine in pDLS lipids were determined by the integration areas from the peak **g** of the polyserine and the peak **a** of the alkyl lipid (Supplementary Figs. 13–22). The characteristics of amphiphilic pDLS lipids are listed in Supplementary Table 2. Poly(D/L-serine) is water-soluble, nonionic, biodegradable, as well as non-cytotoxic, and has a large number of hydroxyl groups for further functionalization, making it suitable for biomedical applications^50^.

### Optimization and characterization of pDLS-LNP formulations for mRNA delivery

**Fig. 2:**
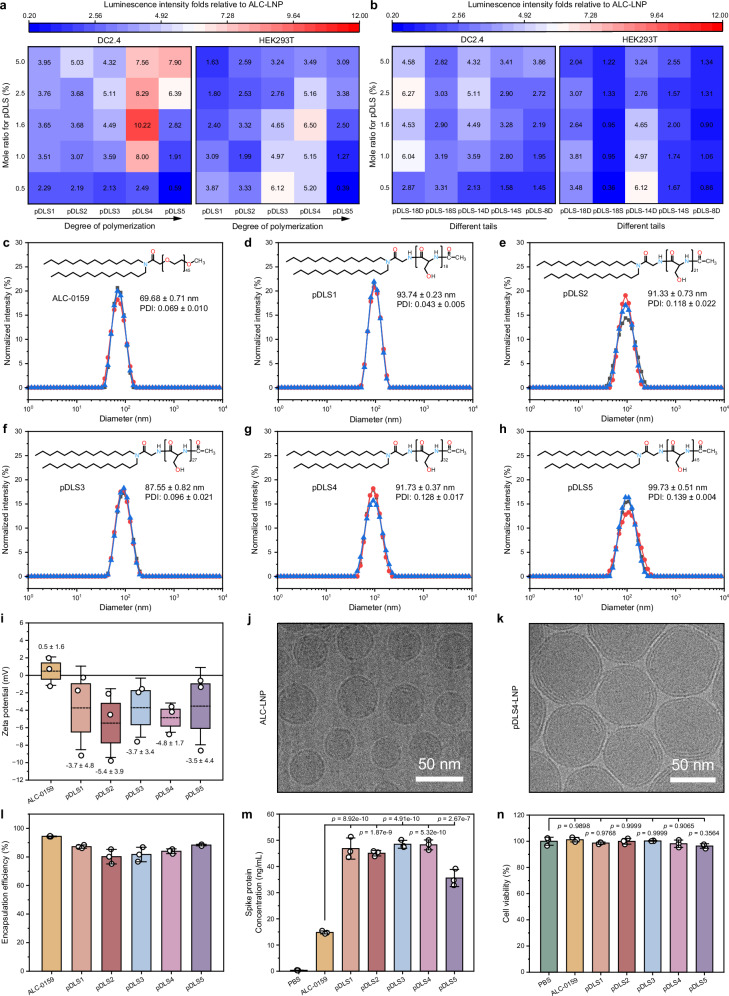
Functional screening and characterization of pDLS-LNPs for mRNA delivery. **a** Screening of mRNA transfection efficiency in the mouse dendritic cell line (DC2.4) and the human embryonic kidney cell line (HEK293T) using pDLS-LNPs formulated with pDLS lipids of varying DPs, compared to PEGylated ALC-LNP (Fluc mRNA-loaded LNPs were prepared via pipette mixing). **b** Screening mRNA transfection efficiency in DC2.4 and HEK293T cells using pDLS-LNPs formulated with pDLS lipids with different hydrophobic tail structures (pDLS14D corresponds to pDLS3), relative to ALC-LNP (Fluc mRNA-loaded LNPs were prepared via pipette mixing). Size distribution profiles of SARS-CoV-2 spike mRNA-loaded LNPs prepared via microfluidic mixing: **c** ALC-LNP; **d** pDLS1-LNP; **e** pDLS2-LNP; **f** pDLS3-LNP; **g** pDLS4-LNP; **h** pDLS5-LNP (*n* = 3 independent samples; mean ± standard deviation (SD)). **i** Zeta potential of SARS-CoV-2 spike mRNA-loaded pDLS-LNPs (*n* = 3 independent samples; mean ± SD; the center dashed line indicates the mean; the box bounds represent the 25th and 75th percentiles; the whiskers indicate the SD from the mean). Cryo-TEM images of representative FLuc mRNA-loaded LNPs prepared via microfluidic mixing: **j** ALC-LNP; **k** pDLS4-LNP. **l** Encapsulation efficiency of SARS-CoV-2 spike mRNA-loaded pDLS-LNPs prepared via microfluidic mixing (*n* = 3 independent samples; mean ± SD). **m** Spike protein expression in DC2.4 cells transfected with SARS-CoV-2 spike mRNA-loaded pDLS-LNPs prepared via pipette mixing for 48 h (*n* = 3 independent biological samples; mean ± SD). **n** Viability of DC2.4 cells following 48 h treatment with SARS-CoV-2 spike mRNA-loaded pDLS-LNPs prepared via pipette mixing (*n* = 3 independent biological samples; mean ± SD). Statistical analyses were performed using two-tailed one-way analysis of variance (ANOVA) with Tukey’s correction (**m**, **n**). Source data are provided as a Source Data file.

To develop efficient PEG-free LNP formulations using pDLS lipids, we systematically optimized the polymer chain length, alkyl tail structure, and molar ratio of pDLS lipids in the LNP composition. A series of structurally diverse pDLS lipids were co-formulated with the ionizable lipid ALC-0315, cholesterol, and 1,2-distearoyl-*sn*-glycero-3-phosphorylcholine (DSPC) at different molar ratios, resulting in 45 distinct pDLS-LNP formulations loaded with firefly luciferase (FLuc) mRNA (Fig. 1b–d). Details of the PEG-free pDLS-LNP compositions are summarized in Supplementary Tables 3 and 4. Dynamic light scattering (DLS) measurements revealed that most pDLS-LNPs exhibited nanosizes (<200 nm), low polydispersity indices (PDI < 0.2), and near-neutral zeta potentials (within ±10 mV) (Supplementary Figs. 23–26). Ribogreen assays showed that LNPs formulated with pDLS1-pDLS5 had comparable encapsulation efficiencies, reaching up to 75% (Supplementary Fig. 27). Notably, LNPs incorporating pDLS14D (pDLS3), which features a double C14 alkyl chain, consistently demonstrated higher encapsulation efficiencies ranging from 73% to 84% across all tested molar ratios compared to LNPs formulated with other lipid tail architectures (Supplementary Fig. 28). To assess their mRNA transfection capability, we evaluated the transfection efficiency of FLuc mRNA delivered by pDLS-LNPs in DC2.4 and HEK293T cells. Most FLuc mRNA-loaded LNPs formulated with pDLS lipids had superior transfection efficiency in DC2.4 and HEK293 T cells over ALC-LNP formulation, while showing negligible cytotoxicity (Supplementary Figs. 29–36). Additionally, pDLS lipids with higher DPs (e.g., pDLS4 and pDLS5) and longer double-chain alkyl tails (e.g., pDLS18D and pDLS14D) showed higher transfection efficiency (Fig. 2a, b). This enhancement could be attributed to the amphiphilic balance between the hydrophilic polyserine and the hydrophobic lipid tails in pDLS lipids. Specifically, mRNA-LNP formulated with pDLS4 lipid at a molar ratio of 1.6% showed more than 10-fold higher transfection efficiency in DC2.4 cells and more than 6.5-fold in HEK293T cells compared to ALC-LNP, which was formulated with 1.6% of the PEG-lipid ALC-0159^16^. Based on these findings, mRNA-LNPs prepared with 1.6% pDLS1-5 were selected for subsequent studies.

To explore if batch-to-batch variation of pDLS lipid affects LNP performance, we evaluated the physicochemical properties and mRNA transfection efficiency of LNPs formulated with different synthetic batches of pDLS lipid. Taking pDLS2 lipid as an example, pDLS2 lipid from two different batches exhibited a closely matched DP, as verified by ^1^H NMR, with a batch-to-batch variation of 3 units (Supplementary Fig. 22). In addition, FLuc mRNA-loaded LNPs formulated with different batches of pDLS2 exhibited similar physicochemical properties and comparable mRNA encapsulation efficiencies (Supplementary Fig. 37). Moreover, FLuc mRNA-loaded LNPs formulated with pDLS2 from batch 1 or batch 2 mediated comparable mRNA transfection efficiency with cytocompatibility in both HEK293T and DC2.4 cells (Supplementary Fig. 38). Furthermore, we compared the physicochemical properties and mRNA transfection efficiency of LNPs prepared by pipette mixing and microfluidic mixing. The size, PDI, and zeta potential of LNPs were similar regardless of manual or microfluidic mixing (Supplementary Fig. 39a–c). However, microfluidic mixing yielded consistently higher encapsulation efficiencies for FLuc mRNA compared to manual mixing (Supplementary Fig. 39d). In DC2.4 cells, all microfluidically formulated LNPs exhibited significantly lower FLuc mRNA transfection efficiency than their manually mixed counterparts (Supplementary Fig. 40a). A similar phenomenon was also observed in HEK293T cells, except for pDLS5-LNPs (Supplementary Fig. 40b). LNPs formulated via pipette or microfluidic mixing were not toxic to HEK293T and DC2.4 cells (Supplementary Fig. 40c, d). Additionally, cytotoxicity assessments in DC2.4 and HEK293T cells treated with LNPs loaded with cyanine5 (Cy5)-labeled mRNA at high doses (1000 ng/well and 2000 ng/well, 24-well plate) demonstrated that both pDLS‑LNPs and ALC-LNPs exhibited a favorable in vitro safety profile (Supplementary Fig. 41a, b).

To explore whether polypeptide-engineered LNPs can be used for small interfering RNA (siRNA) delivery. siRNA for silencing green fluorescent protein (GFP) (siGFP) was loaded into LNPs via pipette mixing. All formulations, including ALC-LNP, pDLS2-LNP, and pDLS5-LNP, showed nanosizes (<200 nm), low polydispersity indices (PDI < 0.2), and near-neutral zeta potentials (within ±10 mV). Notably, both pDLS2-LNP and pDLS5-LNP exhibited much higher siGFP encapsulation efficiency compared to ALC-LNP (Supplementary Fig. 42). To evaluate *GFP* gene knockdown efficiency, LNP-mediated gene knockdown in GFP-expressing HEK293 (HEK293-GFP) cells was studied. The HEK293-GFP cells were incubated with LNPs containing siGFP for 48 h. siGFP-loaded LNPs formulated with pDLS2 and pDLS5 achieved significantly lower GFP expression compared to PEGylated ALC-LNP, suggesting more effective siRNA delivery using pDLS-LNPs (Supplementary Fig. 43a). Additionally, siGFP-loaded pDLS-LNPs were not toxic to HEK293-GFP cells (Supplementary Fig. 43b).

Next, we investigated the feasibility of pDLS-LNP formulations for the delivery of a codon-optimized mRNA vaccine candidate encoding the spike glycoprotein of SARS-CoV-2. SARS-CoV-2 spike mRNA was encapsulated into pDLS-LNPs using microfluidic mixing. DLS analysis showed that, like ALC-LNPs, pDLS-LNPs had sub-100-nm diameters with low polydispersity (PDI < 0.14) (Fig. 2c–h and Supplementary Fig. 44), favorable size distributions without multiple peaks or large aggregates (Supplementary Fig. 45), and near-neutral zeta potentials (−5.5 mV to +5.5 mV) (Fig. 2i). The structure of ALC-LNP and representative pDLS-LNP was characterized by cryo-transmission electron microscopy (Cryo-TEM). Cryo-TEM imaging revealed that ALC-LNP displayed an electron-dense core without apparent lamellar ordering, enclosed by a lipid bilayer-like envelope (Fig. 2j). In contrast, both pDLS2-LNP and pDLS4-LNP exhibited a lamellar structure on the surface and a relatively amorphous core, suggesting that these two pDLS-LNPs present similar morphological characteristics (Fig. 2k and Supplementary Fig. 46). As shown in Fig. 2c–h, pDLS-LNPs exhibited larger sizes (~87–100 nm) compared to ALC-LNP (~70 nm), which could be attributed to the fundamental structural differences between PEG and pDLS. PEG adopts a highly flexible random-coil conformation^51,52^, while polypeptides can form more rigid secondary structures due to potential intramolecular hydrogen bonding along their backbone^40,43,53^. This increased chain rigidity may lead to less compact surface packing and, consequently, larger nanoparticle sizes. Moreover, pDLS-LNPs exhibited comparable encapsulation efficiency of SARS-CoV-2 spike mRNA to ALC-LNP (Fig. 2l). These results reveal that the micfluidic co-assembly of pDLS lipids with the ionizable lipid, helper lipid, cholesterol, and mRNA produces uniform LNPs with a core-shell structure. Importantly, SARS-CoV-2 spike mRNA-loaded pDLS-LNPs achieved significantly higher spike protein expression in DC2.4 cells than ALC-LNPs, without associated cytotoxicity (Fig. 2m, n).

### Cellular uptake and endosomal escape of pDLS-LNPs

**Fig. 3:**
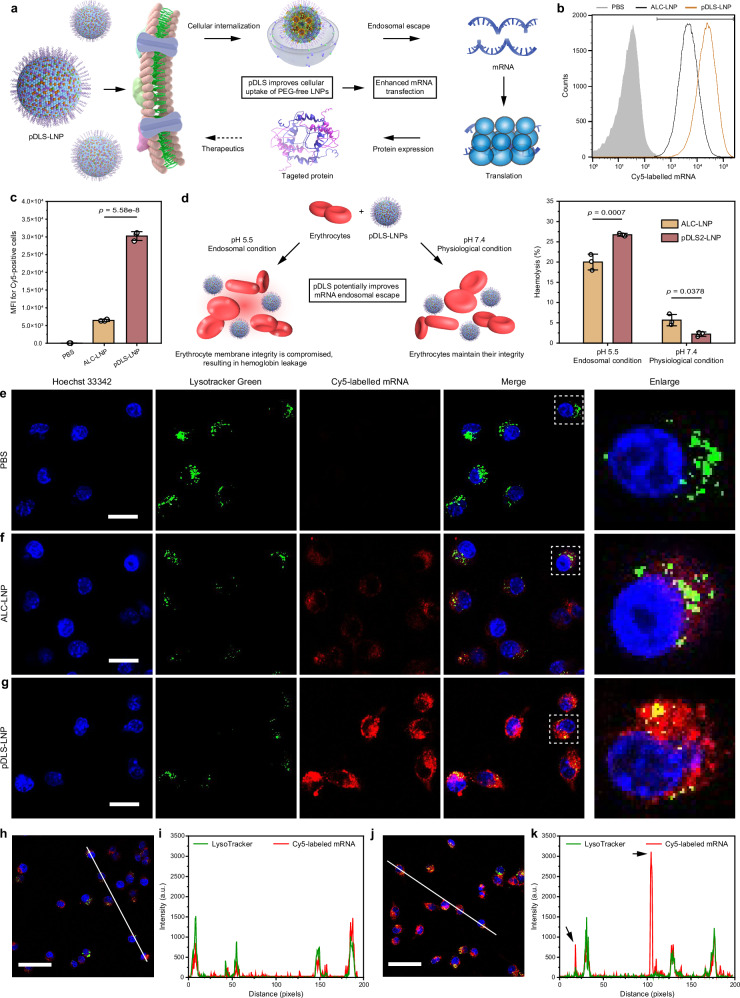
pDLS-LNPs promote cellular internalization and mediate effective endosomal escape for efficient mRNA delivery. **a** Schematic illustrating the cellular uptake, endosomal escape, and intracellular mRNA transfection mediated by pDLS-LNPs. **b** Flow cytometry analysis of cellular uptake in DC2.4 cells treated with representative pDLS2-LNP (Cy5-labeled mRNA-loaded LNPs were prepared via pipette mixing). **c** Quantification of LNP internalization based on flow cytometry data (*n* = 3 independent biological samples; mean ± SD). **d** Hemolysis assay assessing membrane disruption induced by ALC-LNP and pDLS2-LNP in mouse red blood cells at pH 5.5 and pH 7.4, respectively (*n* = 3 independent biological samples; mean ± SD). Representative confocal images of DC2.4 cells treated with **e** PBS, **f** PEGylated ALC-LNP, and **g** PEG-free pDLS2-LNP (Cy5-labeled mRNA-loaded LNPs were prepared via pipette mixing). DC2.4 cells treated with LNPs encapsulated with Cy5-labeled mRNA (Red) for 4 h, followed by staining with LysoTracker Green (Green) and Hoechst 33342 (Blue). Scale bar, 20 μm. Colocalization analysis of mRNA with endosomes using confocal microscopy: **h** Representative image of cells treated with ALC-LNP. Scale bar, 50 μm; **i** Fluorescence intensity profiles showing colocalization between endosomes (Green) and Cy5-labeled mRNA (Red) along the white line in (**h**); **j** Representative image of cells treated with pDLS-LNP. Scale bar, 50 µm; **k** Fluorescence intensity profiles corresponding to (**j**). Arrows indicate the Cy5-labeled mRNA released from the endosomes. In (**e**–**g**), (**h**), and (**j**), each experiment was repeated three times independently with similar results, and a representative result is shown for each. Statistical analyses were performed using two-tailed one-way ANOVA with Tukey’s correction (**c**, **d**). Source data are provided as a Source Data file.

Motivated by the high in vitro transfection efficiency of pDLS-LNPs, we next explored their cellular internalization and endosomal escape using flow cytometry and confocal laser scanning microscopy (CLSM), respectively (Fig. 3a). Both pDLS2-LNP and ALC-LNPs were formulated with Cy5-labeled mRNA to enable tracking of intracellular uptake in DC2.4 cells. Flow cytometric analysis of mean fluorescence intensity (MFI) revealed that pDLS-LNP exhibited approximately 4.6-fold enhancement in uptake efficiency compared with ALC-LNP (Fig. 3b, c), suggesting enhanced internalization by dendritic cells (DCs), probably due to the higher cell adhesion property of polypeptides relative to PEG^46^. The endosomolytic activity of ALC-LNP and pDLS2-LNP was evaluated using a hemolysis assay, where hemoglobin release from red blood cells (RBCs) serves as a proxy for membrane disruption (Fig. 3d). This surrogate assay was used due to the comparable lipid bilayer (phospholipid and cholesterol) and glycocalyx compositions shared between RBCs and endosomal membranes^54,55^. It should be noted, however, that the model has inherent limitations, as endosomal and RBC membranes differ in protein content and RBCs contain a membrane skeleton absent in endosomes. This assay was conducted at both physiological pH (7.4) and endosomal pH (5.5). Hemolysis observed at pH 7.4 reflects potential toxicity, whereas lysis at pH 5.5 serves as an indicator of LNP-mediated membrane disruption under acidic conditions, modeling escape potential from acidic vesicles such as endosomes and lysosomes^56^. At pH 5.5, pDLS2-LNP exhibited significantly higher hemolytic activity than ALC-LNP, underscoring its enhanced membrane-lytic potential. Both ALC-LNP and pDLS2-LNP showed negligible hemolysis at physiological pH, suggesting good cytocompatibility (Fig. 3d). CLSM images demonstrated that DC2.4 cells treated with pDLS2-LNP displayed more diffuse and brighter Cy5-labeled mRNA fluorescence throughout the cytoplasm compared to those treated with ALC-LNP (Fig. 3e–g). The intensity-based pixel matching colocalization analysis of red fluorescence signal (Cy5-mRNA) and green fluorescence signal (LysoTracker Green) showed the distinct distribution of Cy5-mRNA outside the endosomes in the cells treated with pDLS2-LNP for 4 h, consistent with successful Cy5-mRNA release from the endosomes to the cytoplasm (Fig. 3h–k). To further evaluate spatial correlation between Cy5-labeled mRNA and endosomal compartments, we performed pixel-wise colocalization analysis across multiple independent images, and Pearson’s correlation coefficient was calculated using ImageJ/Fiji (Supplementary Fig. 47). It was found that pDLS2-LNP exhibited a lower Pearson’s R value (no threshold) of 0.55 ± 0.05 compared to 0.64 ± 0.06 for ALC-LNP, indicating a reduced degree of colocalization and thus supporting enhanced endosomal escape of Cy5-mRNA by pDLS-LNPs relative to ALC-LNPs. These findings demonstrate that pDLS-LNP not only facilitates superior cellular uptake but also achieves effective endosomal escape, resulting in enhanced intracellular mRNA transfection compared to ALC-LNP (Fig. 2a, m).

### In vivo mRNA delivery performance of pDLS-LNPs

**Fig. 4:**
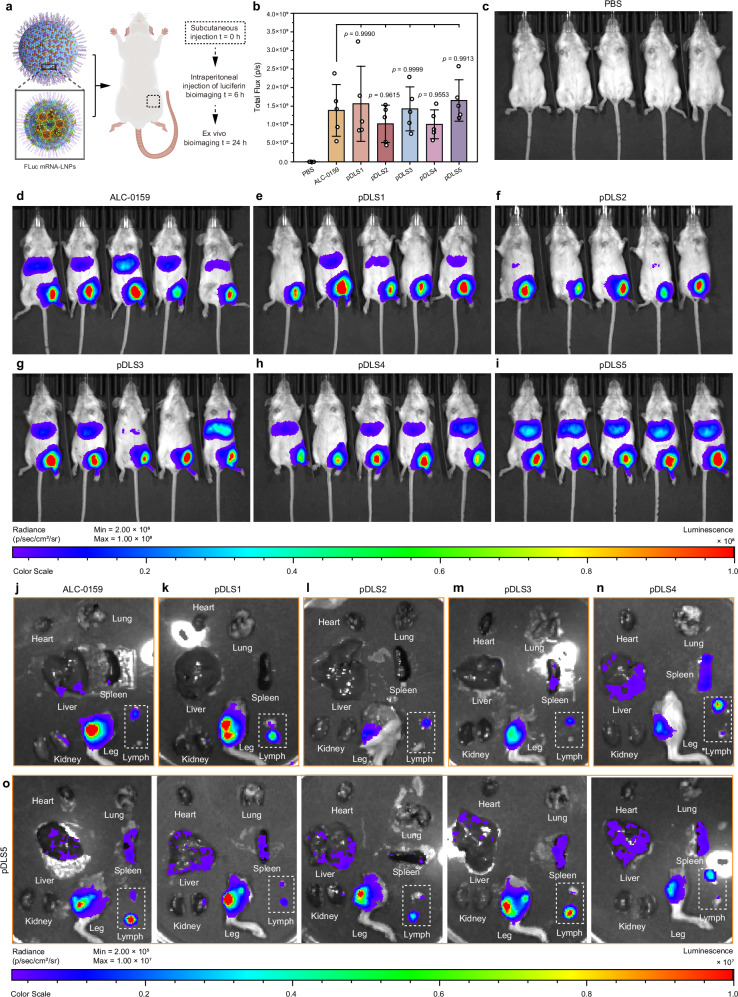
pDLS-LNPs enable efficient mRNA delivery in vivo. **a** Schematic of the experimental design for evaluating in vivo transfection efficiency via bioluminescence imaging after s.c. injection of FLuc mRNA-loaded pDLS-LNPs prepared via microfluidic mixing. **b** Quantification of relative bioluminescence radiance intensity (RBRI) at the injection site of mice treated with different FLuc mRNA-LNPs at 6 h post-s.c. administration (*n* = 5 independent biological samples; mean ± SD). In vivo bioluminescence images at 6 h post-s.c. injection: **c** PBS; **d** PEGylated ALC-LNP; **e** pDLS1-LNP; **f** pDLS2-LNP; **g** pDLS3-LNP; **h** pDLS4-LNP; **i** pDLS5-LNP (*n* = 5 mice per group). Representative ex vivo bioluminescence images of major organs of mice treated with FLuc mRNA-loaded LNPs at 24 h after s.c. administration: **j** PEGylated ALC-LNP; **k** pDLS1-LNP; **l** pDLS2-LNP; **m** pDLS3-LNP; **n**, pDLS4-LNP; **o** pDLS5-LNP. Statistical analyses were performed using two-tailed one-way ANOVA with Tukey’s correction (**b**). Source data are provided as a Source Data file.

To explore the in vivo transfection profiles of pDLS-LNPs, BALB/c mice were injected subcutaneously (s.c) in the left hind leg with FLuc mRNA-loaded pDLS-LNPs (Fig. 4a). FLuc mRNA-mediated luciferase expression was then visualized using bioluminescence imaging at 6 and 24 h post-treatment, with relative bioluminescence radiance intensity (RBRI) measured at the injection site. The RBRI values of PEG-free LNPs formulated with pDLS lipids were comparable to those of the PEGylated ALC-LNP (Fig. 4b), indicating efficient local mRNA transfection. Whole-body images of mice showed that most of the pDLS-LNPs exhibited similar bioluminescence distribution patterns with ALC-LNP (Fig. 4c–i). Specifically, ALC-LNP and pDLS5-LNP displayed widespread bioluminescence, with high signals both locally at the injection site and systemically in other areas (Fig. 4d, i). Moreover, we performed ex vivo bioluminescence imaging of ALC-LNP and pDLS-LNPs at 24 h post-administration to evaluate the mRNA transfection profiles in different organs. Notably, most pDLS-LNPs mediated luciferase expression at the injection site, liver, spleen, and lymph nodes, exhibiting a biodistribution profile comparable to PEGylated ALC-LNP (Fig. 4j–o and Supplementary Fig. 48). This suggests that pDLS is promising as a potential replacement for PEGylated lipids in LNP formulations. Interestingly, pDLS-LNPs formulated with pDLS lipids with lower DPs, such as pDLS2, showed low luciferase expression in the liver of mice. Importantly, the accumulation of pDLS-LNPs in the spleen and lymph nodes may facilitate their uptake into DCs, improving the antigen presentation and immune response activation^57^.

**Fig. 5:**
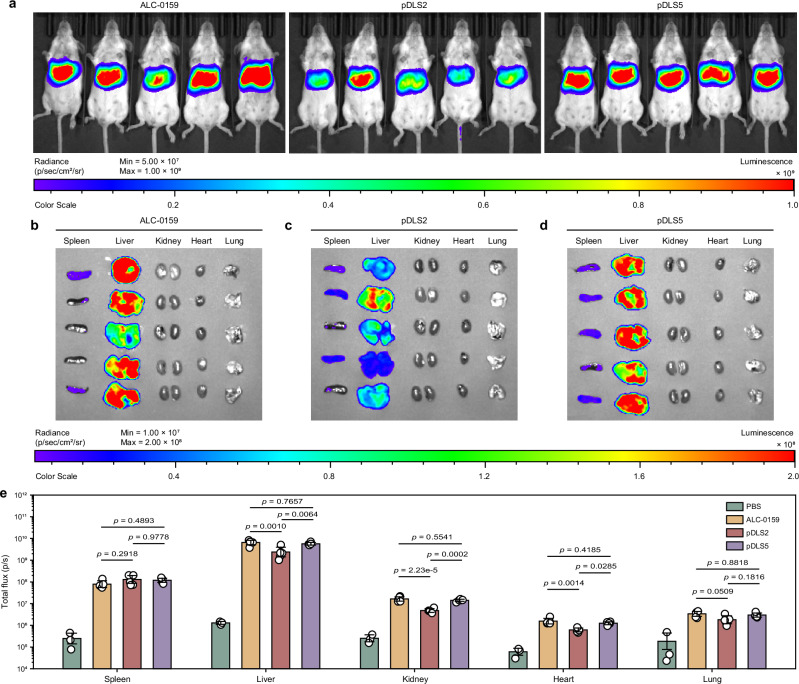
Systemic mRNA delivery using pDLS-LNPs via i.v. injection. **a** In vivo bioluminescence images at 6 h after i.v. injection of FLuc mRNA-loaded pDLS-LNPs (*n* = 3 mice for PBS; *n* = 5 mice for PEGylated ALC-LNP, pDLS2-LNP and pDLS5-LNP groups; FLuc mRNA-loaded LNPs were prepared via microfluidic mixing). Ex vivo bioluminescence images of major organs of mice treated with FLuc mRNA-loaded LNPs at 6 h after i.v. administration (*n* = 3 mice for PBS; *n* = 5 mice for PEGylated ALC-LNP, pDLS2-LNP and pDLS5-LNP groups): **b** PEGylated ALC-LNP; **c** pDLS2-LNP; **d** pDLS5-LNP (*n* = 5). **e** Quantification of the relative bioluminescence radiance intensity (RBRI) in the major organs, including spleen, liver, kidneys, heart, and lungs at 6 h post-i.v. administration (*n* = 3 mice for PBS; *n* = 5 mice for PEGylated ALC-LNP, pDLS2-LNP and pDLS5-LNP groups; mean ± SD). Statistical analyses were performed using two-tailed one-way ANOVA with Tukey’s correction (**e**). Source data are provided as a Source Data file.

To evaluate whether pDLS lipids support systemic mRNA delivery, we further investigated transfection following intravenous (i.v.) administration of FLuc mRNA-loaded LNPs. Representative formulations containing pDLS lipids with low (pDLS2) and high (pDLS5) DPs were selected for comparison with PEGylated ALC-LNP. Based on the whole-body imaging, pDLS-LNPs (both pDLS2-LNP and pDLS5-LNP) exhibited bioluminescence distribution patterns comparable to ALC-LNP at 6 h post-injection (Fig. 5a and Supplementary Fig. 49). Quantification of RBRI demonstrated that pDLS5-LNP achieved systemic transfection efficiency equivalent to that of ALC-LNP (Supplementary Fig. 50). We further performed ex vivo bioluminescence imaging of harvested major organs at 6 h to assess mRNA transfection profiles and tissue distribution (Fig. 5b–d and Supplementary Fig. 51). Similar to ALC-LNP, pDLS-LNPs primarily mediated luciferase expression in the liver and spleen (Fig. 5b–d). Further RBRI analysis indicated that pDLS5-LNP-mediated hepatic expression at a level comparable to ALC-LNP, but significantly higher than that of pDLS2-LNP (Fig. 5e). In the spleen, both pDLS-LNPs showed RBRI levels similar to ALC-LNP (Fig. 5e). In the heart, kidneys, and lungs, the RBRI values of ALC-LNP were comparable to those of pDLS5-LNP, but significantly exceeded the levels observed with pDLS2-LNP (Fig. 5e). These results demonstrate that pDLS lipids can effectively substitute PEGylated lipids in LNP formulations while maintaining efficient systemic mRNA delivery with performance dependent on pDLS chain length.

### Ex vivo biodistribution evaluation of pDLS-LNPs

**Fig. 6:**
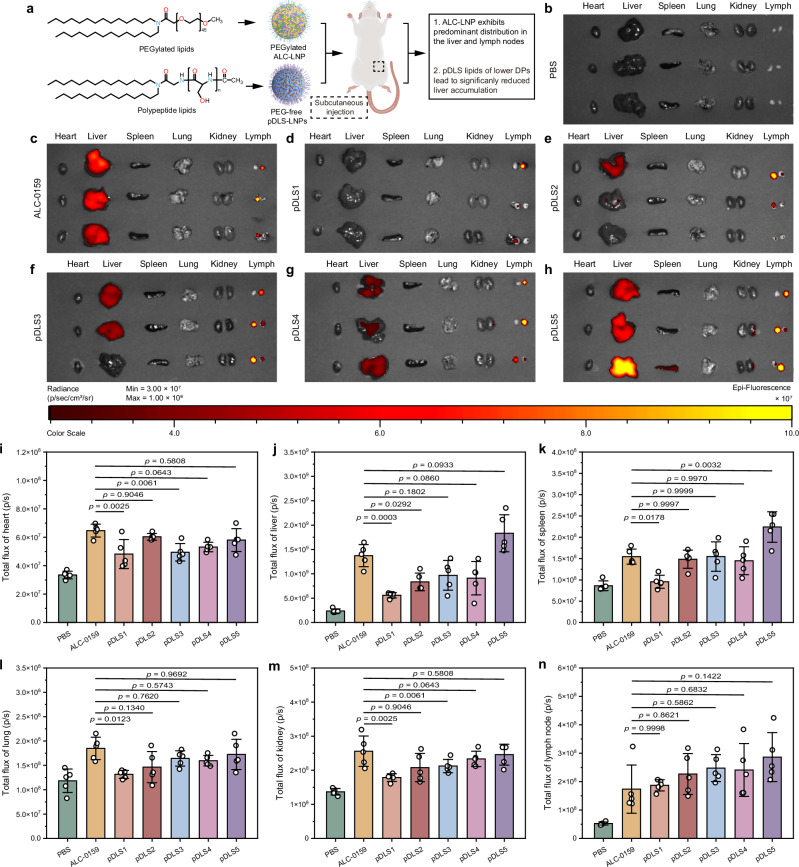
Ex vivo biodistribution of pDLS-LNPs. **a** Schematic illustration of the biodistribution assessment of mRNA-loaded pDLS-LNPs in major organs. Representative ex vivo near-infrared fluorescence images of major organs collected at 6 h after s.c. injection of mRNA-LNPs labeled with DiR′ (*n* = 5 mice per group, LNPs were prepared via microfluidic mixing): **b** PBS; **c** ALC-LNP; **d** pDLS1-LNP; **e** pDLS2-LNP; **f** pDLS3-LNP; **g** pDLS4-LNP; **h** pDLS5-LNP. Quantification of the relative fluorescence radiance intensity (RFRI) in the **i** heart; **j** liver; **k** spleen; **l** lungs; **m** kidneys; **n** lymph nodes at 6 h post-administration of PBS, PEGylated ALC-LNP, pDLS1-LNP, pDLS2-LNP, pDLS3-LNP, pDLS4-LNP or pDLS5-LNP (*n* = 5 independent biological samples; mean ± SD). Statistical analyses were performed using two-tailed one-way ANOVA with Tukey’s correction (**i**–**n**). Source data are provided as a Source Data file.

To further assess the organ-level distribution of pDLS-LNPs, we performed ex vivo near-infrared fluorescence imaging using mRNA-LNPs labeled with the lipophilic fluorescence dye, 1,1′-dioctadecyl-3,3,3′,3′-tetramethylindotricarbocyanine iodide (DiR′) at 6 h after s.c. injection (Fig. 6a). Ex vivo fluorescence images showed that PEGylated ALC-LNP accumulated predominantly in both the liver and lymph nodes (Fig. 6b, c). Similarly, the PEG-free LNPs, especially pDLS3-, pDLS4-, and pDLS5-LNPs, exhibited substantial accumulation in these organs (Fig. 6d–h). The extent of organ accumulation was analyzed by examining the relative fluorescence radiance intensity (RFRI). While its RFRI in the heart was comparable with pDLS2-LNP, pDLS4-LNP, and pDLS5-LNP, ALC-LNP exhibited significantly higher RFRI than pDLS1-LNP and pDLS3-LNP (Fig. 6i). In the liver, ALC-LNP showed similar RFRI to pDLS3-LNP, pDLS4-LNP, and pDLS5-LNP, but significantly exceeded the levels observed for pDLS1-LNP and pDLS2-LNP (Fig. 6j). Notably, the accumulation of pDLS-LNPs in the liver increased with higher DPs of pDLS. Among all groups, pDLS5-LNP demonstrated significantly higher RFRI in the spleen than ALC-LNP (Fig. 6k), while RFRI in the lungs and kidneys was largely comparable across formulations, except for the lower levels seen with pDLS1-LNP (Fig. 6l, m). In the lymph nodes, pDLS-LNPs generally showed relatively higher RFRI than ALC-LNPs (Fig. 6n). These findings were in agreement with the bioluminescence imaging results. Notably, the reduced liver accumulation of pDLS1- and pDLS2-LNPs suggests a potentially lower risk of hepatotoxicity, particularly with repeated dosing.

### Evaluation of cellular and humoral immune responses induced by SARS-CoV-2 spike mRNA-loaded pDLS-LNPs

**Fig. 7:**
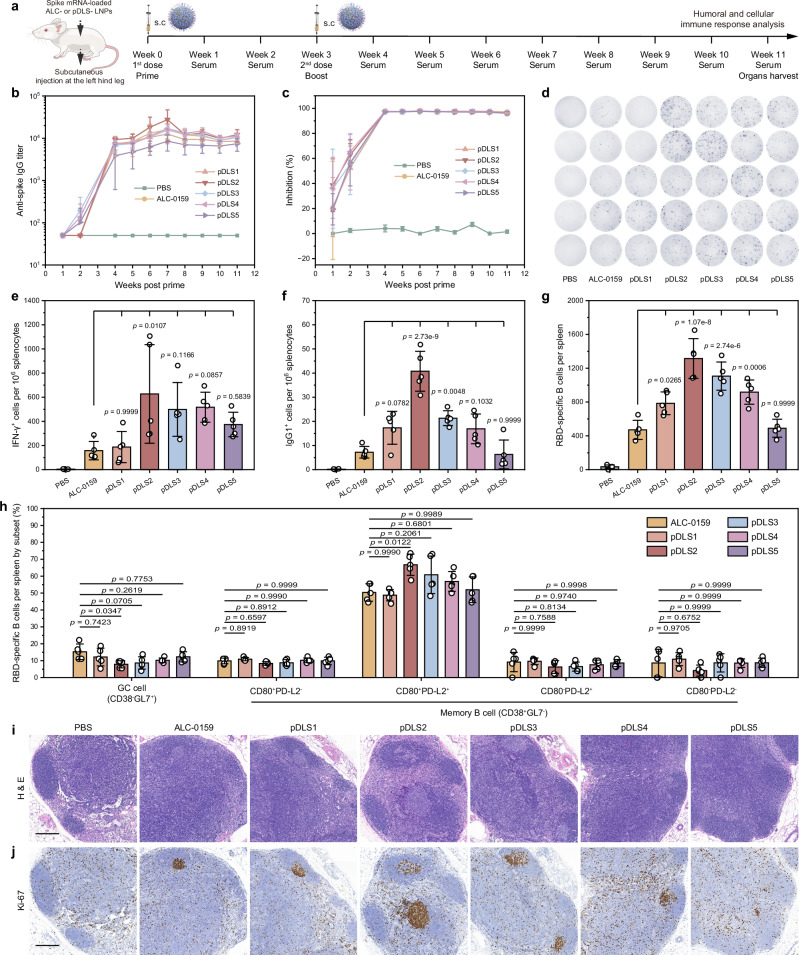
pDLS-LNPs encapsulated with SARS-CoV-2 mRNA vaccines elicit robust and durable cellular and humoral immune responses in mice. **a** Schematic of the prime-boost immunization strategy. BALB/c mice were s.c. immunized with SARS-CoV-2 mRNA-loaded LNPs (containing 3 μg mRNA) at week 0 and 3 (SARS-CoV-2 mRNA-loaded LNPs were prepared via microfluidic mixing). Sera were collected weekly to assess anti-spike IgG titers and neutralizing antibody responses. Lymph nodes and spleens were harvested at the end of the experiment for analysis of immune cell proliferation, antigen-specific T and B-cell responses, respectively. **b** Serum anti-spike IgG titers (*n* = 5 independent biological samples; mean ± SD). **c** Serum neutralizing antibody activity (*n* = 5 independent biological samples; mean ± SD). **d** ELISpot images showing SARS-CoV-2 spike-specific IFN-γ^+^ cells in spleens. **e** ELISpot quantification of SARS-CoV-2 spike-specific IFN-γ^+^ cells in spleens (*n* = 5 independent biological samples; mean ± SD). **f** Quantification of IgG1-secreting B cells in spleens by ELISpot (*n* = 5 independent biological samples; mean ± SD). **g** Number of RBD-specific B cells per spleen (*n* = 5 independent biological samples; mean ± SD). **h** Percentage of RBD-specific germinal center (GC) B cells and memory B cells in spleens (*n* = 5 independent biological samples; mean ± SD). Memory B cells were further classified based on PD-L2 and CD80 expression. **i** Representative H&E staining images of lymph nodes (Scale bar, 200 μm). **j** Representative Ki-67 immunohistochemical staining images of lymph nodes (Scale bar, 200 μm). In (**i**) and (**j**), each experiment was repeated twice independently with similar results, and a representative result is shown for each. Statistical analyses were performed using two-tailed one-way ANOVA with Tukey’s correction (**e**–**h**). Source data are provided as a Source Data file.

Given the promising in vitro and in vivo mRNA delivery performance of pDLS-LNPs, we evaluated their potential as carriers for a SARS-CoV-2 mRNA vaccine candidate. BALB/c mice were s.c. vaccinated following a prime-boost regimen with a 3-week interval. Serum, spleen, and lymph nodes were collected to evaluate both cellular and humoral immune responses after vaccination (Fig. 7a). Vaccination with SARS-CoV-2 spike mRNA-loaded pDLS-LNPs triggered robust anti-spike IgG responses (Fig. 7b and Supplementary Figs. 52 and 53) and high levels of neutralizing antibody activity (Fig. 7c), which were persistent over 11 weeks and comparable to ALC-LNP. Meanwhile, there were no remarkable body weight changes observed throughout the vaccination study (Supplementary Fig. 54), demonstrating good tolerability of pDLS-LNPs. SARS-CoV-2 spike-specific type 1 helper T cell immune response was assessed by using an interferon-γ (IFN-γ) ELISpot assay. pDLS2-LNP elicited significantly higher IFN-γ secretion than ALC-LNP (Fig. 7d, e). In addition, IgG1 ELISpot analysis revealed that mRNA-LNPs formulated with pDLS2 or pDLS3 induced significantly larger numbers of IgG1-secreting B cells than ALC-LNP (Fig. 7f and Supplementary Fig. 55), suggesting superior induction of memory B-cell response than ALC-LNP^58^. These findings likely result from the greater cellular uptake and enhanced endosomal escape of mRNA-loaded pDLS-LNPs (Fig. 3), together with the absence of anti-pDLS immune responses, as discussed later.

To further evaluate the induction of antigen-specific B-cell response, we quantified SARS-CoV-2 receptor-binding domain (RBD)-specific B cells and memory B cells by flow cytometry using fluorescently labeled RBD probes (Supplementary Fig. 56)^59^. All pDLS-LNP, except pDLS5, significantly increased the number of RBD-specific B cells compared to ALC-LNP (Fig. 7g and Supplementary Figs. 57–63). Notably, pDLS1-, pDLS2-, pDLS3-, and pDLS4-LNPs stimulated a significant increase of 1.7-, 2.8-, 2.4-, and 1.9-fold, respectively, over ALC-LNP. Most RBD-specific B cells exhibited a memory phenotype, co-expressing the memory markers PD-L2 and CD80 (Fig. 7h). This suggests their potential for rapid differentiation into antibody-secreting cells upon antigen reexposure^60^. Thus, it is conceivable that SARS-CoV-2 spike mRNA-loaded pDLS-LNPs elicited a larger, yet phenotypically similar, population of RBD-specific memory B cells compared to ALC-LNP. We also performed immunohistochemical analysis of the cell proliferation marker Ki-67 to assess the cell proliferation in the lymph nodes at the endpoint^61^ (Fig. 7i, j). Compared to ALC-LNP, pDLS-LNPs induced greater cell proliferation in lymph nodes after vaccination, potentially indicating their greater capacity to promote T cell and B-cell activation. Altogether, these results demonstrate that SARS-CoV-2 spike mRNA-loaded pDLS-LNPs could induce robust antigen-specific cellular and humoral immune responses.

### In vivo safety and storage performance of mRNA-loaded pDLS-LNPs

**Fig. 8:**
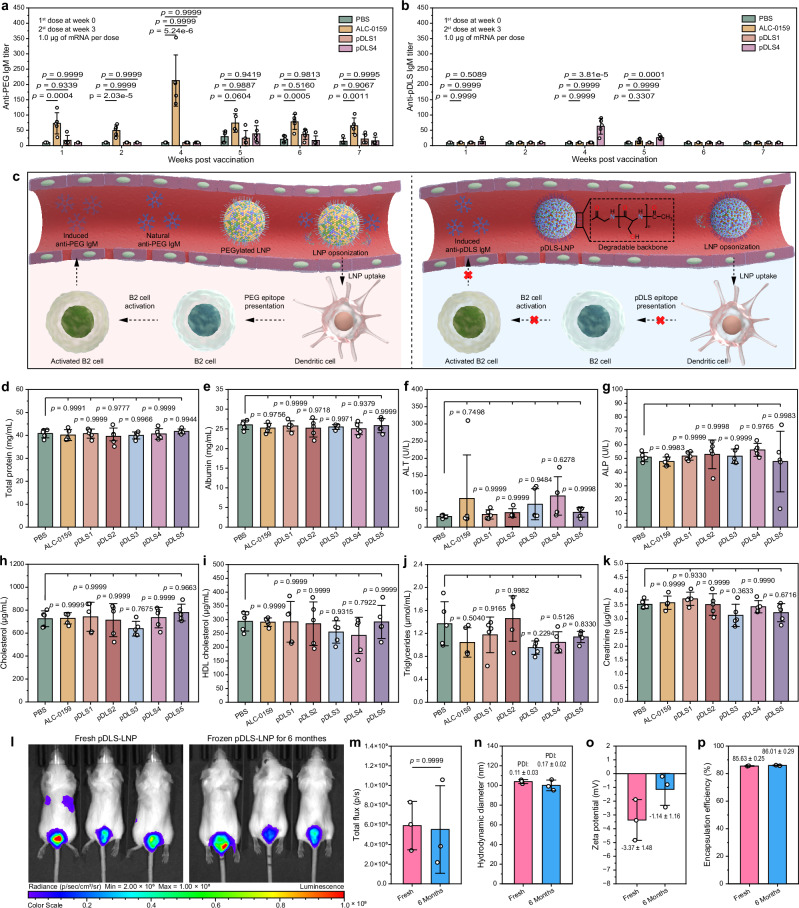
pDLS-LNPs enhance in vivo safety and long-term storage stability of PEG-free formulations. **a** Anti-PEG IgM titers following s.c. administration of PEGylated ALC-LNP and PEG-free pDLS-LNPs (pDLS1-LNP and pDLS4-LNP) over time (*n* = 5 independent biological samples; mean ± SD; SARS-CoV-2 mRNA-loaded LNPs were prepared via microfluidic mixing). **b** Anti-pDLS IgM titers in mice treated with ALC-LNP or pDLS-LNPs over time (*n* = 5 independent biological samples; mean ± SD; SARS-CoV-2 mRNA-loaded LNPs were prepared via microfluidic mixing). **c** Schematic illustrating reduced immunogenicity of PEG-free pDLS-LNPs compared to PEGylated counterparts. PEG stimulates B2 cell activation and anti-PEG IgM production, whereas pDLS-LNPs minimize this response against pDLS. Blood biochemical analysis of safety biomarkers in treated mice (*n* = 5 independent biological samples; mean ± SD), including: **d** total protein; **e** albumin; **f** ALT; **g** ALP; **h** cholesterol; **I** HDL cholesterol; **j** triglycerides; **k** creatinine. **l** In vivo transfection analysis based on bioluminescence imaging for FLuc mRNA-loaded pDLS-LNP after 6 months of storage at −80 °C (FLuc mRNA-loaded LNPs were prepared via microfluidic mixing). **m** Quantification of RBRI at the injection site of mice (*n* = 3 mice per group). Physicochemical properties of mRNA-loaded pDLS-LNPs after 6 months of storage at −80 °C: **n** Hydrodynamic diameter and PDI (*n* = 3 independent samples; mean ± SD); **o** zeta potential (*n* = 3 independent samples; mean ± SD); **p** mRNA encapsulation efficiency (*n* = 2 independent samples; mean ± SD). Statistical analyses were performed using two-tailed one-way ANOVA with Tukey’s correction (**a**, **b**, and **d**–**k**) and unpaired two-tailed Student’s *t*-test with Mann-Whitney test (**m**). Source data are provided as a Source Data file.

Growing evidence reveals that the anti-PEG antibodies present in humans may cause adverse effects upon administration of PEGylated therapeutics^27^. PEG, a synthetic hydrophilic polymer, is commonly used in everyday consumer products (e.g., cosmetics, personal care items) and in pharmaceutical formulations, including LNP-based SARS-CoV-2 mRNA vaccines^62^. To evaluate immunogenicity, we compared anti-PEG and anti-polyserine immune responses induced by PEGylated ALC-LNP and PEG-free pDLS-LNPs in mice. Anti-PEG IgM titer assay revealed that mice injected with ALC-LNP containing 1 µg of mRNA produced significantly elevated anti-PEG IgM titers, particularly at one week after the second dose (Fig. 8a). In contrast, mice treated with PEG-free pDLS1-LNP or pDLS4-LNP (containing 1 μg mRNA) exhibited minimal immune responses. While pDLS4-LNP induced relatively weak yet detectable levels of anti-pDLS IgM, pDLS1-LNP elicited no detectable anti-pDLS IgM (Fig. 8b). We further assessed the anti-pDLS IgM levels in mice immunized with pDLS2-LNP (3 µg mRNA), which previously demonstrated superior immunogenicity, including enhanced IFN-γ secretion (Fig. 7d, e), increased numbers of IgG1-secreting (Fig. 7f) and RBD-specific (Fig. 7g) B cells relative to ALC-LNP. Notably, no anti-pDLS2 IgM was detected in the sera of immunized mice at week 4 (one week after the second dose) (Supplementary Fig. 64). This may be attributed to PEG-free pDLS1- and pDLS2-LNPs formulated with biodegradable polypeptide-based lipids, avoiding the production of polypeptide-specific antibodies (Fig. 8c)^28^. Additionally, no changes in body weight were observed throughout the experiment, further supporting the tolerability of pDLS-LNP (Supplementary Fig. 65). These results indicated that the use of pDLS lipids to fabricate PEG-free LNPs can avoid the production of anti-PEG IgM and thus mitigate the risk of PEG-associated immune responses observed with the existing mRNA-LNP vaccines.

To further assess safety, we conducted blood biochemistry analysis to evaluate potential effects on liver, heart, and kidney functions. Serum levels of total protein, albumin, alanine aminotransferase (ALT), alkaline phosphatase (ALP), cholesterol, high-density lipoprotein (HDL) cholesterol, triglycerides, and creatinine were comparable across all groups, including those treated with pDLS-LNPs and PBS (Fig. 8d–k). Histological analysis of the heart, lungs, liver, spleen, and kidneys showed no evidence of organ injury, necrosis, or any abnormal morphological changes, further confirming the safety of pDLS-LNPs (Supplementary Fig. 66). Finally, we investigated the storage stability of PEG-free pDLS-LNPs through freezing. LNPs formulated with pDLS lipid with a DP of 23 (comparable to pDLS2) were used as a representative formulation. pDLS-LNP demonstrated promising cryopreserved stability even after 6 months of storage at -80 °C; good size distribution, near-neutral zeta potential, comparable encapsulation efficiency, and effective in vivo transfection compared with fresh pDLS-LNP (Fig. 8l–p and Supplementary Fig. 67). These results revealed that PEG-free pDLS-LNPs can effectively maintain their physiochemical properties and bioactivity during 6-month storage at −80 °C.

In summary, we have successfully synthesized a series of hydrophilic, nonionic, and biodegradable pDLS lipids for fabricating PEG-free LNPs. The optimized LNPs formulated with pDLS lipids by microfluidic mixing exhibited desirable physicochemical properties, including nanosize with low polydispersity, near-neutral zeta potential, high encapsulation efficiency, and excellent colloidal stability. Leveraging the inherent functionality of polypeptides, pDLS-LNPs demonstrated enhanced cellular uptake and efficient endosomal escape in DCs, resulting in significantly enhanced mRNA transfection efficiency compared to PEGylated ALC-LNP used in the BNT162b2 vaccine. SARS-CoV-2 spike mRNA-loaded pDLS-LNPs elicited robust and sustained cellular and humoral immune responses, without inducing systemic toxicity. Unlike ALC-LNP, repeated administration of pDLS-LNPs showed minimal anti-pDLS immunogenicity, addressing a major limitation of current PEGylated LNP platforms. Moreover, pDLS-LNP maintained their physiochemical properties and bioactivity for at least 6 months at −80 °C. Collectively, our findings highlight pDLS-LNPs as a safe, effective, and storage-stable platform for mRNA delivery, with potential for translation into next-generation mRNA vaccines and therapeutics.

## Methods

### Cell lines and mice

The mouse dendritic cell line DC2.4 (Merck), the human embryonic kidney cell line HEK293T (ATCC), and the HEK293-GFP stable cells (GeneTarget) were maintained in RPMI 1640 (DC2.4) and Dulbecco’s modified Eagle’s medium (DMEM, HEK293T, HEK293-GFP), respectively. Both media were supplemented with 10% FBS and 1% penicillin-streptomycin. BALB/c mice, obtained from InVivos Pte. Ltd., were housed in a specific-pathogen-free facility and cared for in compliance with institutional and international guidelines for the ethical use of laboratory animals. The facility has a 12-h light/dark cycle, with a standard environmental temperature of 20–24 °C and humidity of 40–60%. All animal study procedures were approved by the Institutional Animal Care and Use Committee of the Biological Resource Center (BRC), A*STAR, under protocol number: 221681 and its renewed approval: 251905.

### mRNA-loaded LNP formulation and characterization

The pDLS lipids were dissolved in a 9:1 ethanol to nuclease-free water (Promega) mixture at 3 mg ml^−1^. ALC-0315, DSPC, cholesterol, and ALC-0159 (all from MedChemExpress) were each dissolved in ethanol at concentrations of 20 mg ml^−1^, 10 mg ml^−1^, 10 mg ml^−1^, and 3 mg ml^−1^, respectively. The FLuc mRNA (TriLink) and the SARS-CoV-2 mRNA vaccine candidate PVX1010 (Provaxus, Inc.) were prepared in 1 mM sodium citrate buffer (pH 6.4) at 1 mg ml^−1^. For in vitro studies, LNPs were formulated using manual pipette mixing. Specifically, the ethanol phase containing ALC-0315, DSPC, cholesterol, and either pDLS or ALC-0159 at defined molar ratios (Supplementary Table 1) was rapidly mixed with an aqueous mRNA solution (10 mM sodium acetate, pH 4.0) at an N/P ratio of 6:1 (N/P 6). The mixture was pipetted up and down 20 times to ensure thorough mixing. For in vivo studies and encapsulation of SARS-CoV-2 mRNA, LNPs were formulated via microfluidic mixing using the NanoAssemblr Ignite nanoparticle formulation microfluidic device (Cytiva). Briefly, the ethanol phase (ALC-0315:DSPC:cholesterol:pDLS or ALC-0159, at a molar ratio of 46.3:9.4:42.7:1.6) was mixed with the mRNA-containing aqueous phase (10 mM sodium acetate, pH 4.0) at N/P 6, using a flow rate ratio of 1:3 (ethanol:aqueous). Following formulation, the LNPs were diluted in PBS (1st BASE) and concentrated using 30 kDa molecular weight cut-off ultrafiltration centrifugal concentrators (Sartorius) for 1 h at 800 × *g* and 4 °C. The hydrodynamic diameter, PDI, and zeta potential were assessed in saline using a Nano ZSE system (Malvern). In addition, the encapsulation efficiency of mRNA was measured using the Quant-iT RiboGreen RNA Assay Kit (Invitrogen), following the manufacturer’s instructions.

### Sample preparation and Cryo-TEM imaging of pDLS-engineered LNP

A 3.5 µl aliquot of ALC-LNP or pDLS-LNP was deposited onto a 300-mesh Quantifoil R1.2/1.3 copper grid coated with ultrathin carbon (Quantifoil Micro Tools GmbH), which had been glow-discharged in air for 60 s (FLuc mRNA-loaded LNPs were prepared via microfluidic mixing). The sample was blotted for 2.5 s at blot force 2 under conditions of 22 °C and 100% humidity, and then rapidly vitrified in liquid ethane using the FEI Vitrobot Mark IV. Cryo-TEM imaging was conducted on a 300 kV Titan Krios electron microscope, equipped with a Selectris X imaging filter and a Falcon 4i direct electron detector. Images were captured at the magnifications of 105,000×, corresponding to pixel sizes of either 1.6 Å/px or 1.2 Å/px.

### In vitro FLuc mRNA and SARS-CoV-2 mRNA delivery

To evaluate the transfection performance of mRNA-loaded pDLS-LNPs in vitro, DC2.4 or HEK293T cells were dispensed into 96-well plates (1 × 10^4^ per well) and incubated overnight. Cells were then treated with LNPs containing 100 ng of FLuc mRNA or SARS-CoV-2 mRNA per well (mRNA-LNPs were prepared via pipette mixing). After 48 h of incubation, luciferase activity was analyzed using ONE-Glo Luciferase Assay System (Promega), while spike protein expression was detected using the SARS-CoV-2 Spike RBD Protein Sandwich ELISA Kit (GeneTex). The viability of cells was determined using the alamarBlue Cell Viability Reagent (Invitrogen). All procedures were conducted according to the manufacturer’s guidelines.

### Cellular uptake

DC2.4 cells were plated in 24-well plates (1 × 10^5^ per well) and incubated overnight to allow for adherence. The next day, the cells were exposed to LNPs containing Cy5-labeled mRNA at a dose of 1000 ng per well (Cy5-labeled mRNA-loaded LNPs were prepared via pipette mixing). Cells were collected after a 24-h incubation and subsequently analyzed on a FACSymphony A3 flow cytometer (BD Biosciences). The resulting data were processed and visualized using the FlowJo software.

### Endosomal escape

DC2.4 cells were cultured overnight on Nunc Lab-Tek 8-well Chamber Slides (Thermo Fisher Scientific) (1 × 10^4^ per well). The next day, cells were incubated with LNPs containing Cy5-labeled mRNA (APExBIO, Cat# R1010) at a total mRNA dose of 1000 ng for 4 h (Cy5-labeled mRNA-loaded LNPs were prepared via pipette mixing). Following treatment, cells were stained with 500 nM LysoTracker Green (Thermo Fisher Scientific) for 1 h to visualize acidic organelles, and then counterstained with 5 μg ml^−1^ Hoechst 33342 (Thermo Fisher Scientific) for 10 min to label nuclei. Images were captured immediately using an FV3000 confocal laser scanning microscope (Olympus), and fluorescence profiles of colocalization were analyzed using the ImageJ software.

### Hemolysis assay for evaluating pH-dependent membrane destabilization by LNPs

To assess the ability of LNPs to disrupt membranes in a pH-dependent manner, a hemolysis assay was performed using mouse red blood cells. Whole blood was collected, and erythrocytes were isolated by centrifugation at 1000 × *g* for 5 min at 4 °C. The cells were washed three times with saline, and the supernatant was discarded after each wash. The resulting pelleted erythrocytes were resuspended in saline and counted. For the assay, erythrocytes were adjusted to 2 × 10⁸ cells ml^-1^ in PBS at either pH 5.5 (mimicking the endosomal environment) or pH 7.4 (physiological pH). Equal volumes of LNP solution and erythrocyte suspension were mixed in 1.5 ml Eppendorf tubes to reach a final lipid concentration of 40 μM. Saline was used as a negative control, while 0.5% Triton X-100 in saline served as a positive control to induce complete hemolysis. All conditions were tested in triplicate. Samples were incubated at 37 °C for 30 min, then centrifuged at 1000 × *g* for 5 min at 4 °C. A 100 µL portion of the resulting supernatant was transferred from each tube into a flat, clear-bottom 96-well plate. Absorbance at 545 nm was recorded using a TECAN plate reader to quantify hemoglobin release. Hemolysis was quantified using the following equation: Hemolysis (%) = [(OD₅₄₅(sample) − OD₅₄₅(negative control))/(OD₅₄₅(positive control) − OD₅₄₅(negative control))] × 100. Higher percentages reflect greater endosomal destabilization potential of the LNPs.

### In vivo bioluminescence imaging of mRNA-loaded LNPs

To evaluate transfection efficiency and biodistribution in vivo, LNPs encapsulating 1 μg of FLuc mRNA were s.c. injected into the left hind legs, or LNPs encapsulating 2 μg of FLuc mRNA (FLuc mRNA-loaded LNPs were prepared via microfluidic mixing) were i.v. injected into the tail of BALB/c mice (6–8 weeks old, female). At 6 h or 24 h post-injection, mice received intraperitoneal (i.p.) injections with 200 µL of 15 mg/mL VivoGlo luciferin (Promega, USA). After 10 min, luminescence signal from mice was detected using the IVIS Spectrum imaging system (PerkinElmer, USA). For biodistribution analysis, mice were administered the same dose of luciferin i.p. at 6 h or 24 h post-LNP injection, just prior to euthanasia. Lymph nodes, spleens, kidneys, livers, hearts, lungs, and left hind legs were harvested and placed into individual wells of 24-well plates. A luciferin solution (300 μg ml^−1^ in PBS) was added to each well until the tissues and organs were fully submerged. Bioluminescence images were captured after 5 min of incubation and analyzed using the Living Image software.

### Ex vivo near-infrared fluorescence imaging of mRNA-loaded LNPs

To enable near-infrared fluorescence tracking, LNPs were labeled with DiR′ lipid dye (Life Technologies) by incorporating it at 0.5 mol% of total lipid content into the ethanol phase prior to microfluidic mixing, ensuring stable dye encapsulation. BALB/c mice (6–8 weeks old, female) were s.c. injected in the left hind leg with mRNA-loaded ALC-, pDLS1-, pDLS2-, pDLS3-, pDLS4- and pDLS5-LNPs (2.0 μg mRNA per mouse; *n* = 5 mice per group). Untreated mice served as the negative control (*n* = 5 mice). At 6 h post-injection, mice were sacrificed, and the heart, lungs, liver, kidneys, spleen, and lymph nodes were collected. Ex vivo near-infrared fluorescence imaging was carried out using an excitation wavelength of 754 nm and an emission wavelength of 800 nm. Quantification of fluorescence signals was conducted using Living Image software (PerkinElmer), and total flux values were measured for each region of interest.

### In vivo immunization

To evaluate immune responses in vivo, BALB/c mice (6–8 weeks old, female) were s.c. injected in the left hind leg with LNPs containing 3 μg of SARS-CoV-2 spike mRNA, following a prime-boost strategy with two doses administered three weeks apart (SARS-CoV-2 spike mRNA-loaded LNPs were prepared via microfluidic mixing). Serum samples were collected every week post-vaccination for ELISA testing and final biochemistry analysis. Throughout the study, body weight was monitored every week. Eight weeks after the booster dose, mice were euthanized for tissue collection. Spleens were harvested for ELISpot assays, flow cytometry, and H&E staining. Lymph nodes were collected for immunohistochemical staining of Ki-67 and H&E staining. Kidneys, livers, lungs, and hearts were collected and analyzed by H&E staining.

### Measurement of anti-spike antibody titers and neutralizing antibody activity

To quantify anti-spike antibody levels, Nunc MaxiSorp 96-well plates (Thermo Fisher Scientific) were prepared by coating them at 4 °C overnight with recombinant SARS-CoV-2 spike protein (GenScript, 100 µg ml^−1^). The following day, plates were blocked with PBS supplemented with 4% BSA for 2 h at 37 °C and rinsed twice with PBS. Serial dilutions of mouse serum were prepared in PBS with 1% BSA and left to incubate at room temperature for 2 h. Following three washes with PBS, an anti-mouse IgG antibody (Bethyl Laboratories, Cat# NC9965958, 1:10000) conjugated to horseradish peroxidase was added, followed by a 1 h incubation. Plates were washed three additional times with PBS, after which 100 µl of KPL SureBlue Reserve TMB substrate was introduced to each well and incubated at room temperature for 15 min to allow color development. The reaction was then terminated by adding an equal volume of KPL TMB Stop Solution. Absorbance at 450 nm was determined using a Spark Multimode Microplate Reader. Antibody titers were determined based on the highest serum dilution yielding an optical density (OD) value greater than three times the background. Samples with OD readings below the detection threshold were assigned an arbitrary value of 50, as titers could not be interpolated. Neutralizing antibody inhibition responses were assessed using the SARS-CoV-2 Surrogate Virus Neutralization Test Kit (GenScript) in accordance with the manufacturer’s instructions.

### ELISpot assay

Spleens were collected and dissociated into single-cell suspensions using RPMI 1640 medium, passed through 70 µm cell strainers (Falcon), and centrifuged at 1000 × *g* for 5 min at 4 °C. Red blood cells were subsequently lysed on ice with ACK Lysing Buffer (Gibco) for 5 min, followed by a wash with PBS containing 0.2% BSA to remove cell debris. The Murine IFN-γ ELISpot Kit (Abcam) was utilized to evaluate IFN-γ secretion. A total of 5 × 10^5^ splenocytes were seeded per well in a 96-well plate pre-coated with capture antibodies and stimulated with 0.03 nmol of PepTivator SARS-CoV-2 Prot_S B.1.617.2 Mutation Pool (Miltenyi Biotec). For the detection of RBD-specific IgG1-secreting cells, the Mouse IgG1 Single-Color ELISpot Kit (ImmunoSpot) was used, and 5×10^5^ cells were initially cultured with CTL Mouse B-Poly-S (ImmunoSpot) for three days. Subsequently, 2 × 10^7^ cells per well were transferred to a 96-well PVDF plate coated with 10 μg ml^−1^ SARS-CoV-2 Spike RBD protein (Sino Biological). All subsequent steps followed the respective manufacturers’ instructions. Upon completion, the plates were dried overnight, scanned, and counted using a Mabtech ELISpot Fluorospot Reader (Biomed Global).

### Flow cytometric analysis of memory B cells

Spleens were collected and processed into single-cell suspensions in RPMI 1640, passed through 70 µm cell strainers, and centrifuged at 1000 × *g* for 5 min at 4 °C. The obtained single-cell suspensions were treated with ACK Lysing Buffer for 1 min on ice to lyse red blood cells, followed by washing with PBS containing 0.2% BSA to remove cell debris. For flow cytometry analysis, 5 × 10^6^ splenocytes per sample were incubated with 5 µg ml^−1^ biotinylated SARS-CoV-2 Spike RBD (Sino Biological) in RPMI 1640 for 1 h at 37 °C with 5% CO_2_, washed with PBS containing 0.2% BSA, and stained with BV421-Streptavidin and APC-Streptavidin (BioLegend) for 30 min. Cell samples were then washed with PBS, blocked with anti-mouse CD16/32 antibodies (BioLegend, Cat# 101301, clone 93, Lot# B460768, 1:100) for 30 min at 4 °C to reduce nonspecific binding, and stained for 1 h at 4 °C using the following antibodies: BV785 anti-mouse B220 (BioLegend, Cat# 103246, clone RA3-6B2, Lot# B379487, 1:25), PE/Cy5 anti-mouse CD19 (BioLegend, Cat# 115510, clone 6D5, Lot# B409651, 1:50), PE/Dazzle594 anti-mouse IgM (BioLegend, Cat# 406530, clone RMM-1, Lot# B365242, 1:25), BV711 anti-mouse IgD (BioLegend, Cat# 405731, clone 11-26c.2a, Lot# B370139, 1:50), AF700 anti-mouse CD38 (BioLegend, Cat# 102742, clone 90, Lot# B380905, 1:100), PE/Cy7 anti-mouse GL7 (BioLegend, Cat# 144619, clone GL7, Lot# B376163, 1:50), BV650 anti-mouse CD80 (BioLegend, Cat# 104732, clone 16-10A1, Lot# B401937, 1:25), and PE anti-mouse PD-L2 (BioLegend, Cat# 107205, clone TY25, Lot# B392057, 1:10). Following staining, cells were washed with PBS containing 0.2% BSA. Cell samples were collected and analyzed using a FACSymphony A3 (BD Biosciences). Flow cytometry data were analyzed using FlowJo software. The gating strategies were provided in Supplementary Fig. 56.

### Evaluation of anti-PEG and anti-pDLS antibody responses

To investigate the anti-PEG and anti-pDLS immune responses, BALB/c mice (6–8 weeks old, female) were s.c. immunized with LNPs containing 1.0 µg of SARS-CoV-2 mRNA formulated using ALC-0159, pDLS1, or pDLS4 (SARS-CoV-2 mRNA-loaded LNPs were prepared via microfluidic mixing). Injections were administered into the left hind leg at three-week intervals, with two doses given in total. A two-dose schedule was chosen to reflect the clinically approved and widely implemented vaccination regimens for mRNA-LNP-based vaccines^19,63,64^. Serum samples were collected every week post-immunization for ELISA analysis, and body weight changes were monitored every week throughout the experiment. The anti-PEG and anti-pDLS antibody titers were assessed by ELISA. Nunc PolySorp 96-well plates (Thermo Fisher Scientific) were coated with ALC-0159, pDLS1, pDLS2, or pDLS4 (200 nmol ml^−1^) and allowed to dry completely overnight at room temperature. The next day, plates were blocked for 2 h at 37 °C using PBS supplemented with 4% BSA (Sigma). Serum of mice was serially diluted in PBS supplemented with 1% BSA and allowed to incubate for 2 h at room temperature. PBS with 1% BSA and 0.5% Tween 20 was used as the dilution buffer in the anti-pDLS antibody study. Plates were rinsed three times with PBS, and anti-mouse IgM antibody (Bethyl Laboratories, Cat# A90-101P, 1:5000) conjugated to horseradish peroxidase was then added. Following a 1-h incubation, the plates were washed three more times before adding 100 µl of KPL SureBlue Reserve TMB Substrate (SeraCare) to each well for a 15-min color development period. The reaction was terminated by adding 100 μL of KPL TMB Stop Solution (SeraCare), and the absorbance was recorded at 450 nm using a Spark Multimode Microplate Reader (TECAN). Antibody titers were determined by identifying the highest serum dilution at which the optical density (OD) exceeded threefold above background. For samples below the detection threshold, an arbitrary value of 10 (half of the initial dilution) was assigned, as reliable titer interpolation was impossible.

### Cryopreserved storage of pDLS-LNP

FLuc mRNA-loaded pDLS-LNPs were formulated using a pDLS lipid with a DP of 23 (comparable to pDLS2) on the NanoAssemblr Ignite platform. The N/P ratio of 6:1 was used. 20% w/v sucrose-containing TBS solution was prepared by adding 1 g of sucrose to 5 ml of TBS. This sucrose mixture was then added to the LNPs at a 1:1 volume ratio and mixed thoroughly by pipetting, resulting in a final sucrose concentration of 10%. The formulated LNPs were stored at –80 °C for long-term preservation.

### In vivo transfection efficiency evaluation of cryopreserved FLuc mRNA-loaded pDLS-LNP

BALB/c mice (6–8 weeks old, female) were subcutaneously immunized at the tail base with cryopreserved or fresh FLuc mRNA-loaded pDLS-LNP (FLuc mRNA-loaded LNPs were prepared via microfluidic mixing) at a dose of 1.0 μg mRNA per mouse (*n* = 3 mice per group). Mice that were not treated with LNPs served as a negative control (*n* = 3 mice). After 6 h, mice were intraperitoneally injected with 200 µL of 15 mg/mL VivoGlo luciferin (Promega, USA). After 10 min, luminescence signal from mice was detected using the IVIS Spectrum imaging system (PerkinElmer, USA). The total flux values of the whole mouse body were measured using Living Image software (PerkinElmer, USA).

### Statistical analysis

All statistical analyses were conducted using GraphPad Prism (version 10). Statistical analyses were performed using two-tailed one-way ANOVA with Tukey’s correction for comparisons involving multiple groups or unpaired two-tailed Student’s *t*-test with Mann-Whitney test for two groups. Results are presented as mean ± SD. Statistical significance is indicated by the specific *p*-value (*p* < 0.05). p > 0.05 indicates no significant difference.

### Reporting summary

Further information on research design is available in the Nature Portfolio Reporting Summary linked to this article.

## Supplementary information

Supplementary Information

Reporting Summary

Transparent Peer Review file

## Source data

Source Data

## Data Availability

Source data are available for Figs. 2a–i, 2l–n, 3c, 3d, 3i, 3k, 4b, 5e, 6i–n, 7b, 7c, 7e–h, 8a, 8b, 8d–k, 8m–p and Supplementary Figs. 23–45, 50, 52-54, 64, 65, and 67 in the associated source data file. The authors declare that the remaining data are available within the Manuscript, Supplementary Information, or Source Data file. Source data are provided with this paper.
