# Supplementary material for: Polypeptide-engineered lipid nanoparticles for mRNA delivery with limited immunogenicity
Source: Nat Commun. 2026 May 29;17:6986. doi: 10.1038/s41467-026-73698-6 (PMC13392015; doi:10.1038/s41467-026-73698-6)
Supplement: Supplementary file 1 — Supplementary Information [file 41467_2026_73698_MOESM1_ESM.pdf]

Supplementary Information

1. Materials and methods

1.1 Materials

Chemical reagents for the synthesis of poly(D, L-serine) (pDLS) lipids were purchased from Sigma-Aldrich and used as received unless otherwise noted. Helper lipid 1,2-distearoyl-sn-glycerol-3-phosphocholine (DSPC), cholesterol and ionizable lipid ALC-0315 were purchased from MedChemExpress (Monmouth Junction, NJ, USA). Sodium acetate was purchased from Sigma-Aldrich (St. Louis, MO, USA). Triton®-X100, Tris-EDTA, and VivoGlo Luciferin (In Vivo Grade) were purchased from Promega (Madison, WI, USA). Alamar Blue and Pierce Firefly Luciferase Glow assay kit were purchased from Invitrogen (Waltham, MA, USA). Other reagents used were of analytical grade.

1.2 Synthesis of o-benzyl-L-serine-N-carboxyanhydride (L-Ser-NCA) and o-benzyl-D-serine-N-carboxyanhydride (D-Ser-NCA)

Synthesis strategy for L-Ser-NCA and D-Ser-NCA was showed in Supplementary Fig. 1. General synthetic method for L-Ser-NCA: O-benzyl-L-serine (6.0 g, 30.8 mmol) was suspended in 100 mL of dry tetrahydrofuran (THF) and then triphosgene (3.4 g) was added under N_2. The mixture was stirred at 70 ℃ under a flow of N_2 for 3 h. After the reaction mixture was cooled down to room temperature, the crude product was precipitated by pouring the mixture solution into hexane (800 mL), collected by filtration.

The resulting crude product was purified by recrystallizing with THF/hexane mixture for three times. The yield of L-Ser-NCA was 67%. The structure of L-Ser-NCA was verified by ^{1}H NMR and ^{13}C NMR spectra (Supplementary Fig. 2 and 3).

General synthetic method for D-Ser-NCA: o-benzyl-D-serine (6.0 g, 30.8 mmol) was suspended in 100 mL of dry tetrahydrofuran (THF) and then triphosgene (3.4 g) was added under N_2. The mixture was stirred at 70 ℃ under a flow of N_2 for 3 h. After the reaction mixture was cooled down to room temperature, the crude product was precipitated by pouring the mixture solution into hexane (800 mL), collected by filtration. The resulting crude product was purified by recrystallizing with THF/hexane mixture for three times. The yield of D-Ser-NCA was 62%. The structure of D-Ser-NCA was verified by ^{1}H NMR and ^{13}C NMR spectra (Supplementary Fig. 4 and 5).

1.3 Synthesis of pDLS1-5 lipids

Synthesis strategy for pDLS lipids with different degree of polymerization (DP) is showed in Supplementary Fig. 6. The initiator, 2-amino-N, N-ditetradecylacetamide, was first synthesized. General synthetic method for 2-amino-N, N-ditetradecylacetamide: Boc-glycine (350.3 mg, 2.0 mmol) and ditetradecylamine (819.6 mg, 2.0 mmol) were dissolved in dry dichloromethane (DCM) (40 mL). 2-(1H-Benzotriazole-1-yl)-1,1,3,3-tetramethyluronium hexafluorophosphate (HBTU) (910.5 mg, 2.4 mmol) and N-ethyl-N-(1-methylethyl)-2-propanamine (DIPEA) (646.1 mg, 5.0 mmol) were added to the solution. The reaction mixture was stirred for 24 h at room temperature under N_2. The mixture was diluted with 50 mL of DCM, and washed with 7% citric acid, brine and H_2O. The resulting organic layer was collected and dried over anhydrous $MgSO_4$. DCM was evaporated in vacuo to yield crude product. The resulting crude product was purified by flash silica gel column chromatography (Hexane/Diethylether 8:2, v/v), obtaining the final product as a yellow oil. The yield of

the compound was 85%. 2-Amino-N, N-ditetradecylacetamide was then synthesized. *tert*-Butyl (2-(ditetradecylamino)-2-oxoethyl)carbamate was dissolved in 5 mL of anhydrous DCM, to which was added 4 mL of trifluoroacetic acid (TFA). The mixture was stirred for 2 h under N_2 atmosphere. The solvents were evaporated in vacuo to yield crude product. The resulting crude product was dissolved in 20 mL of DCM, and then 20 mL of $NaHCO_3$ aqueous solution (10%) was added. The mixture was stirred for 12 h under N_2 atmosphere. The organic layer was collected and dried over anhydrous $MgSO_4$. DCM was evaporated in vacuo. The resulting product was dried under vacuum to obtain a white powder. The yield of the compound was 95%. The structure of product was verified by ^{1}H NMR spectrum (Supplementary Fig. 7).

General synthetic method for poly(o-benzyl-D, L-Ser) lipids: In the glove box, 2-amino-N, N-ditetradecylacetamide (46.6 mg, 0.1 mmol), L-Ser-NCA (331.5 mg, 1.5 mmol) and D-Ser-NCA (331.5 mg, 1.5 mmol) were dissolved in 40 mL of anhydrous DCM. The mixture was stirred for 48 h at room temperature in the glove box. Then, 3.0 mL of acetic anhydride was added and the reaction was continued for 2 h. The crude product was precipitated by pouring the mixture solution into glacial ether (300 mL), collected by centrifugation. The resulting crude product was purified by dissolving with DCM and precipitated by pouring the solution into glacial ether. The resulting product was dried under vacuum. The number of polymerization unit for poly(o-benzyl-D, L-Ser) lipid (protected pDLS) can be adjusted by varying the amount of L-Ser-NCA and D-Ser-NCA. The reactant feed ratios for the synthesis of poly(o-benzyl-D, L-Ser) lipids are listed in Supplementary Table 1. The yields of the protected polypeptide lipids for pDLS1, pDLS2, pDLS3, pDLS4 and pDLS5, were 58%, 63%, 70%, 72% and 78%, respectively. The typical structure of poly(o-benzyl-D, L-Ser) lipid was verified by ^{1}H NMR (Supplementary Fig. 8-12).

The deprotection of poly(o-benzyl-D, L-Ser) lipids was then performed to yield

pDLS lipids. General synthetic method for pDLS lipid: 1.0 g of lipid-poly(o-benzyl-D, L-Ser) was dissolved in 10 mL of TFA, to which was added 3 mL of 33% HBr in acetic acid. The mixture was stirred in ice bath for 2 h. The solvents were removed in vacuo. The resulting crude product was suspended in 30 mL of methanol and then precipitated by pouring the mixture solution into glacial ether (300 mL), collected by centrifugation. The crude product was purified by suspending it in methanol and precipitated by pouring the solution into glacial ether. The obtained product was dried under vacuum. The crude pDLS lipid was purified by dialysis with deionized water. The final pDLS lipid was obtained by freeze-drying under vacuum. The yields of the deprotected polypeptide lipids for pDLS1, pDLS3, pDLS4 and pDLS5, were 55%, 58%, 64%, 75% and 87%, respectively. The typical structure of pDLS1-5 lipids was verified by ^{1}H NMR (Supplementary Fig. 13-17).

1.4 Synthesis of pDLS18D, pDLS18S, pDLS14S and pDLS8D lipids

PDLS lipids with different lipid chain were synthesized by using various lipids with amine group as initiator. By using 2-amino-N,N-dioctadecylacetamide, octadecan-1-amine, tetradecan-1-amine and 6-amino-N,N-dioctylhexanamide as the initiators, pDLS18D, pDLS18S, pDLS14S and pDLS8D lipids were synthesized, respectively. General synthetic method for pDLS18D, pDLS18S, pDLS14S and pDLS8D lipids: In the glove box, the initiator (46.6 mg, 0.1 mmol), L-Ser-NCA (552.5 mg, 2.5 mmol) and D-Ser-NCA (552.5 mg, 2.5 mmol) were dissolved in 40 mL of anhydrous DCM. The mixture was stirred for 48 h at room temperature in the glove box. Then, 3.0 mL of acetic anhydride was added and the reaction was continued for 2 h. The crude product was precipitated by pouring the mixture solution into glacial ether (300 mL), collected by centrifugation. The resulting crude product was purified by dissolving with DCM and precipitated by pouring the solution into glacial ether. The resulting product was dried under vacuum.

The deprotection of poly(o-benzyl-D, L-Ser) lipids was then performed to yield pDLS lipids. General synthetic method for pDLS lipid: 1.0 g of poly(o-benzyl-D, L-Ser) lipid was dissolved in 10 mL of TFA, to which was added 3 mL of 33% HBr in acetic acid. The mixture was stirred in ice bath for 2 h. The solvents were removed in vacuo. The resulting crude product was suspended in 30 mL of methanol and then precipitated by pouring the mixture solution into glacial ether (300 mL), collected by centrifugation. The crude product was purified by suspending it in methanol and precipitated by pouring the solution into glacial ether. The obtained product was dried under vacuum. The crude pDLS lipid was purified by dialysis with deionized water. The final pDLS lipid was obtained by freeze-drying under vacuum. The yields of the deprotected polypeptide lipids for pDLS18D, pDLS18S, pDLS14S and pDLS8D, were 75%, 78%, 74%, and 72%, respectively. The typical structure of pDLS18D, pDLS18S, pDLS14S and pDLS8D lipids was verified by ^{1}H NMR (Supplementary Fig. 18-21).

1.5 Formulation and characterization of mRNA-loaded LNPs for comparisons of different batches of pDLS lipid and formulating methods

ALC-0315, DSPC, cholesterol, and ALC-0159 (MedChem Express) were dissolved in ethanol to stock concentrations of 20.0 mg/mL, 10.0 mg/mL, 10.0 mg/mL, and 3.0 mg/mL, respectively. pDLS lipids were dissolved in nuclease-free water and ethanol in a 1:4 (v/v) ratio to obtain a stock concentration of 3.0 mg/mL. The organic phase was prepared by mixing ALC-0315, DSPC, cholesterol, and ALC-0159 or pDLS lipid at a molar ratio of 46.3:9.4:42.7:1.6. The aqueous phase consisted of firefly luciferase (FLuc) mRNA diluted in 10 mM sodium acetate buffer (pH 4) at a nitrogen to phosphate (N/P) ratio of 6. For manually mixed LNP formulations, the organic phase was rapidly mixed with the aqueous phase at a 1:3 (v/v) ratio, followed by incubation at room temperature for 30 min. For LNPs formulated using microfluidic mixing, the organic and aqueous phases were mixed at a 1:3 (v/v) ratio at a total flow rate of 12 mL/min

using the NanoAssemblr Ignite microfluidics system (Cytiva). The resulting LNPs were diluted 25-fold with 1 × PBS (1st Base) and concentrated using 30 kDa MWCO spin columns (Sartorius) by centrifugation at 800 × g, 4 ºC for 40-min intervals. Following formulation, the size, PDI, and zeta potential of mRNA-LNPs were characterized using a Zetasizer (Malvern). Encapsulation efficiency of mRNA-LNPs was quantified using Quant-iT RiboGreen RNA Reagent and Kit (Invitrogen) according to the manufacturer's instructions.

Note: In this study, pipette mixing and microfluidic mixing approaches were used for mRNA-LNP formulations to balance the needs of different experimental phases. Pipette mixing was used for the initial, functional screening of pDLS-LNP formulation parameters (e.g., pDLS lipid structures, mole ratios of pDLS lipids) as well as in vitro studies. This method allowed for rapid preparation with minimal material consumption, which was critical for efficiently identifying lead pDLS-LNP formulations. Once lead pDLS-LNP formulations were identified, microfluidic mixing was used for all subsequent *in vivo* studies. This method was chosen for its superior control over particle size, low polydispersity, and high batch-to-batch reproducibility, which is essential for translational relevance. The mRNA-LNP preparation approaches used for the presented data is also specified in the Methods section and the figure legends.

1.6 Evaluation of transfection efficiency and cytocompatibility of FLuc mRNA-loaded LNPs prepared from different batches of pDLS lipid and formulating methods

Mouse dendritic cells (DC2.4) and human embryonic kidney cells (HEK 293T) were seeded at a cell density of 1 × 10^4 cells/well in white or black 96-well clear-bottom plates. The seeded plates were incubated at 37 ºC, 5% CO_2, for 24 h. Subsequently, mRNA-LNPs formulated by manual or microfluidic mixing were added at a dose of 100 ng mRNA/well, followed by incubation at 37 ºC with 5% CO_2, for 48 h. For

cytocompatibility assessment, spent media from cells cultured in black 96-well plates were aspirated, and culture media containing 10% AlamarBlue were added to each well. Plates were incubated at 37 ºC with 5% CO_2 for 2 h. Fluorescence was measured using a microplate reader (Tecan Spark 10M) at the excitation and emission wavelengths of 570 nm and 600 nm, respectively. Cell viability was calculated from blank-corrected fluorescence values using the following formula:

$$\text{Cell viability (\%)} = \frac{\text{Sample fluorescense intensity}}{A\text{verage of fluorescence of untreated control}} \times 100$$

For evaluation of translation efficiency, 100 µL of ONE-Glo™ Luciferase assay reagent (Promega) was added to each well in the white 96-well plate and incubated at 37 ºC for 10 min in the dark. Luminescence was subsequently measured using the microplate reader (Tecan Spark 10M).

1.7 Cytocompatibility assessment of Cy5-labelled mRNA-loaded LNPs at high doses

DC2.4 and HEK 293T cells were seeded at a cell density of 1 × 10^5 cells/well in 24-well clear flat-bottom plates and incubated at 37 ºC with 5% CO_2, for 24 h. Cells were then treated with Cy5-labelled mRNA-LNPs at a dose of 1000 ng or 2000 ng mRNA/well (n = 3 independent biological samples) and incubated for an additional 48 h at 37 ºC with 5% CO_2. After incubation, spent media was aspirated and replaced with culture media containing 10% AlamarBlue. Plates were incubated for 2 h at 37 ºC with 5% CO_2, after which fluorescence was measured at the excitation and emission wavelengths of 570 nm and 600 nm, respectively, using the microplate reader (Tecan Spark 10M). Cell viability was calculated as described above.

1.8 Formulation and characterization of siGFP-loaded LNPs

ALC-0315, DSPC, cholesterol, and ALC-0159 (MedChem Express) were dissolved in

ethanol to stock concentrations of 20.0 mg/mL, 10.0 mg/mL, 10.0 mg/mL, and 3.0 mg/mL, respectively. pDLS lipids were dissolved in nuclease-free water and ethanol in a 1:4 (v/v) ratio to obtain a stock concentration of 3.0 mg/mL. The organic phase was prepared by mixing ALC-0315, DSPC, cholesterol, and ALC-0159 or pDLS lipid at a molar ratio of 46.3:9.4:42.7:1.6. GFP-22 siRNA (Qiagen, Cat# 1022064) was denoted as siGFP in this study. The aqueous phase consisted of siGFP diluted in 10 mM sodium acetate buffer (pH 4) at a nitrogen to phosphate (N/P) ratio of 6. siGFP-loaded LNPs were formulated *via* manual mixing. The organic phase was rapidly mixed with the aqueous phase at a 1:3 (v/v) ratio, followed by incubation at room temperature for 30 min. Following formulation, the size, PDI, and zeta potential of siGFP-loaded LNPs were characterized using a Zetasizer (Malvern). Encapsulation efficiency of mRNA-LNPs was quantified using Quant-iT RiboGreen RNA Reagent and Kit (Invitrogen) according to the manufacturer's instructions.

1.9 Evaluation of knockdown efficiency of siGFP-loaded LNPs

GFP-expressing HEK293 (HEK293-GFP) cells were seeded at a cell density of 1×10^5 cells/well in 24-well clear flat-bottom plates and incubated at 37 ºC with 5% CO_2, for 24 h. Cells were then treated with siGFP-loaded LNPs (siGFP-loaded LNPs were prepared *via* pipette mixing) at a dose of 1000 ng siGFP/well (n = 3 independent biological samples) and incubated for an additional 48 h at 37 ºC with 5% CO_2. Cells were collected and subsequently analyzed on a FACSymphony A3 flow cytometer (BD Biosciences). The resulting data were processed and visualized using the FlowJo software.

1.10 Cytocompatibility assessment of siGFP-loaded LNPs

GFP-expressing HEK293 cells were seeded at a cell density of 1×10^4 cells/well in black 96-well clear-bottom plates. The seeded plates were incubated at 37 ºC, 5% CO_2, for 24 h. Subsequently, siGFP-loaded LNPs formulated by manual mixing were added

at a dose of 100 ng siGFP/well, followed by incubation at 37 ºC with 5% CO_2 for 48 h. Then, spent media from cells cultured in black 96-well plates were aspirated, and culture media containing 10% AlamarBlue were added to each well. Plates were incubated at 37 ºC with 5% CO_2 for 2 h. Fluorescence was measured using the microplate reader (Tecan Spark 10M) at the excitation and emission wavelengths of 570 nm and 600 nm, respectively. Cell viability was calculated as described above.

2. Supplementary figures

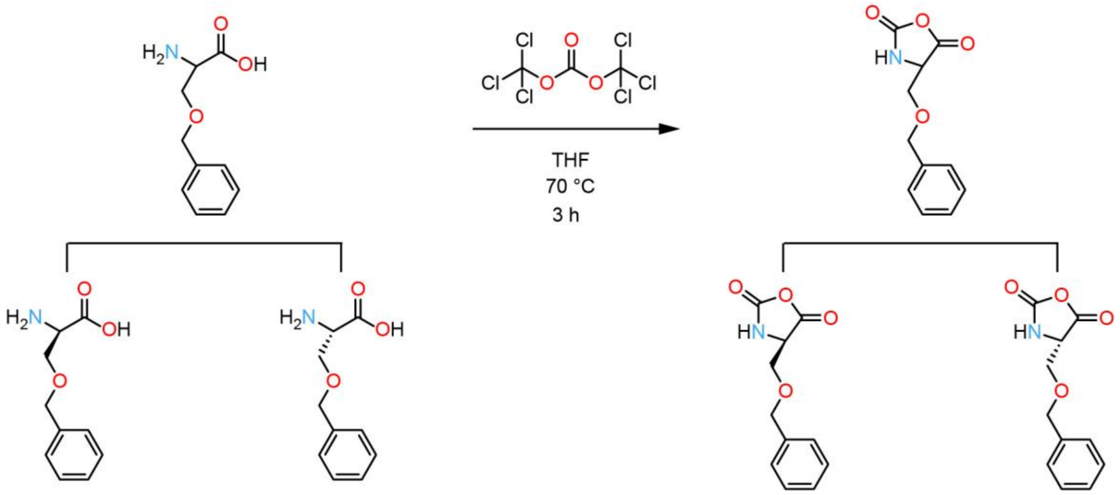

Supplementary Fig. 1 Synthesis of serine-N-carboxyanhydride.

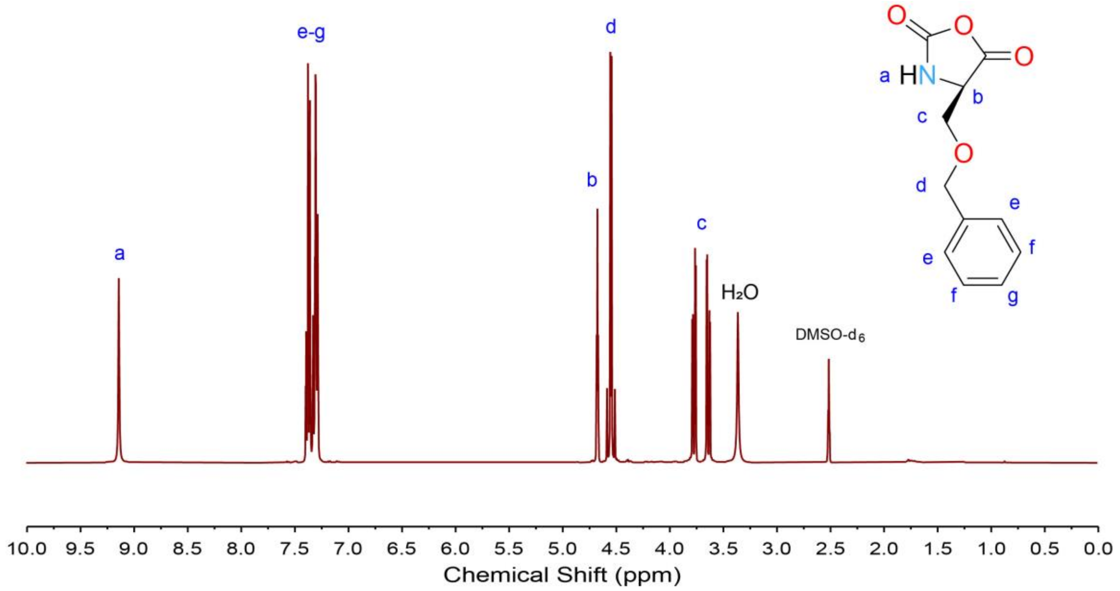

Supplementary Fig. 2 ^{1}H NMR spectrum of of L-serine NCA (solvent: DMSO-d_6).

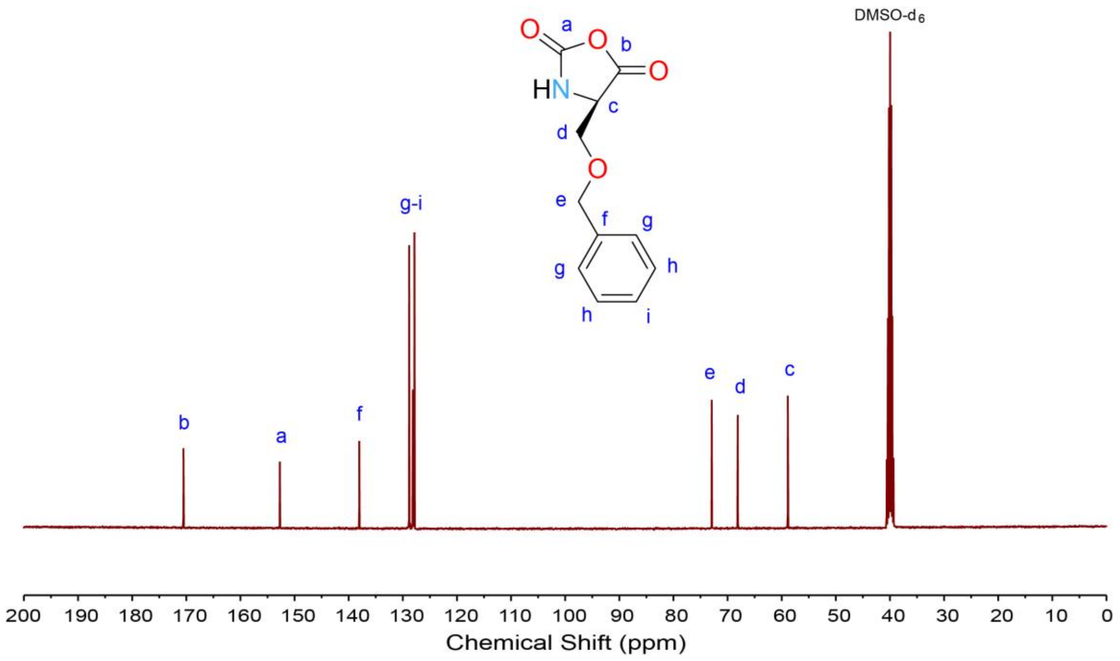

Supplementary Fig. 3 ^{13}C NMR spectrum of L-serine NCA (solvent: DMSO-d_6).

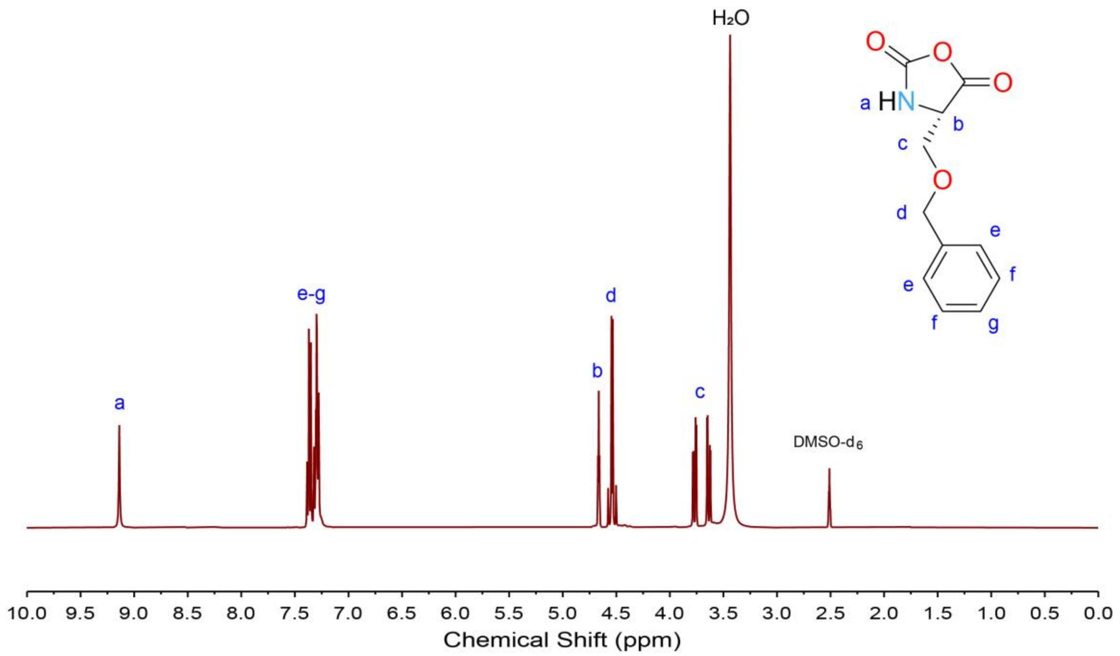

Supplementary Fig. 4 ^{1}H NMR spectrum of D-serine NCA (solvent: DMSO-d_6).

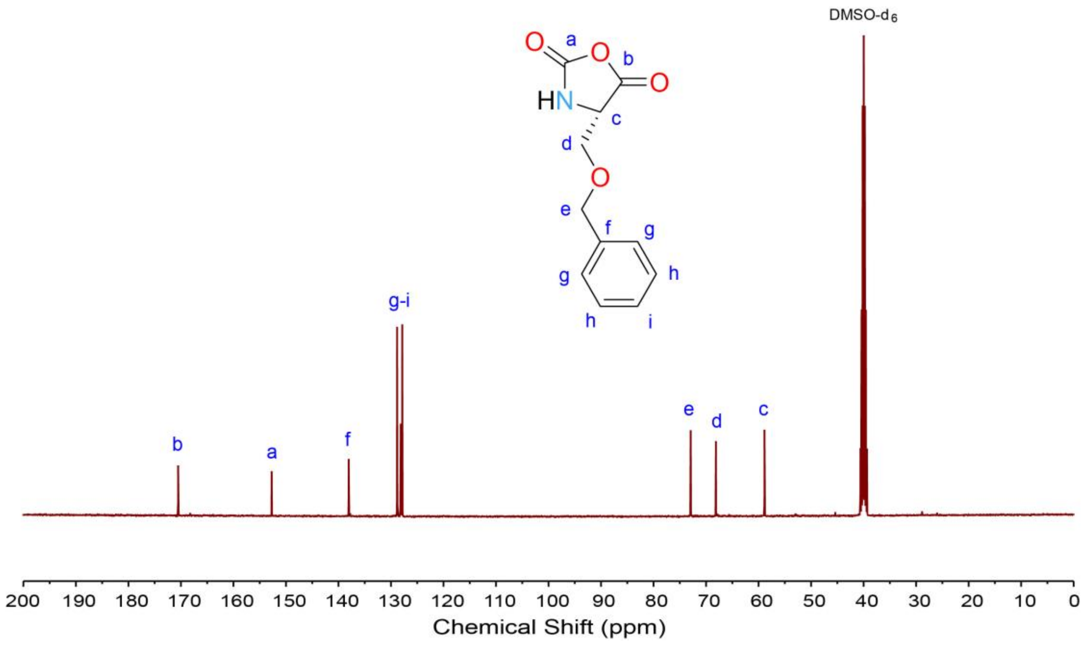

Supplementary Fig. 5 ^{13}C NMR spectrum of D-serine NCA (solvent: DMSO-d_6).

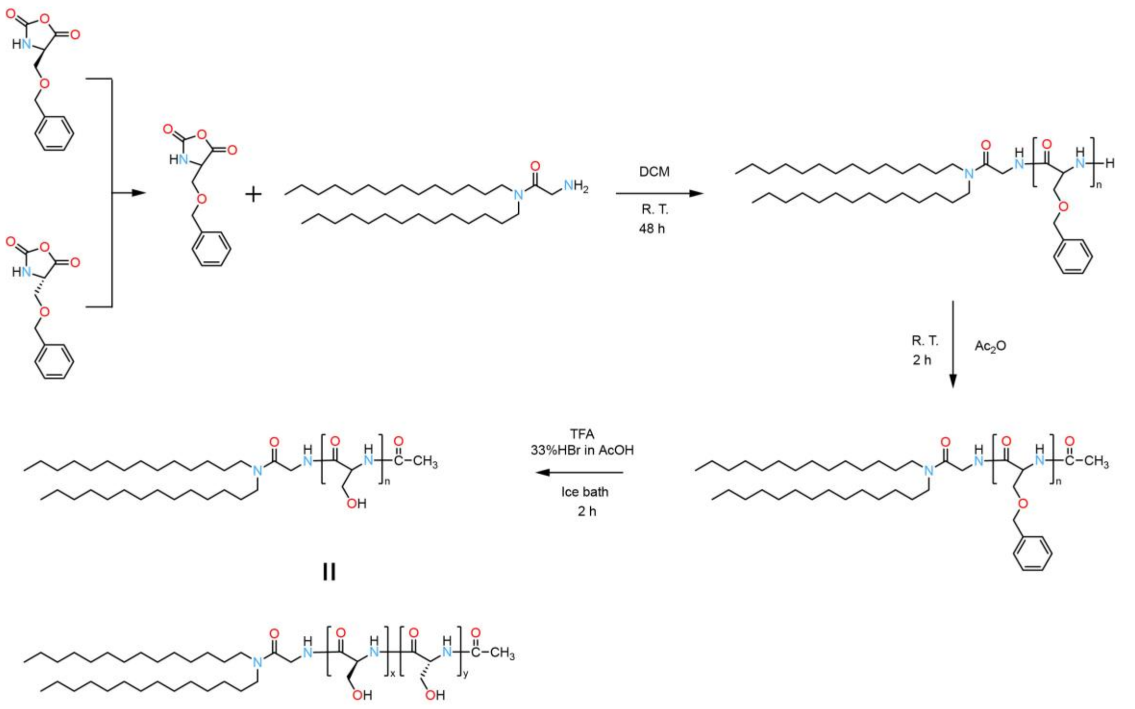

Supplementary Fig. 6 Synthesis of poly(D, L-Serine) (pDLS) lipids.

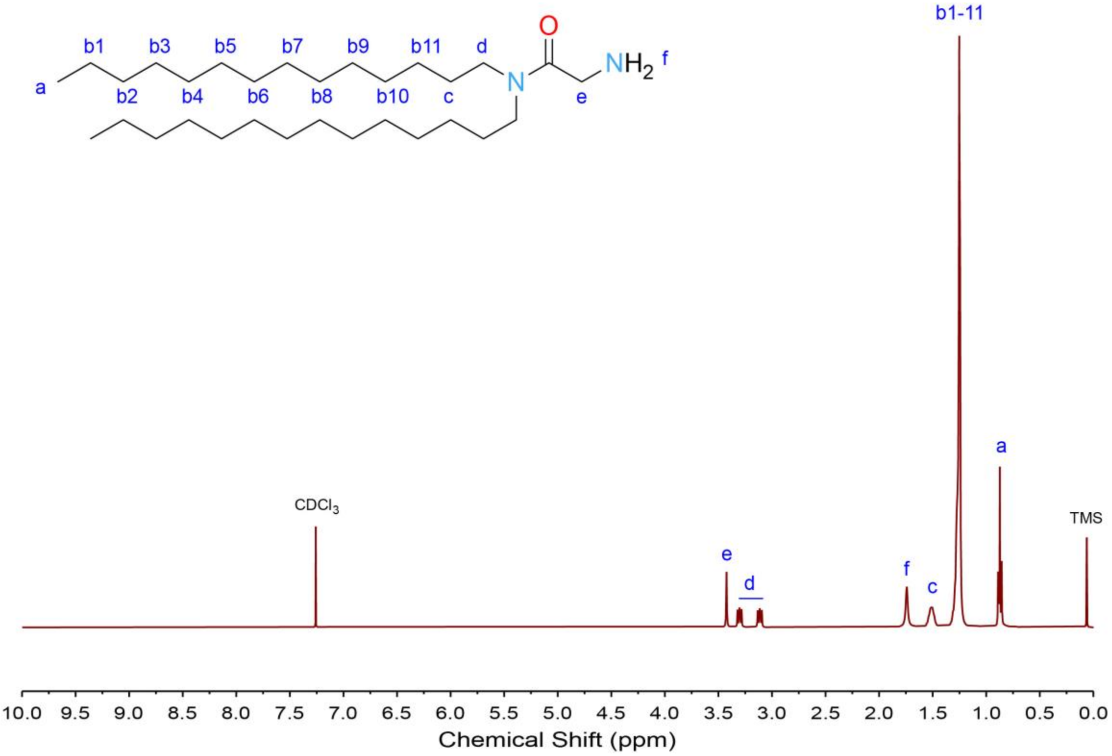

Supplementary Fig. 7 ^{1}H NMR spectrum of 2-amino-N, N-ditetradecylacetamide (solvent: $CDCl_3$).

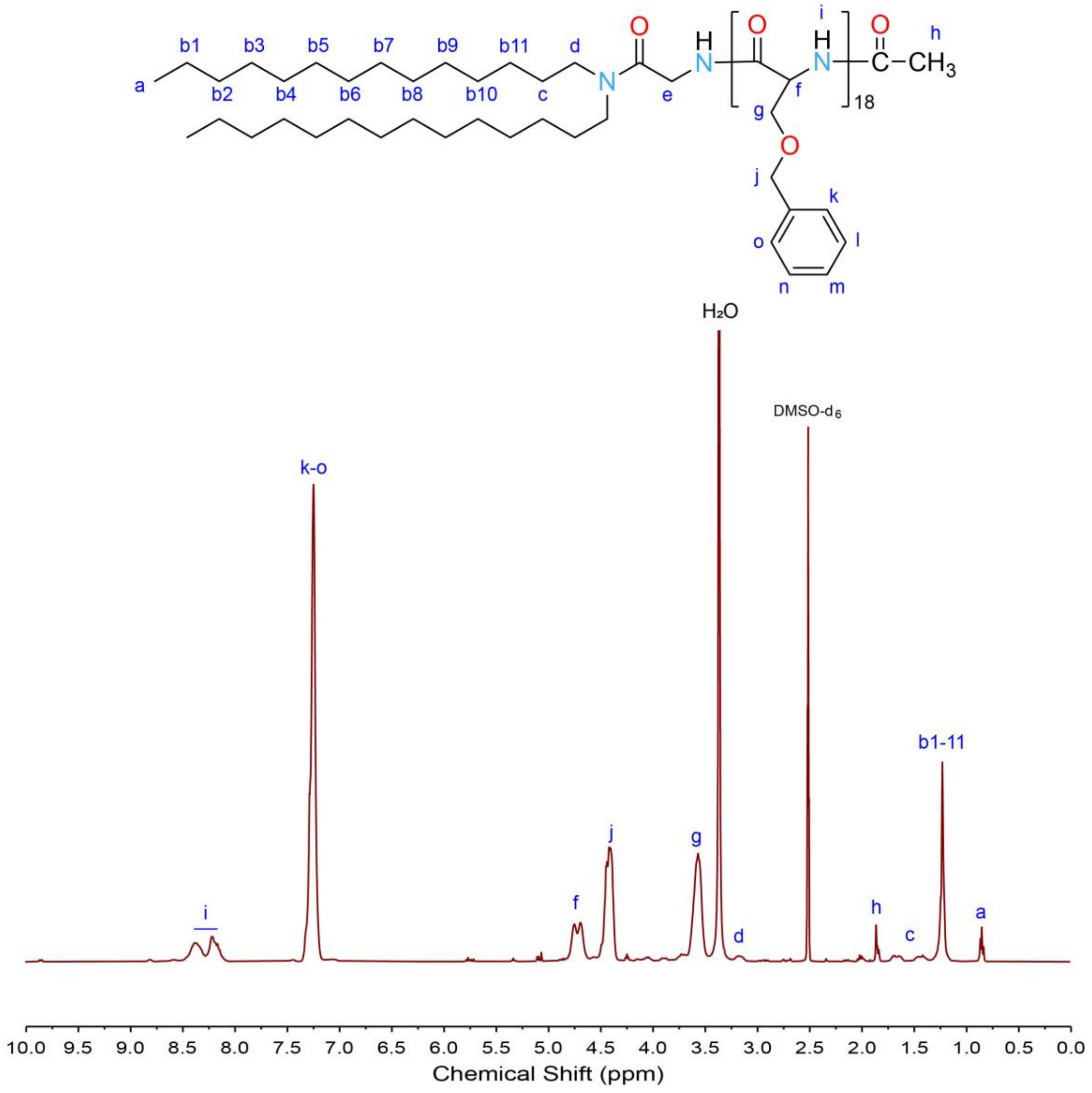

Supplementary Fig. 8 ^{1}H NMR spectrum of poly(*o*-benzyl-D, L-Serine)$_{18}$ lipid (protected pDLS1, solvent: DMSO-d_6).

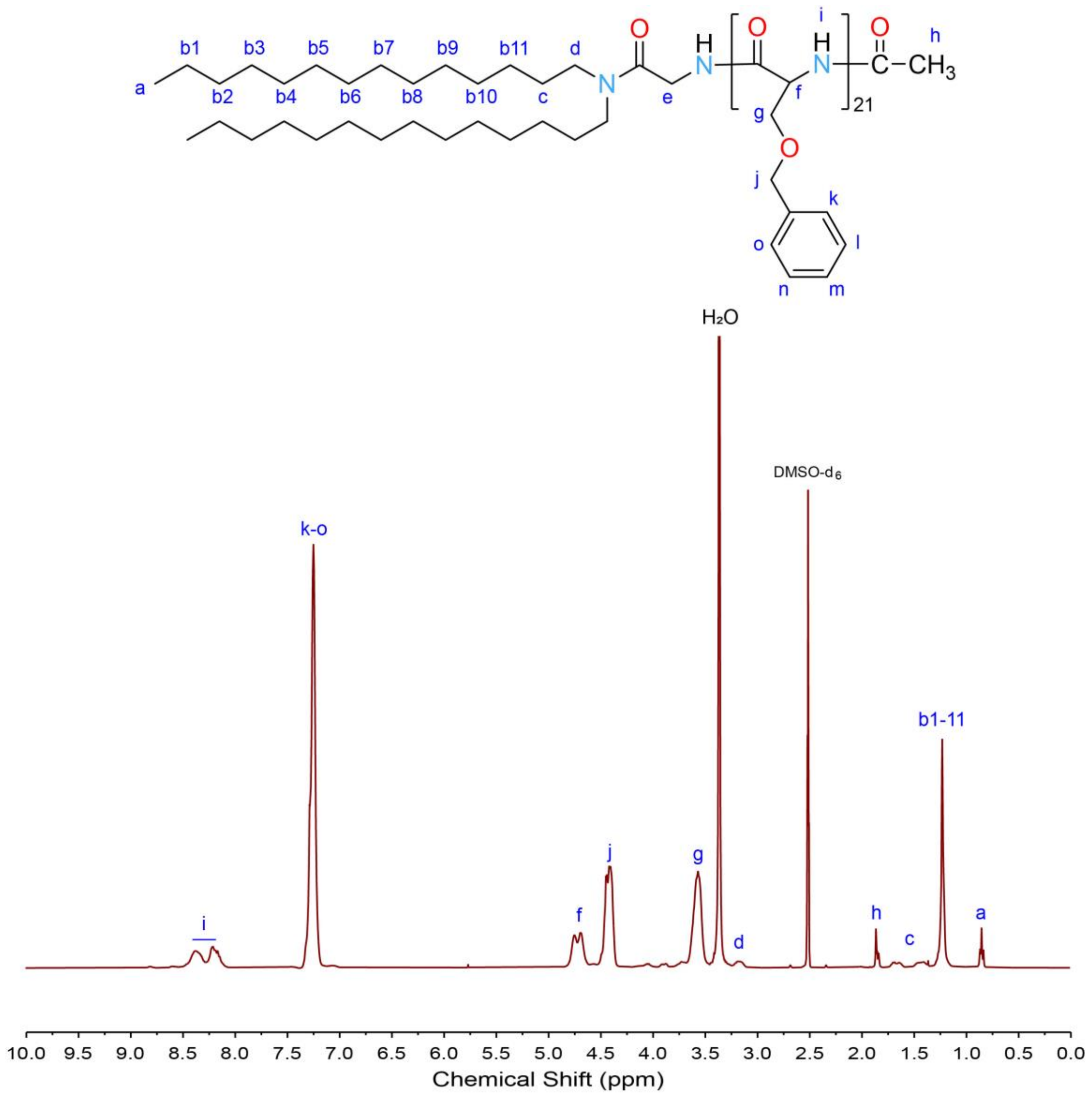

Supplementary Fig. 9 ^{1}H NMR spectrum of poly(*o*-benzyl-D, L-Serine)$_{21}$ lipid (protected pDLS2, solvent: DMSO-d_6).

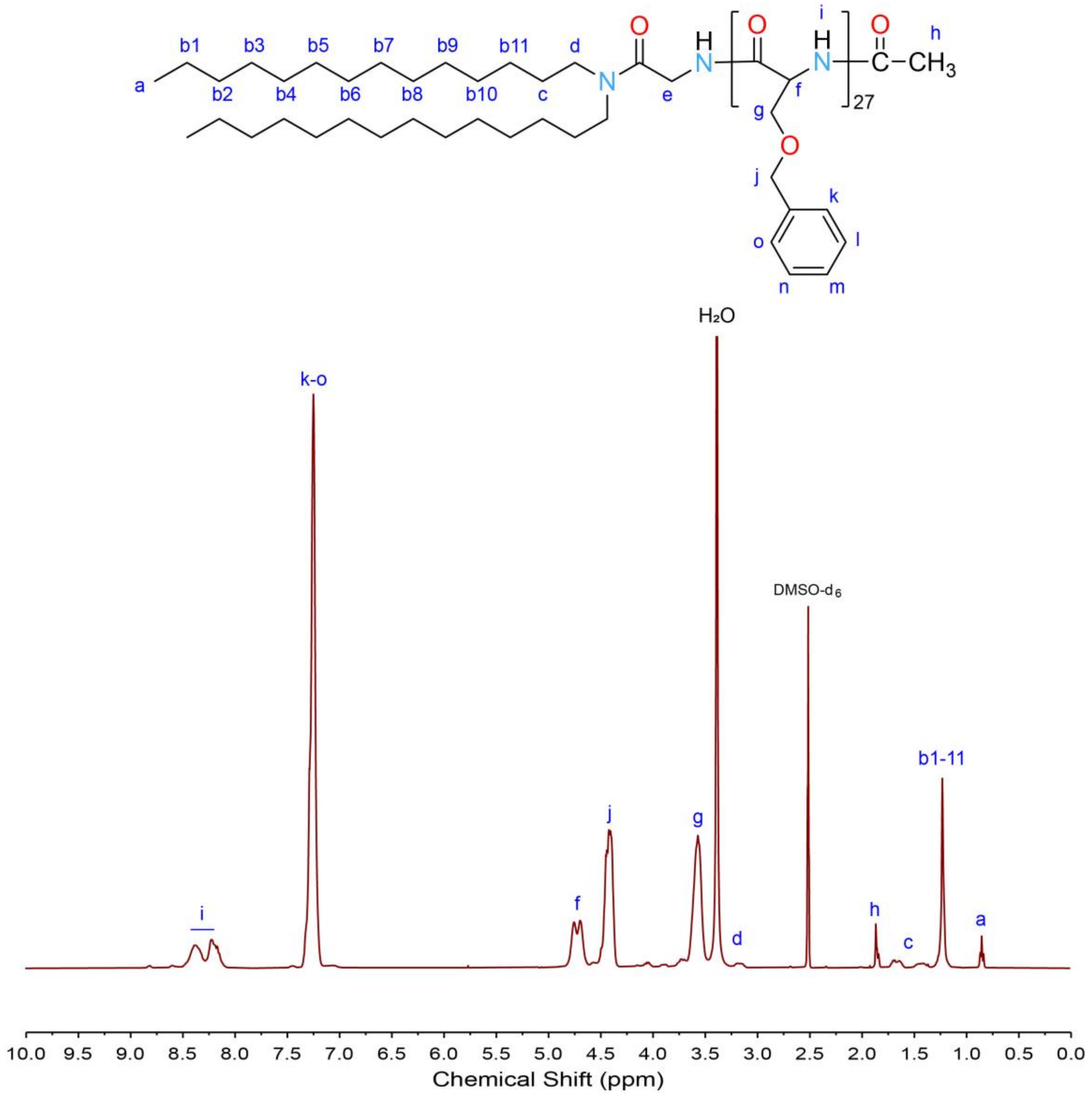

Supplementary Fig. 10 ^{1}H NMR spectrum of poly(*o*-benzyl-D, L-Serine)$_{27}$ lipid (protected pDLS3, solvent: DMSO-d_6).

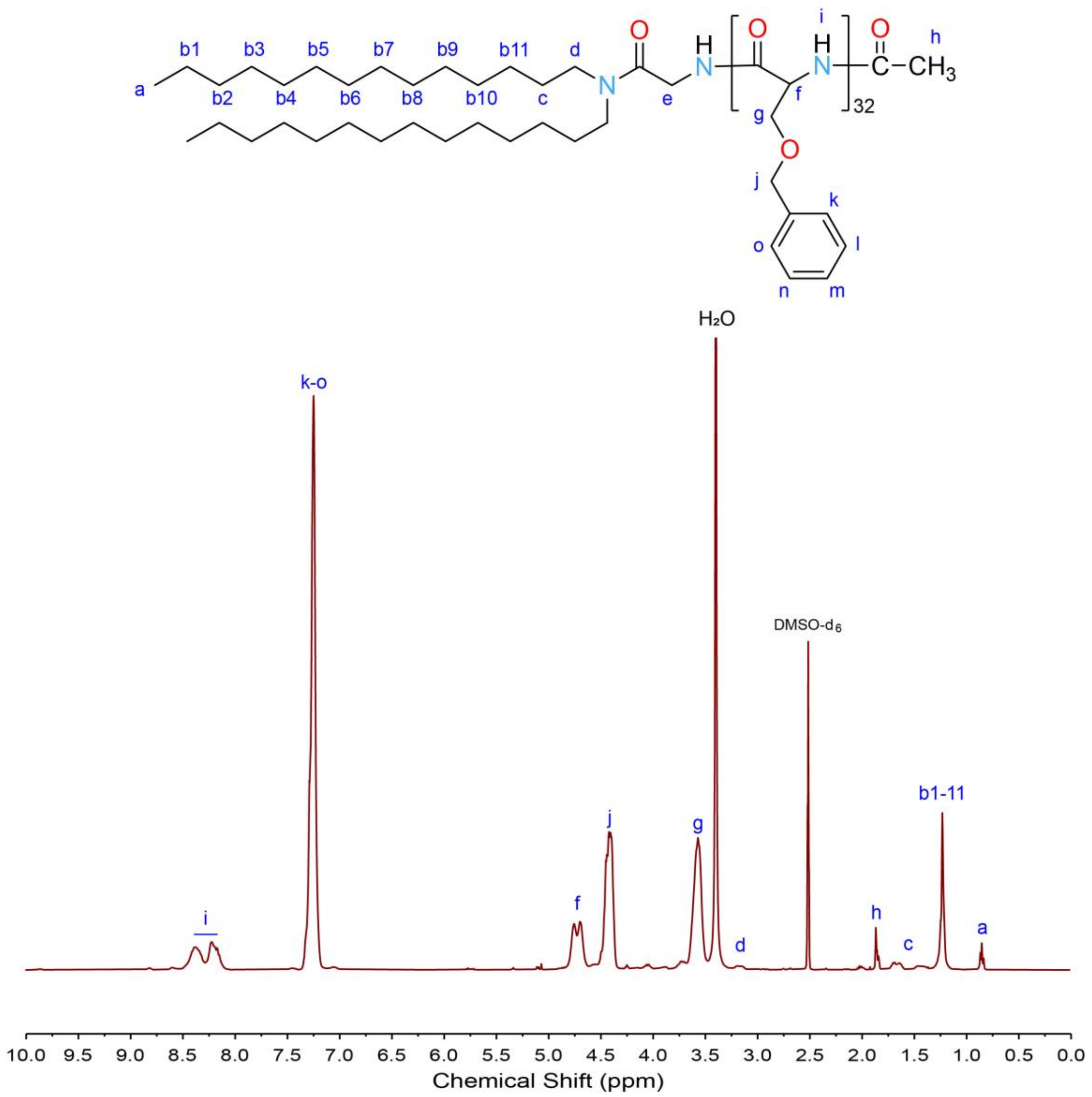

Supplementary Fig. 11 ^{1}H NMR spectrum of poly(*o*-benzyl-D, L-Serine)$_{32}$ lipid (protected pDLS4, solvent: DMSO-d_6).

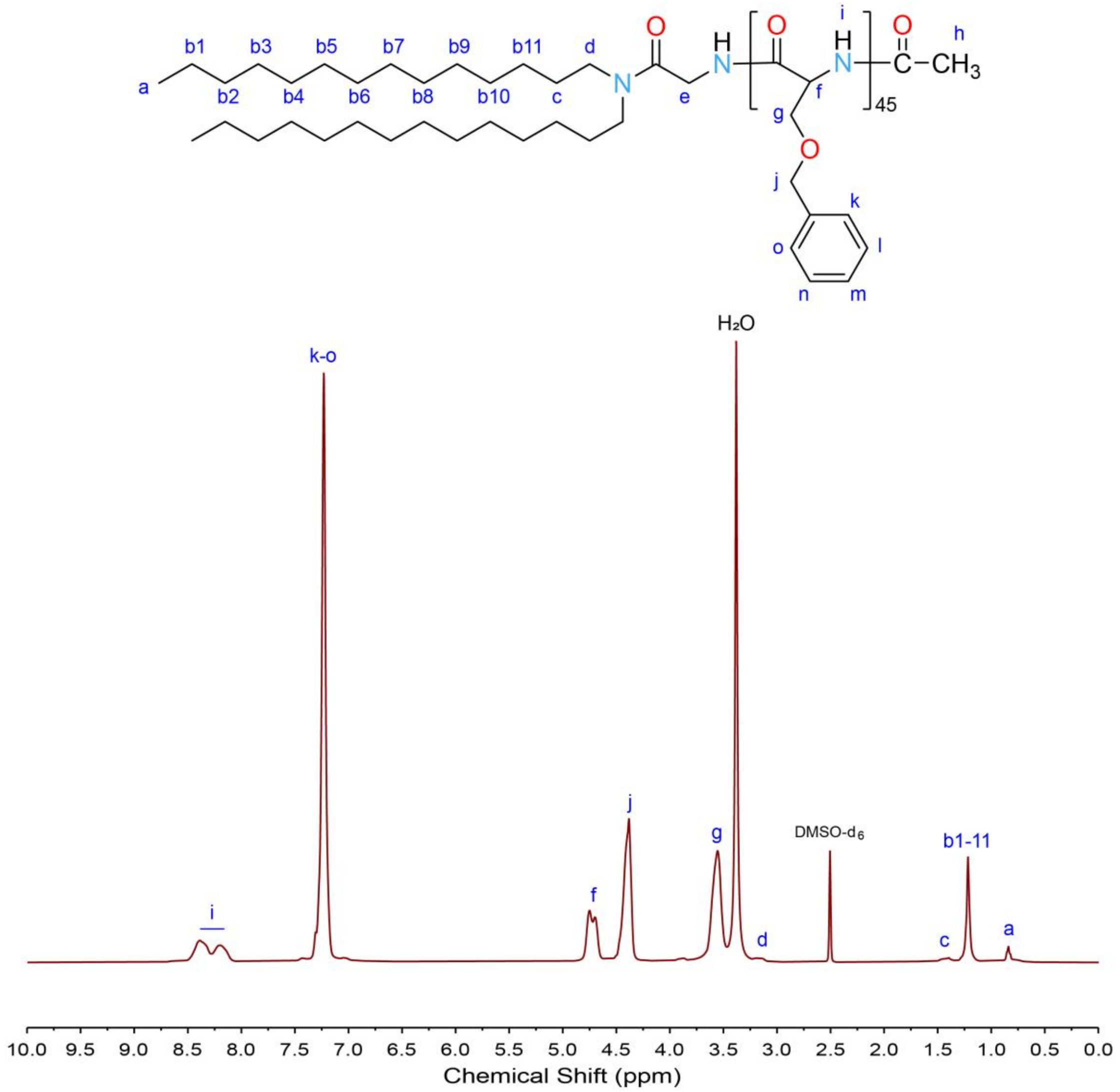

Supplementary Fig. 12 ^{1}H NMR spectrum of poly(*o*-benzyl-D, L-Serine)$_{45}$ lipid (protected pDLS5, solvent: DMSO-d_6).

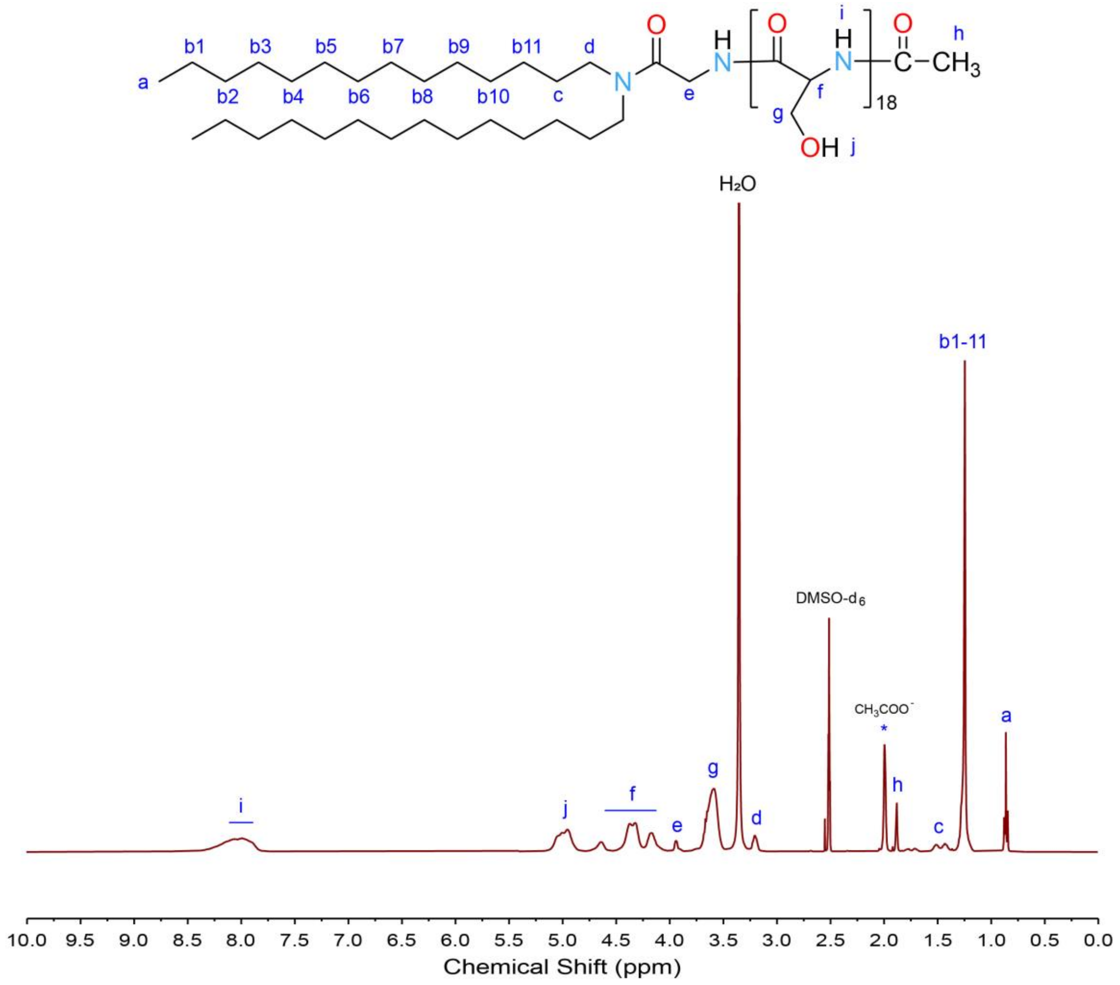

Supplementary Fig. 13 ^1H NMR spectrum of poly(D, L-Serine)$_{18}$ lipid (pDLS1, solvent: DMSO-d_6).

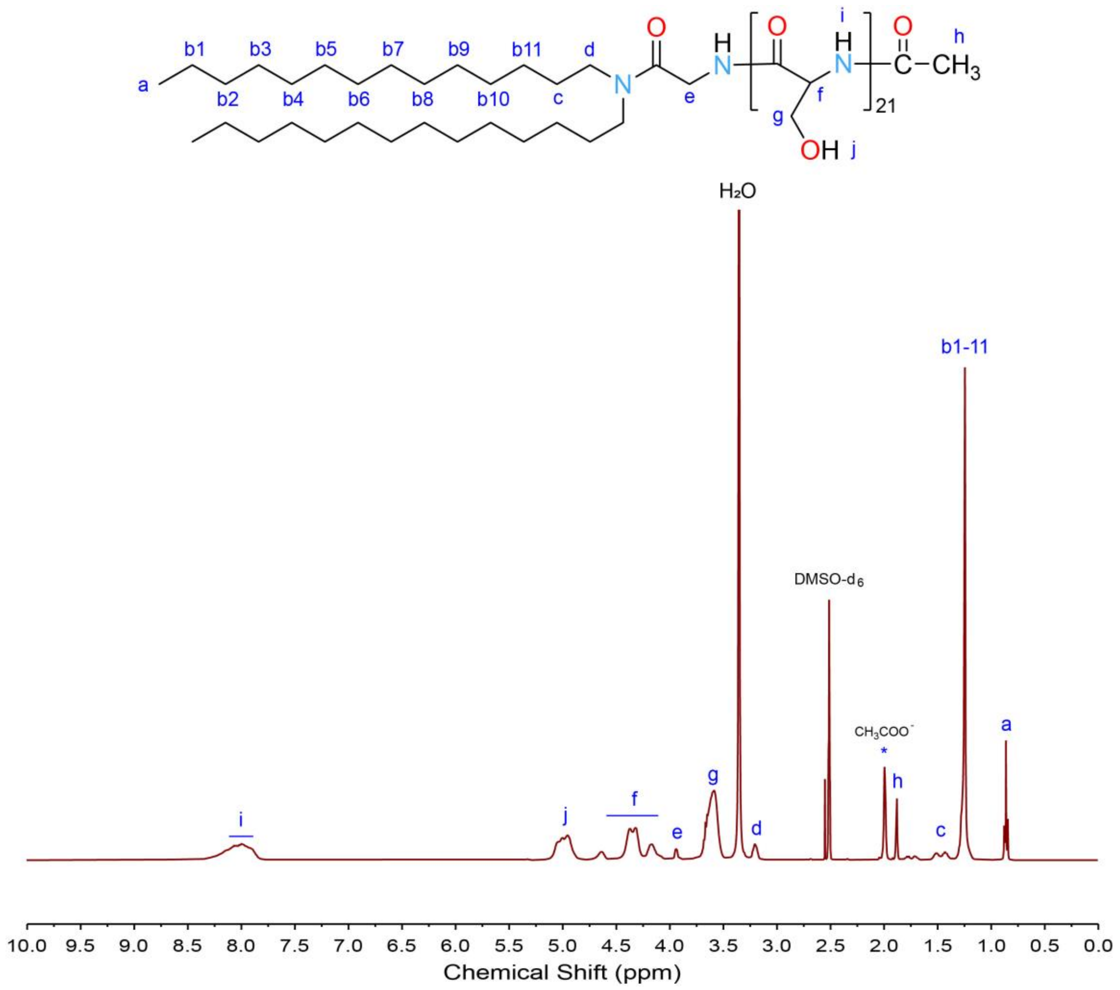

Supplementary Fig. 14 ^{1}H NMR spectrum of poly(D, L-Serine)$_{21}$ lipid (pDLS2, solvent: DMSO-d_6).

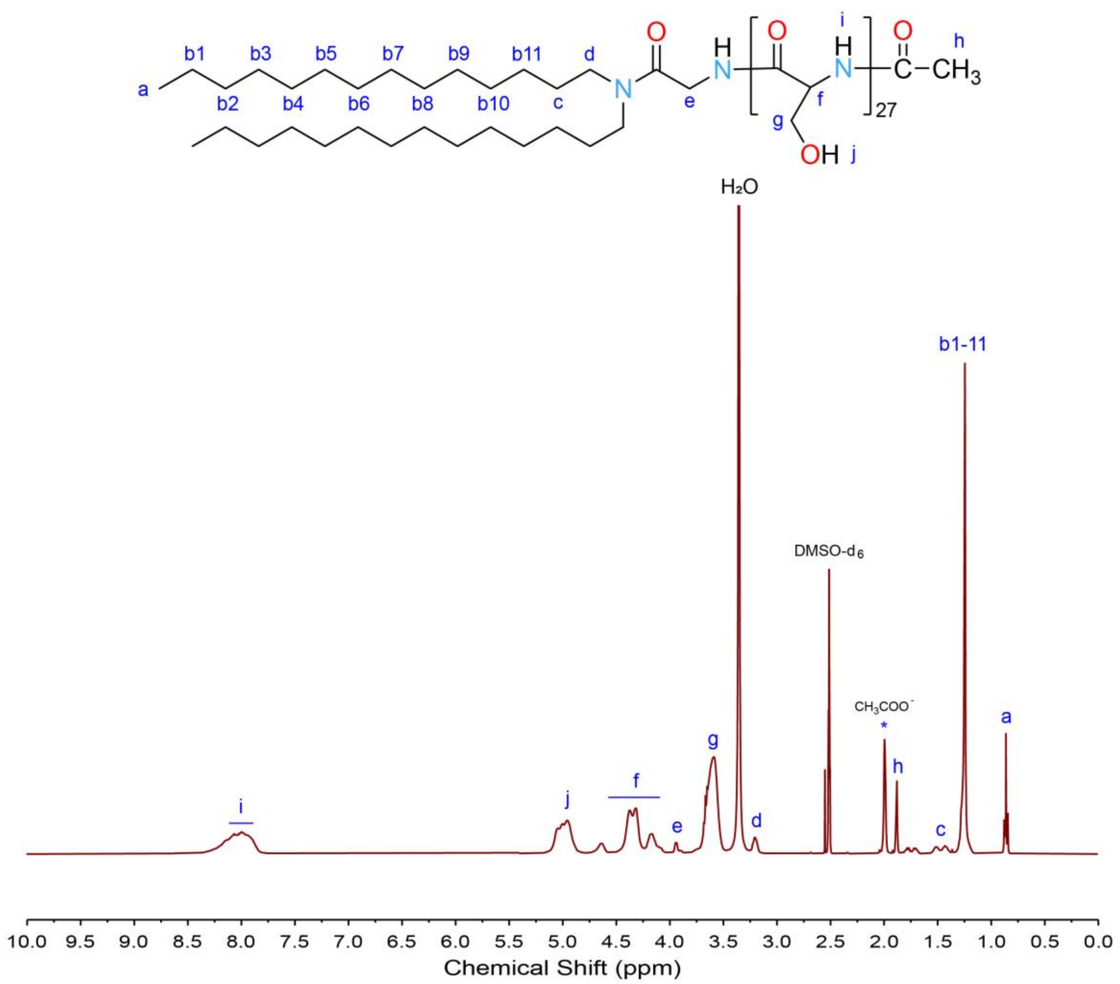

Supplementary Fig. 15 ^{1}H NMR spectrum of poly(D, L-Serine)$_{27}$ lipid (pDLS3, solvent: DMSO-d_6).

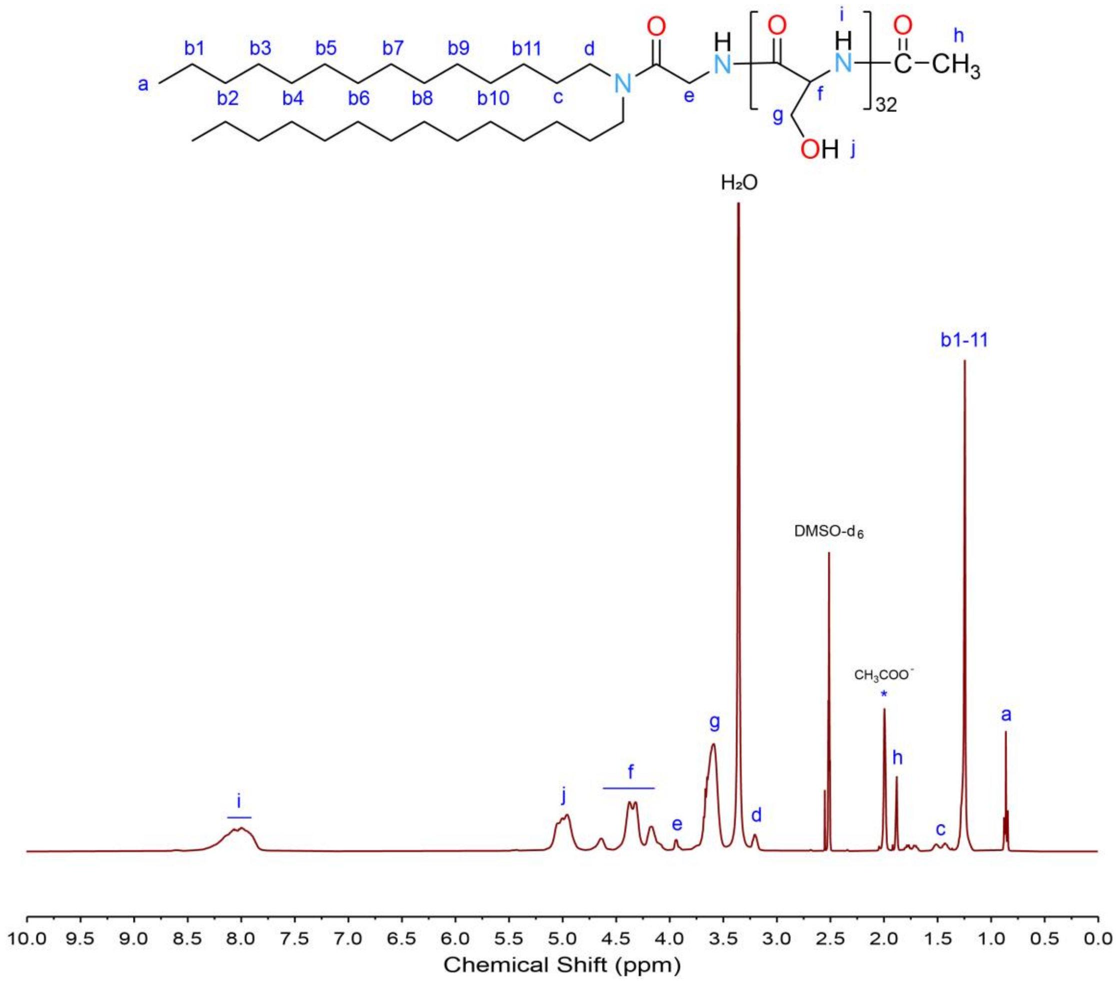

Supplementary Fig. 16 ^{1}H NMR spectrum of poly(D, L-Serine)$_{32}$ lipid (pDLS4, solvent: DMSO-d_6).

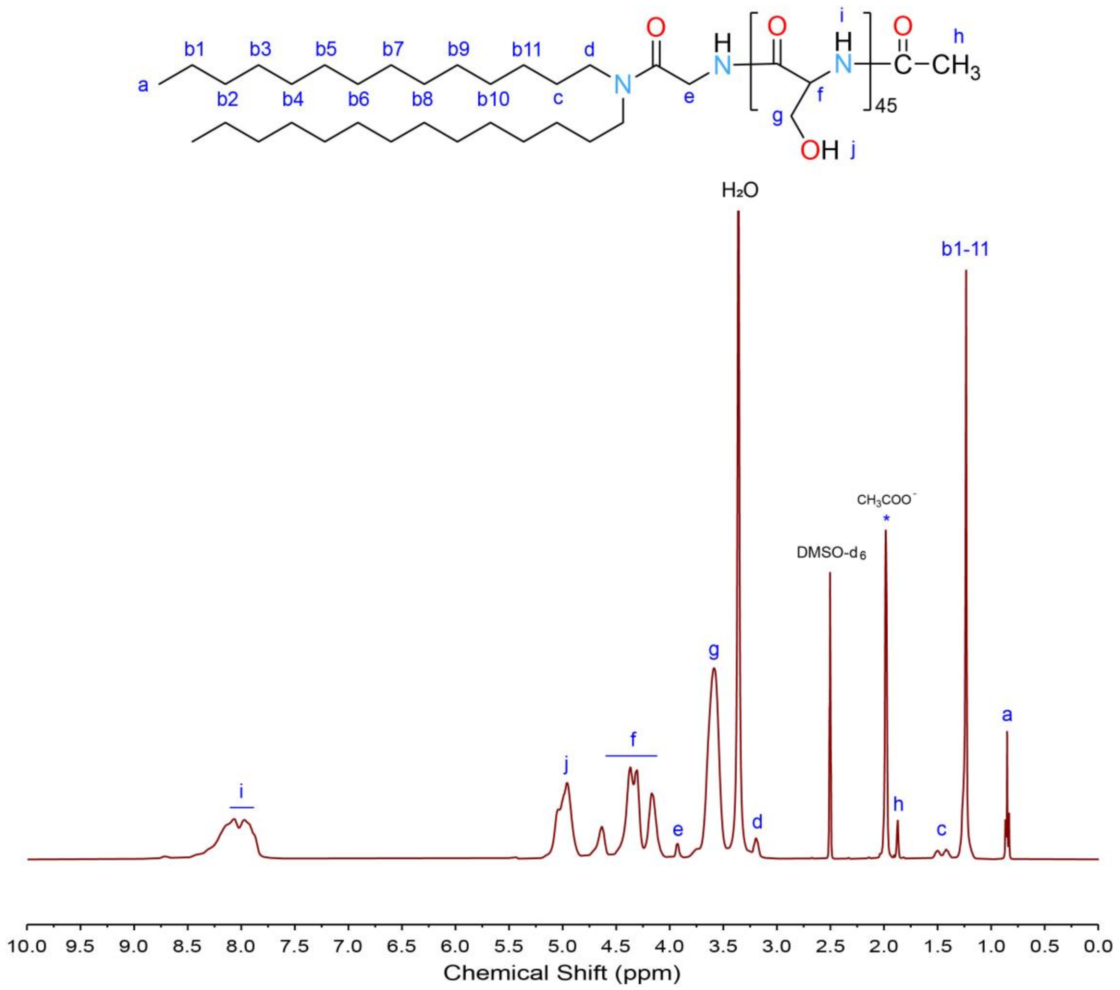

Supplementary Fig. 17 ^{1}H NMR spectrum of poly(D, L-Serine)$_{45}$ lipid (pDLS5, solvent: DMSO-d_6).

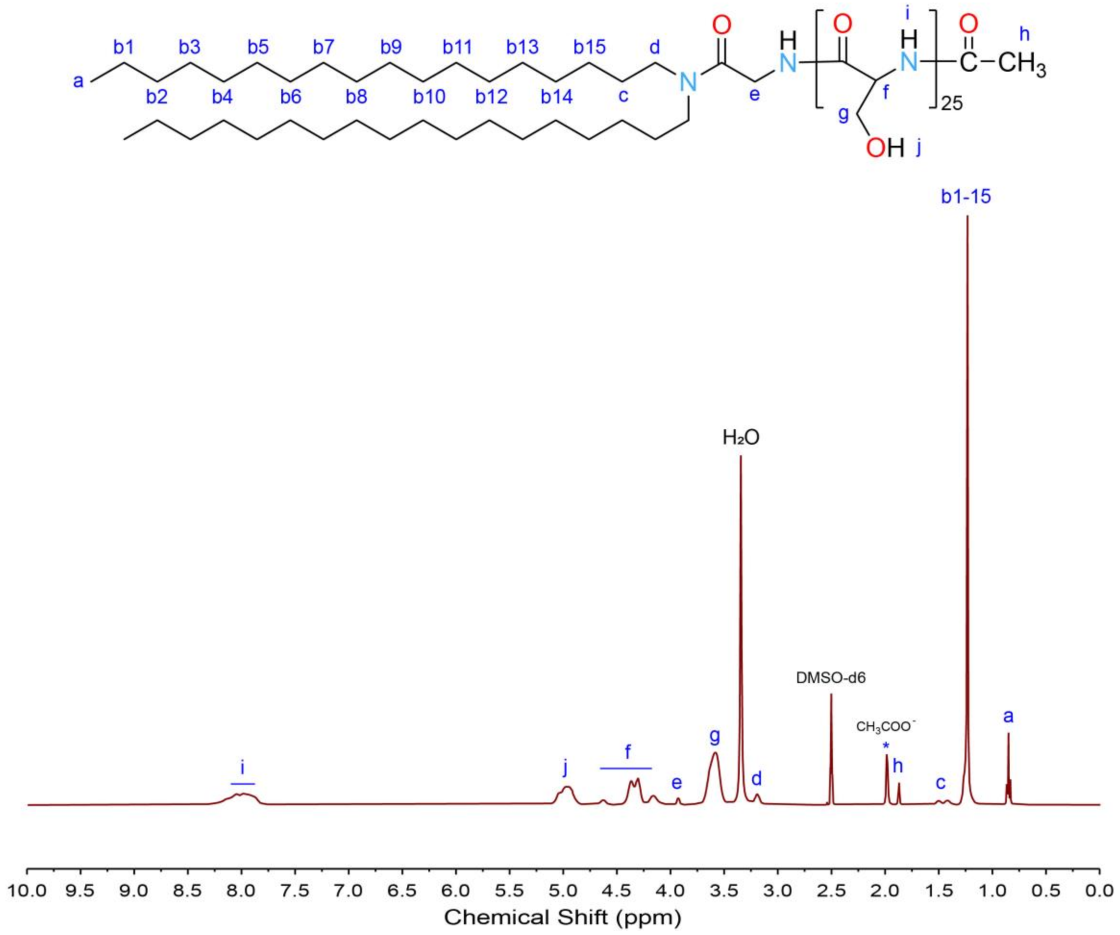

Supplementary Fig. 18 ^{1}H NMR spectrum of C18D-poly(D, L-Serine)$_{25}$ lipid (pDLS18D, solvent: DMSO-d_6).

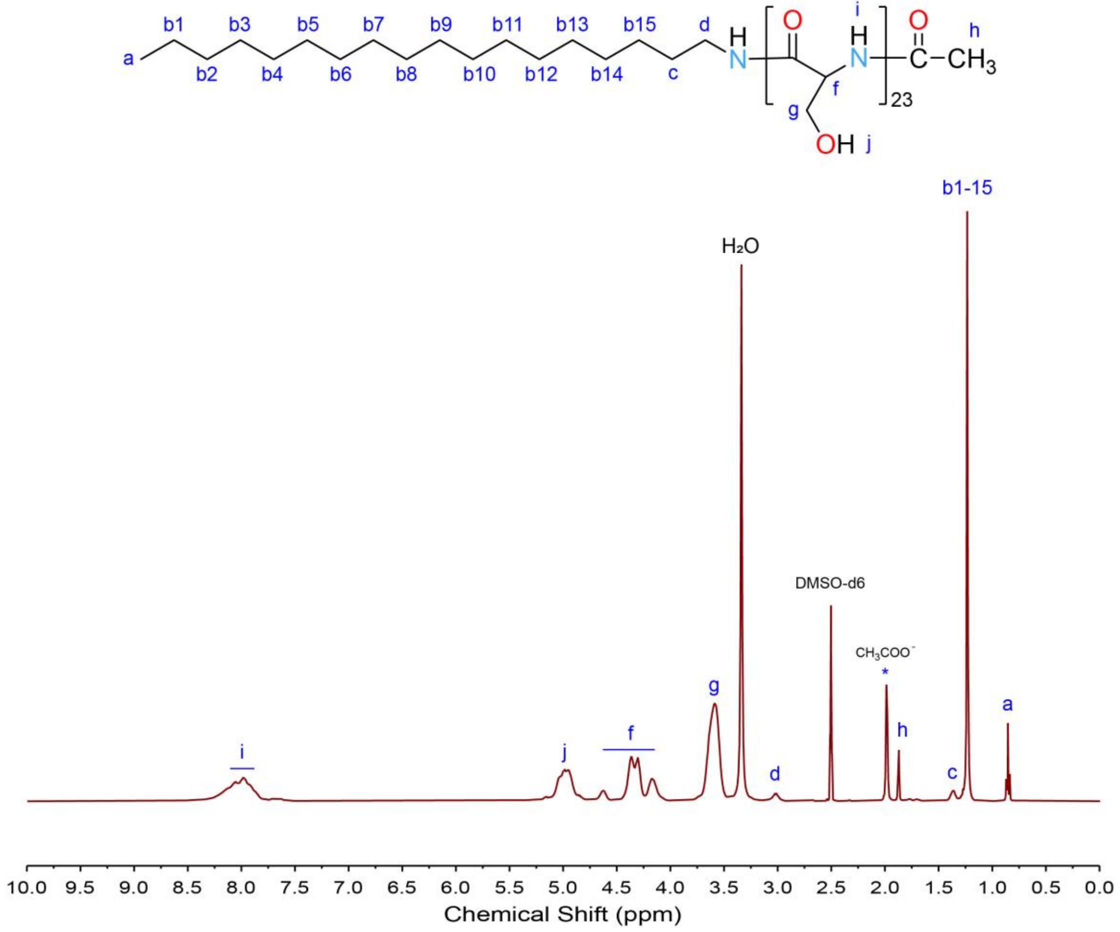

Supplementary Fig. 19 ^{1}H NMR spectrum of C18S-poly(D, L-Serine)$_{23}$ lipid (pDLS18S, solvent: DMSO-d_6).

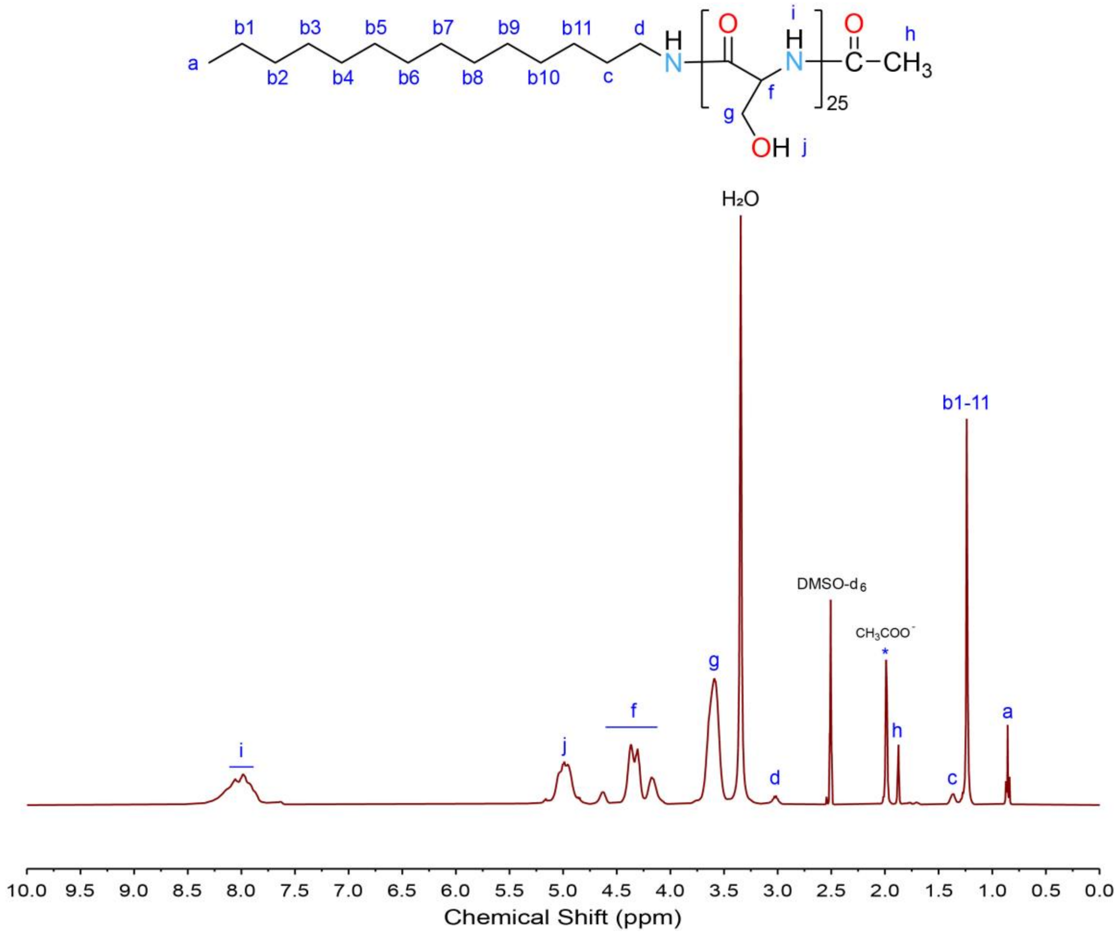

Supplementary Fig. 20 ^{1}H NMR spectrum of C14S-poly(D, L-Serine)$_{25}$ lipid (pDLS14S, solvent: DMSO-d_6).

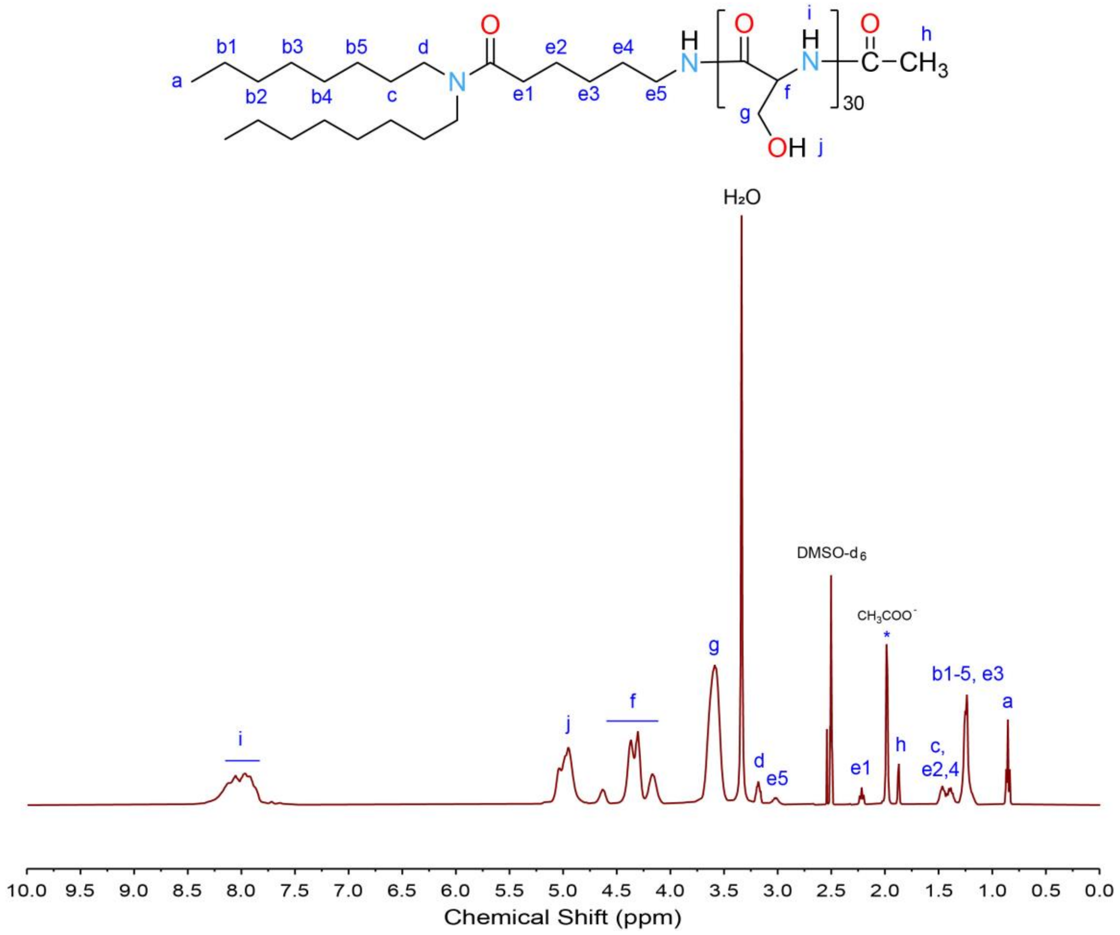

Supplementary Fig. 21 ^{1}H NMR spectrum of C8D-poly(D, L-Serine)$_{30}$ lipid (pDLS8D, solvent: DMSO-d_6).

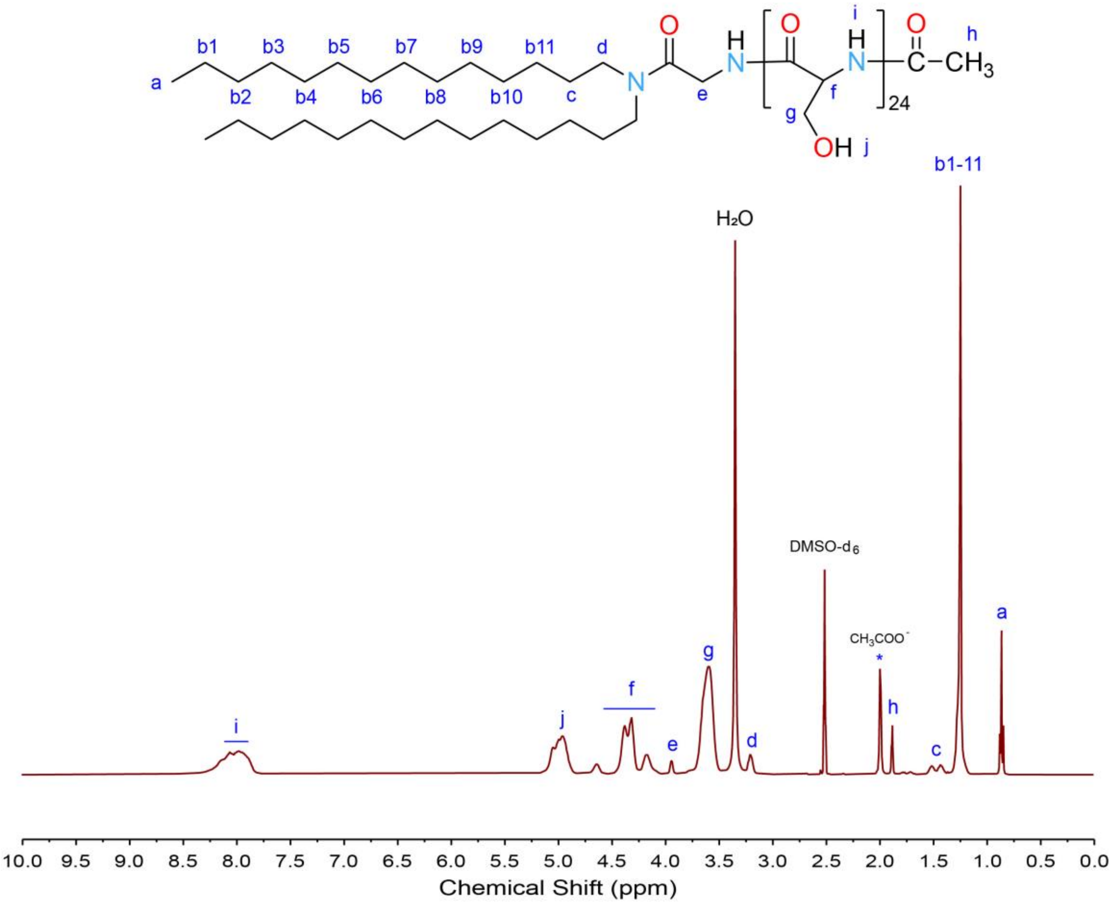

Supplementary Fig. 22 ^{1}H NMR spectrum of poly(D, L-Serine)$_{24}$ lipid (pDLS2 batch 2, solvent: DMSO-d_6).

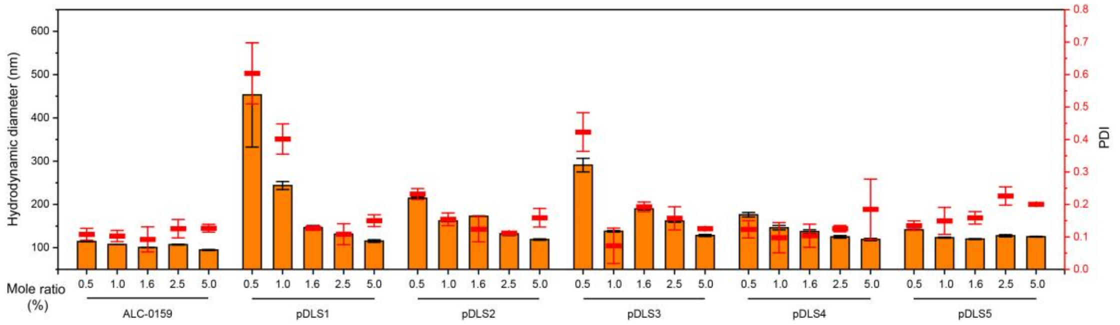

Supplementary Fig. 23 Hydrodynamic diameter and PDI of FLuc mRNA-loaded pDLS-LNPs prepared by pipette mixing using pDLS lipids with different DPs (n = 3 independent samples; mean ± SD). Source data are provided as a Source Data file.

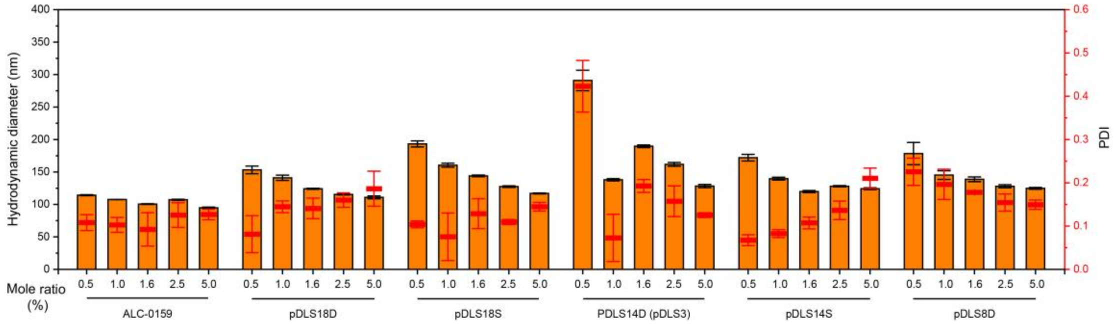

Supplementary Fig. 24 Hydrodynamic diameter and PDI of FLuc mRNA-loaded pDLS-LNPs prepared by pipette mixing using pDLS lipids with different hydrophobic tail structures (n = 3 independent samples; mean ± SD). Source data are provided as a Source Data file.

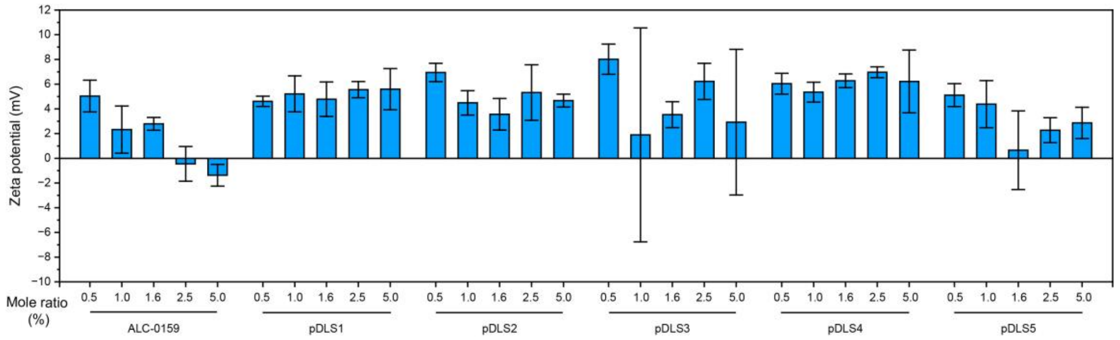

Supplementary Fig. 25 Zeta potential of FLuc mRNA-loaded pDLS-LNPs prepared by pipette mixing using pDLS lipids with different DPs (n = 3 independent samples; mean ± SD). Source data are provided as a Source Data file.

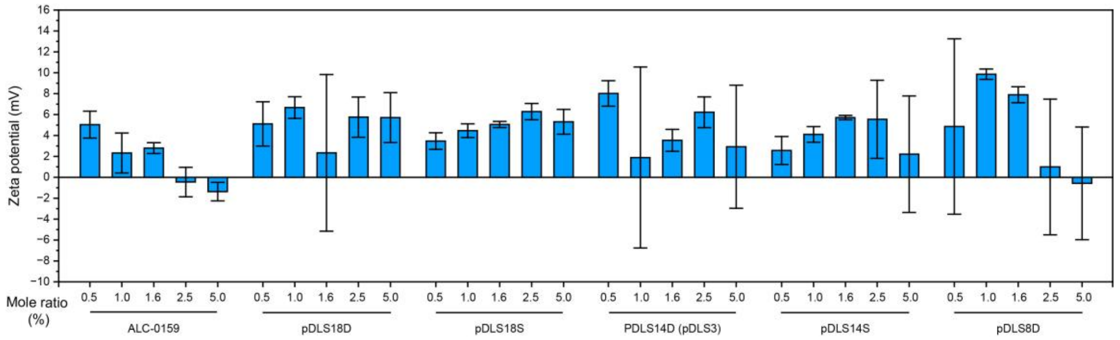

Supplementary Fig. 26 Zeta potential of FLuc mRNA-loaded pDLS-LNPs prepared by pipette mixing using pDLS lipids with different hydrophobic tail structures (n = 3 independent samples; mean ± SD). Source data are provided as a Source Data file.

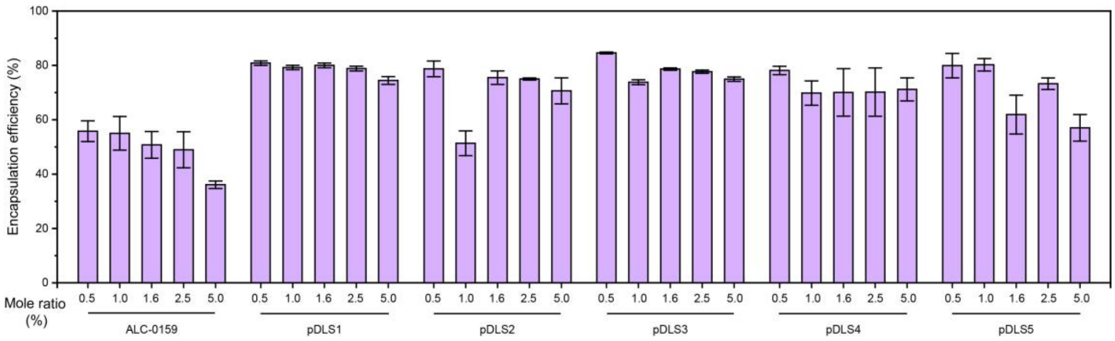

Supplementary Fig. 27 FLuc mRNA encapsulation efficiency of pDLS-LNPs prepared via pipette mixing using pDLS lipids with different DPs (*n* = 3 independent samples; mean ± SD). Source data are provided as a Source Data file.

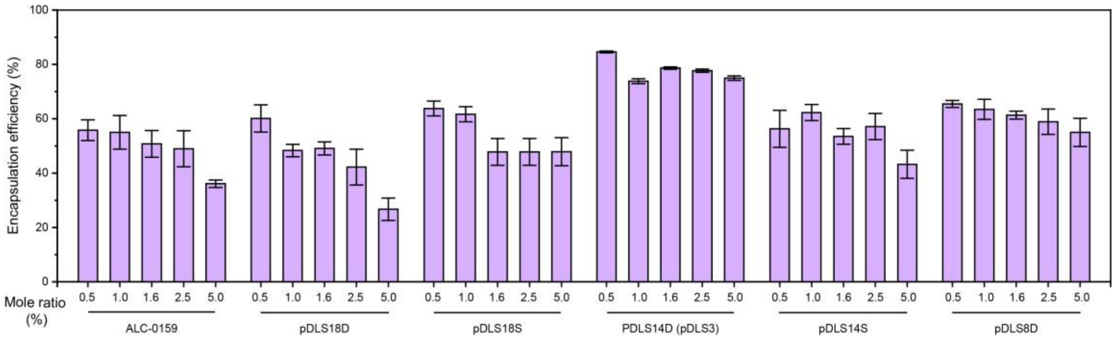

Supplementary Fig. 28 FLuc mRNA encapsulation efficiency of pDLS-LNPs prepared via pipette mixing using pDLS lipids with different hydrophobic tail structures (*n* = 3 independent samples; mean ± SD). Source data are provided as a Source Data file.

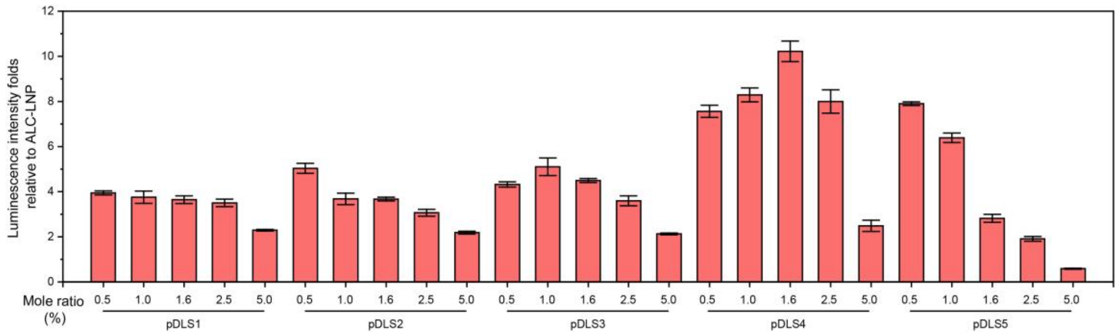

Supplementary Fig. 29 Screening of FLuc mRNA transfection efficiency in DC2.4 cells using pDLS-LNPs prepared via pipette mixing with pDLS lipids of varying DPs. DC2.4 cells were treated with FLuc mRNA-loaded LNPs at an mRNA dose of 100 ng per well in 96-well plates for 48 h (n = 3 independent biological samples; mean ± SD). Source data are provided as a Source Data file. Given the large number of pDLS lipids involved, the number of formulations that could be screened in each time was limited. Therefore, ALC-0159 at 1.6% was included as a positive control in every experiment. Due to variability introduced by manual mixing, the transfected luminescence intensity of the manually prepared ALC-0159 formulation varied between batches. Therefore, the luminescence intensity of pDLS LNPs was normalized to that of ALC-LNP prepared at 1.6%. The raw luminescence intensity results of FLuc mRNA-LNPs are listed in Supplementary Table 5.

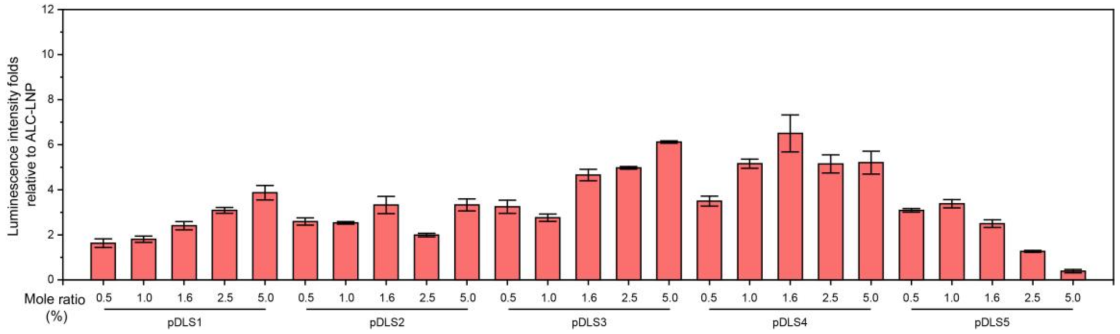

Supplementary Fig. 30 Screening of FLuc mRNA transfection efficiency in HEK 293T cells using pDLS-LNPs prepared via pipette mixing using pDLS lipids of varying DPs. HEK 293T cells were treated with FLuc mRNA-loaded LNPs at an mRNA dose of 100 ng per well in 96-well plates for 48 h (n = 3 independent biological samples; mean ± SD). Source data are provided as a Source Data file. Given the large number of pDLS lipids involved, the number of formulations that could be screened in each time was limited. Therefore, ALC-0159 at 1.6% was included as a positive control in every experiment. Due to variability introduced by manual mixing, the transfected luminescence intensity of the manually prepared ALC-0159 formulation varied between batches. Therefore, the luminescence intensity of pDLS LNPs was normalized to that of ALC-LNP prepared at 1.6%. The raw luminescence intensity results of FLuc mRNA-LNPs are listed in Supplementary Table 6.

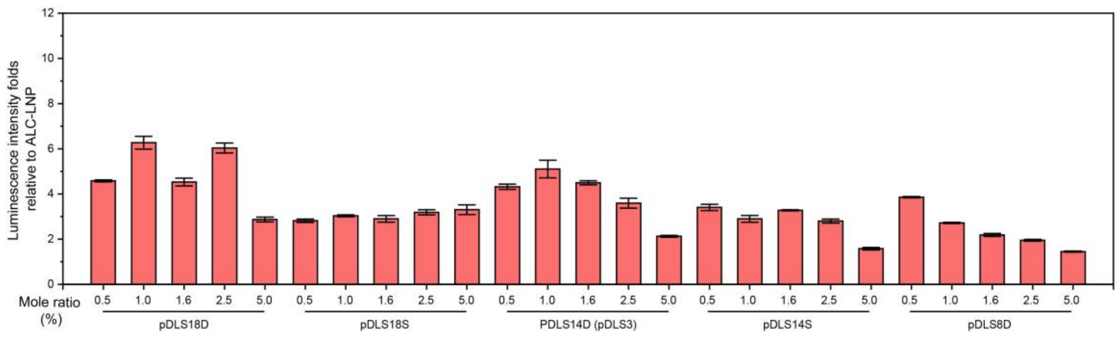

Supplementary Fig. 31 Screening of FLuc mRNA transfection efficiency in DC2.4 cells using pDLS-LNPs prepared via pipette mixing using pDLS lipids with different hydrophobic tail structures. DC2.4 cells were treated with FLuc mRNA-loaded LNPs at an mRNA dose of 100 ng per well in 96-well plates for 48 h (n = 3 independent biological samples; mean ± SD). Source data are provided as a Source Data file. Given the large number of pDLS lipids involved, the number of formulations that could be screened in each time was limited. Therefore, ALC-0159 at 1.6% was included as a positive control in every experiment. Due to variability introduced by manual mixing, the transfected luminescence intensity of the manually prepared ALC-0159 formulation varied between batches. Therefore, the luminescence intensity of pDLS LNPs was normalized to that of ALC-LNP prepared at 1.6%. The raw luminescence intensity results of FLuc mRNA-LNPs are listed in Supplementary Table 5.

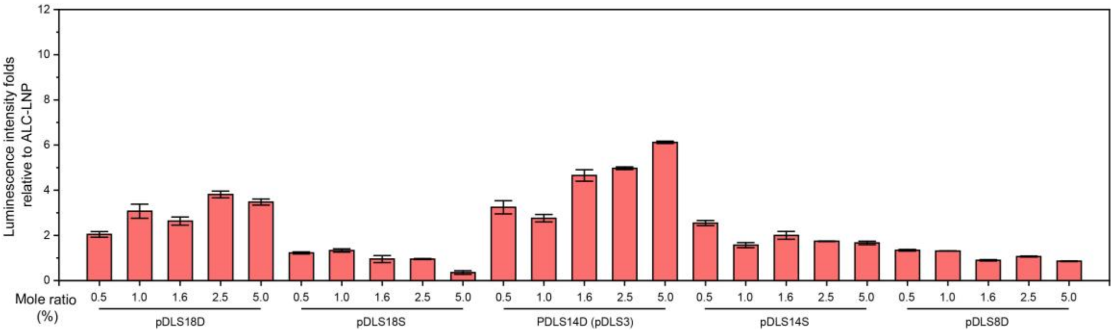

Supplementary Fig. 32 Screening of FLuc mRNA transfection efficiency in HEK 293T cells using pDLS-LNPs prepared via pipette mixing using pDLS lipids with different hydrophobic tail structures. HEK 293T cells were treated with FLuc mRNA-loaded LNPs at an mRNA dose of 100 ng per well in 96-well plates for 48 h (n = 3 independent biological samples; mean ± SD). Source data are provided as a Source Data file. Given the large number of pDLS lipids involved, the number of formulations that could be screened in each time was limited. Therefore, ALC-0159 at 1.6% was included as a positive control in every experiment. Due to variability introduced by manual mixing, the transfected luminescence intensity of the manually prepared ALC-0159 formulation varied between batches. Therefore, the luminescence intensity of pDLS LNPs was normalized to that of ALC-LNP prepared at 1.6%. The raw luminescence intensity results of FLuc mRNA-LNPs are listed in Supplementary Table 6.

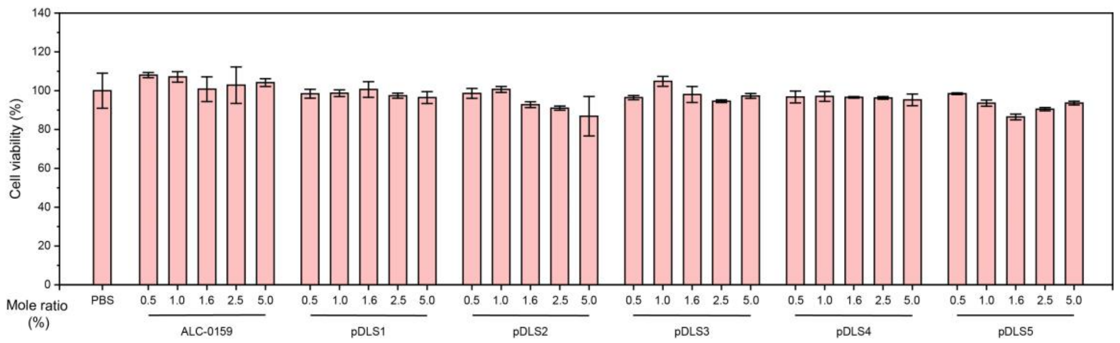

Supplementary Fig. 33 Viability of DC2.4 cells after treated with FLuc mRNA-loaded LNPs formulated via pipette mixing using pDLS lipids with different DPs. DC2.4 cells were treated with FLuc mRNA-loaded LNPs at an mRNA dose of 100 ng per well in 96-well plates for 48 h (*n* = 3 independent biological samples; mean ± SD). Source data are provided as a Source Data file.

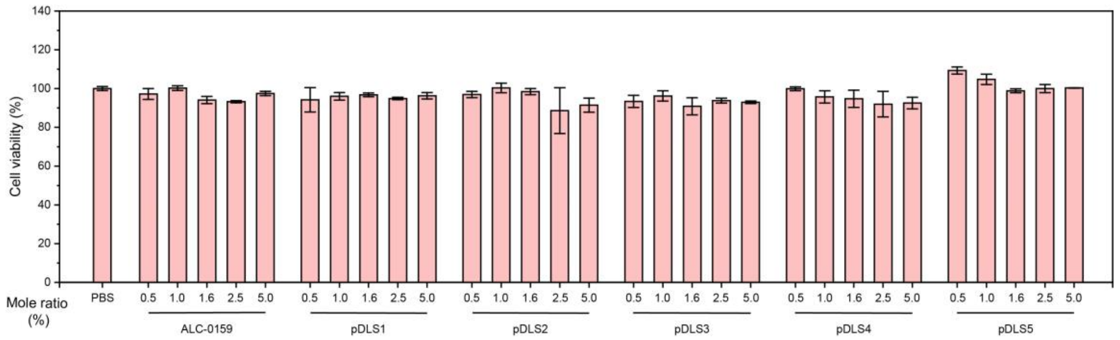

Supplementary Fig. 34 Viability of HEK 293T cells after treated with FLuc mRNA-loaded LNPs formulated via pipette mixing using pDLS lipids with different DPs. HEK 293T cells were treated with FLuc mRNA-loaded LNPs at an mRNA dose of 100 ng per well in 96-well plates for 48 h (*n* = 3 independent biological samples; mean ± SD). Source data are provided as a Source Data file.

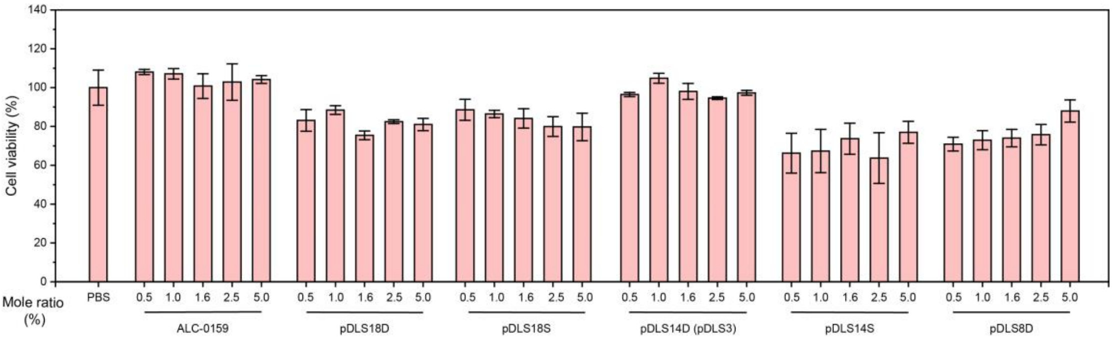

Supplementary Fig. 35 Viability of DC2.4 cells after treated with FLuc mRNA-loaded LNPs formulated via pipette mixing using pDLS lipids with different hydrophobic tail structures. DC2.4 cells were treated with FLuc mRNA-loaded LNPs at an mRNA dose of 100 ng per well in 96-well plates for 48 h (n = 3 independent biological samples; mean ± SD). Source data are provided as a Source Data file.

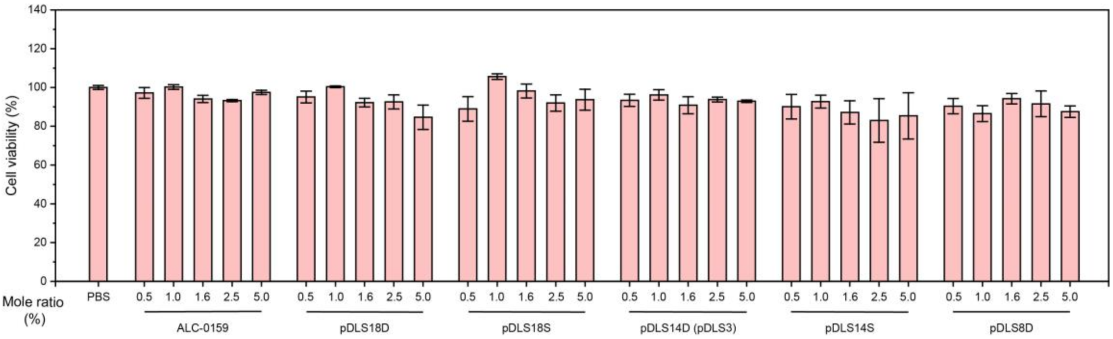

Supplementary Fig. 36 Viability of HEK 293T cells after treated with FLuc mRNA-loaded LNPs formulated via pipette mixing using pDLS lipids with different hydrophobic tail structures. HEK 293T cells were treated with FLuc mRNA-loaded LNPs at an mRNA dose of 100 ng per well in 96-well plates for 48 h (n = 3 independent biological samples; mean ± SD). Source data are provided as a Source Data file.

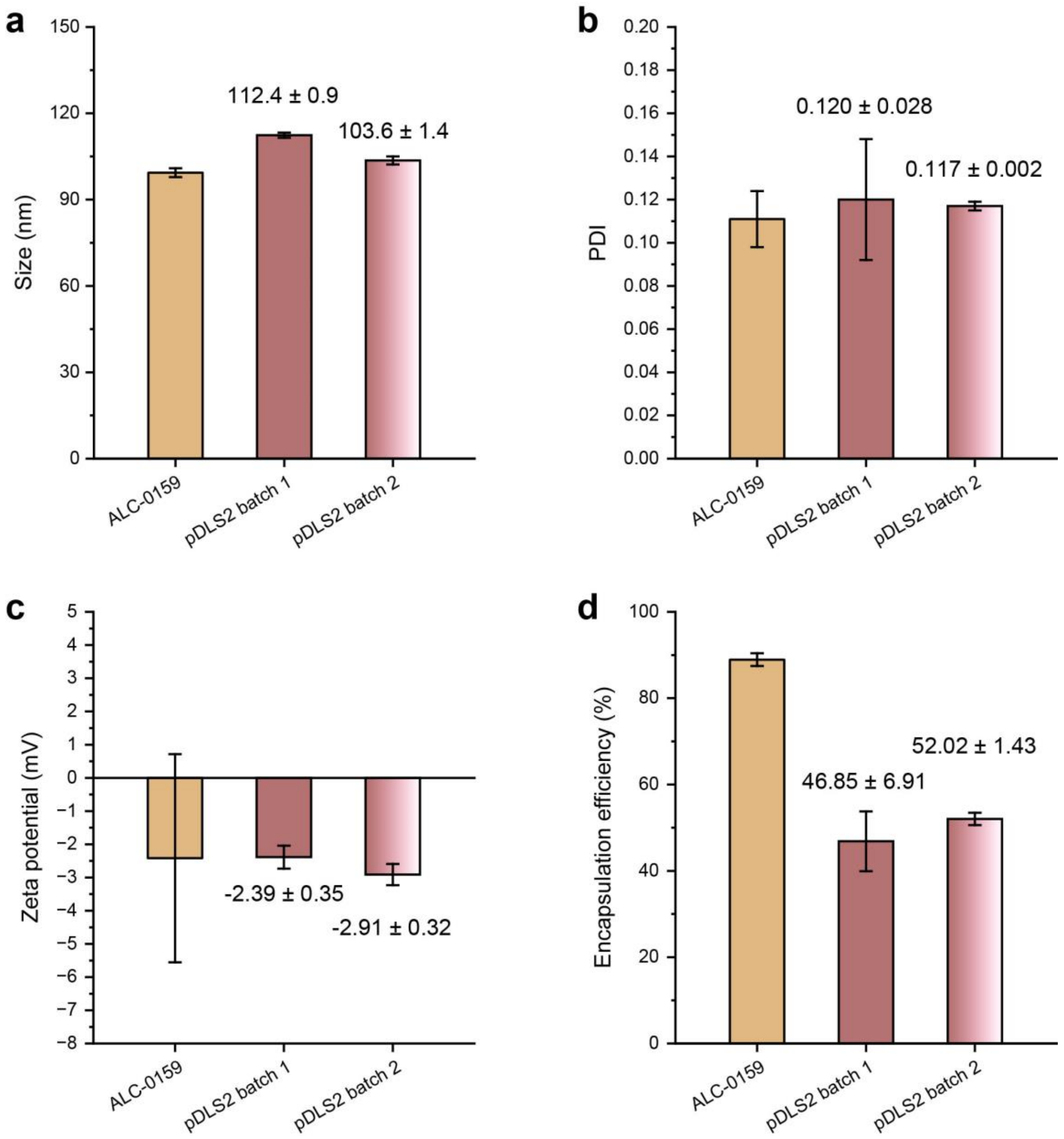

Supplementary Fig. 37 Hydrodynamic diameter (**a**), PDI (**b**), zeta potential (**c**) and FLuc mRNA encapsulation efficiency (**d**) of LNPs formulated with different batches of pDLS2 lipid (*n* = 3 independent samples; mean ± SD; FLuc mRNA-loaded LNPs were prepared *via* pipette mixing). Source data are provided as a Source Data file.

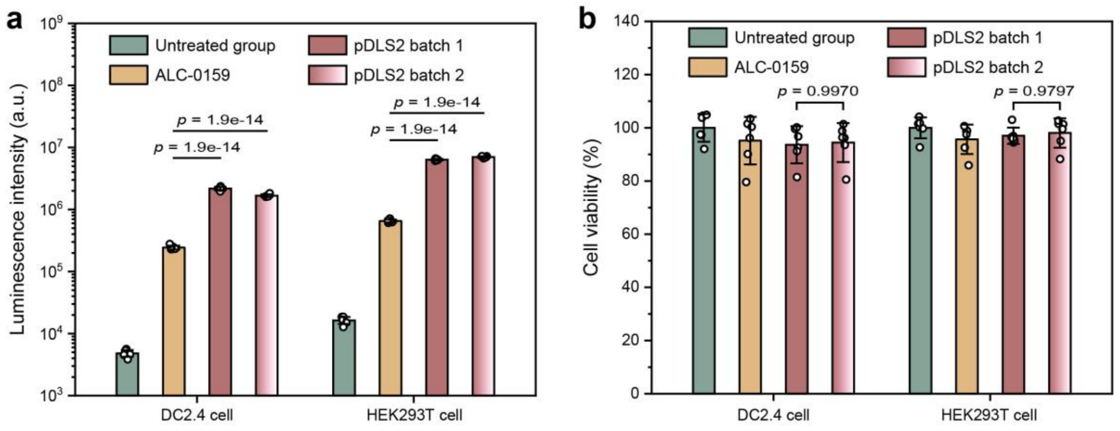

Supplementary Fig. 38 Transfection efficiency of FLuc mRNA-loaded LNPs prepared using different batches of pDLS2 lipid (**a**) and viability (**b**) of HEK 293T and DC2.4 cells (n = 5 independent biological samples; mean ± SD; FLuc mRNA-loaded LNPs were prepared *via* pipette mixing). Statistical analyses were performed using two-tailed one-way ANOVA with Tukey's correction (**a-b**). Source data are provided as a Source Data file.

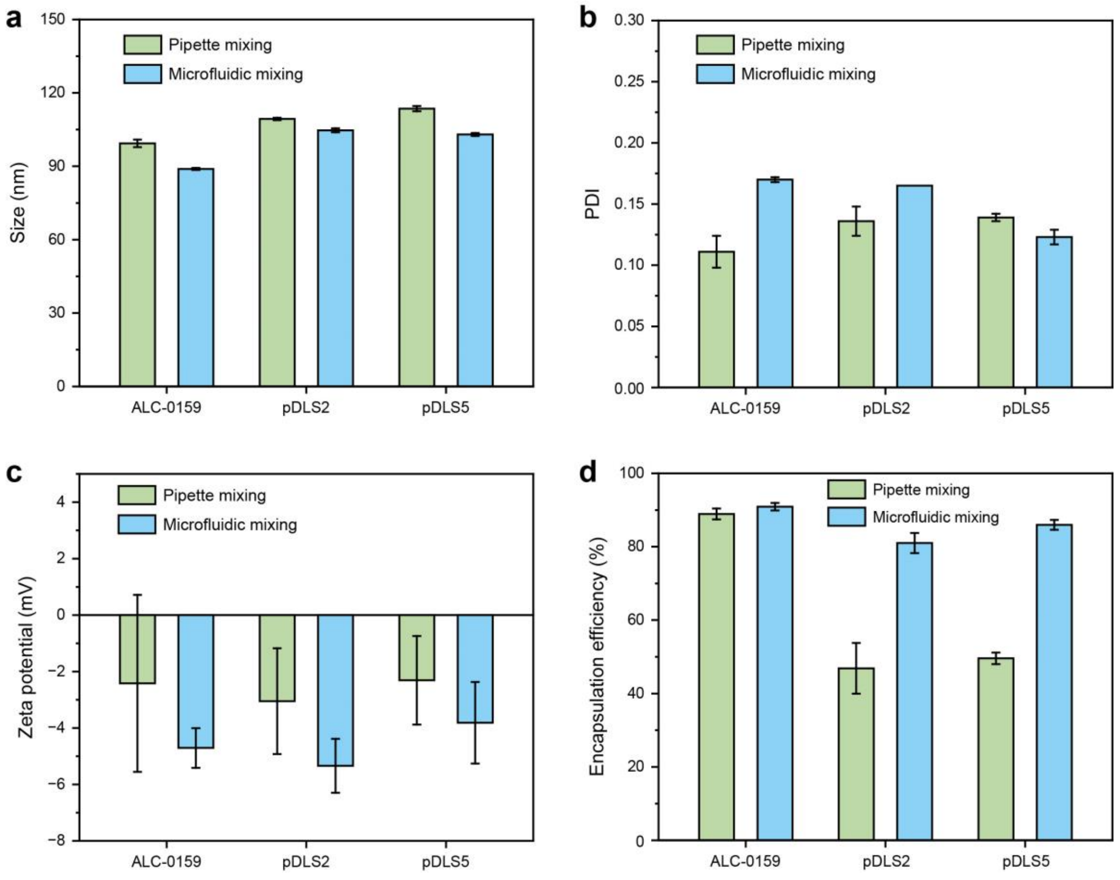

Supplementary Fig. 39 Hydrodynamic diameter (**a**), PDI (**b**), zeta potential (**c**) and FLuc mRNA encapsulation efficiency (**d**) of LNPs formulated using different mixing methods (pipette *vs.* microfluidic mixing) (n = 3 independent samples; mean ± SD). Source data are provided as a Source Data file.

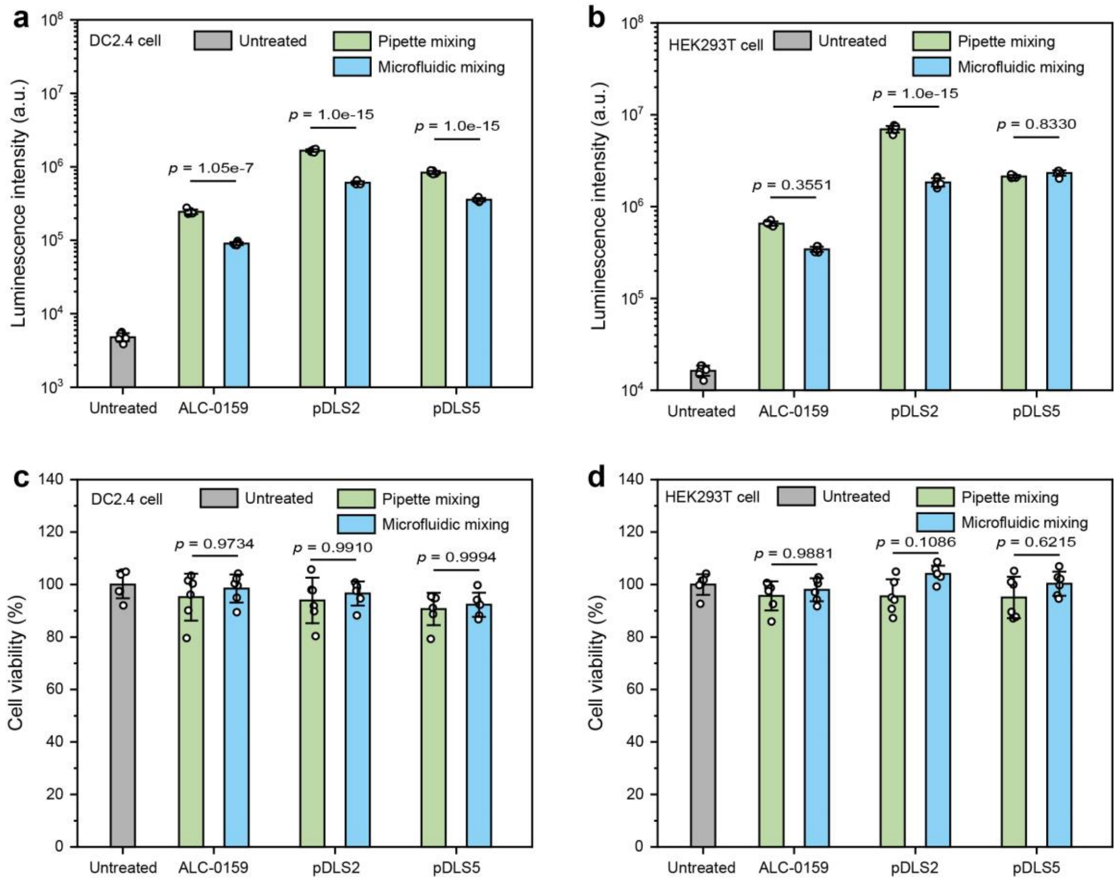

Supplementary Fig. 40 FLuc mRNA transfection efficiency in DC2.4 cells (**a**) and HEK 293T cells (**b**), and viability of DC2.4 cells (**c**) and HEK 293T cells (**d**) of LNPs using different mixing methods (pipette *vs.* microfluidic mixing) (n = 5 independent biological samples; mean ± SD). Statistical analyses were performed using two-tailed one-way ANOVA with Tukey's correction (**a-d**). Source data are provided as a Source Data file.

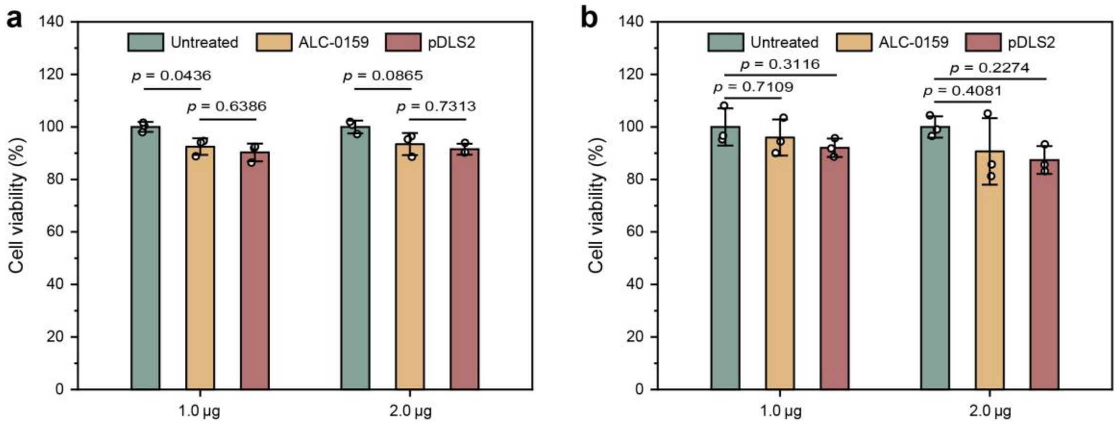

Supplementary Fig. 41 Viability of DC2.4 cells (**a**) and HEK 293T cells (**b**) after 48 h of treatment with ALC-LNP and pDLS2-LNP encapsulating Cy5-labeled mRNA at doses of 1000 ng and 2000 ng per well in 24-well plates (n = 3 independent biological samples; mean ± SD; Both LNPs were prepared *via* pipette mixing). Statistical analyses were performed using two-tailed one-way ANOVA with Tukey's correction (**a**-**b**). Source data are provided as a Source Data file.

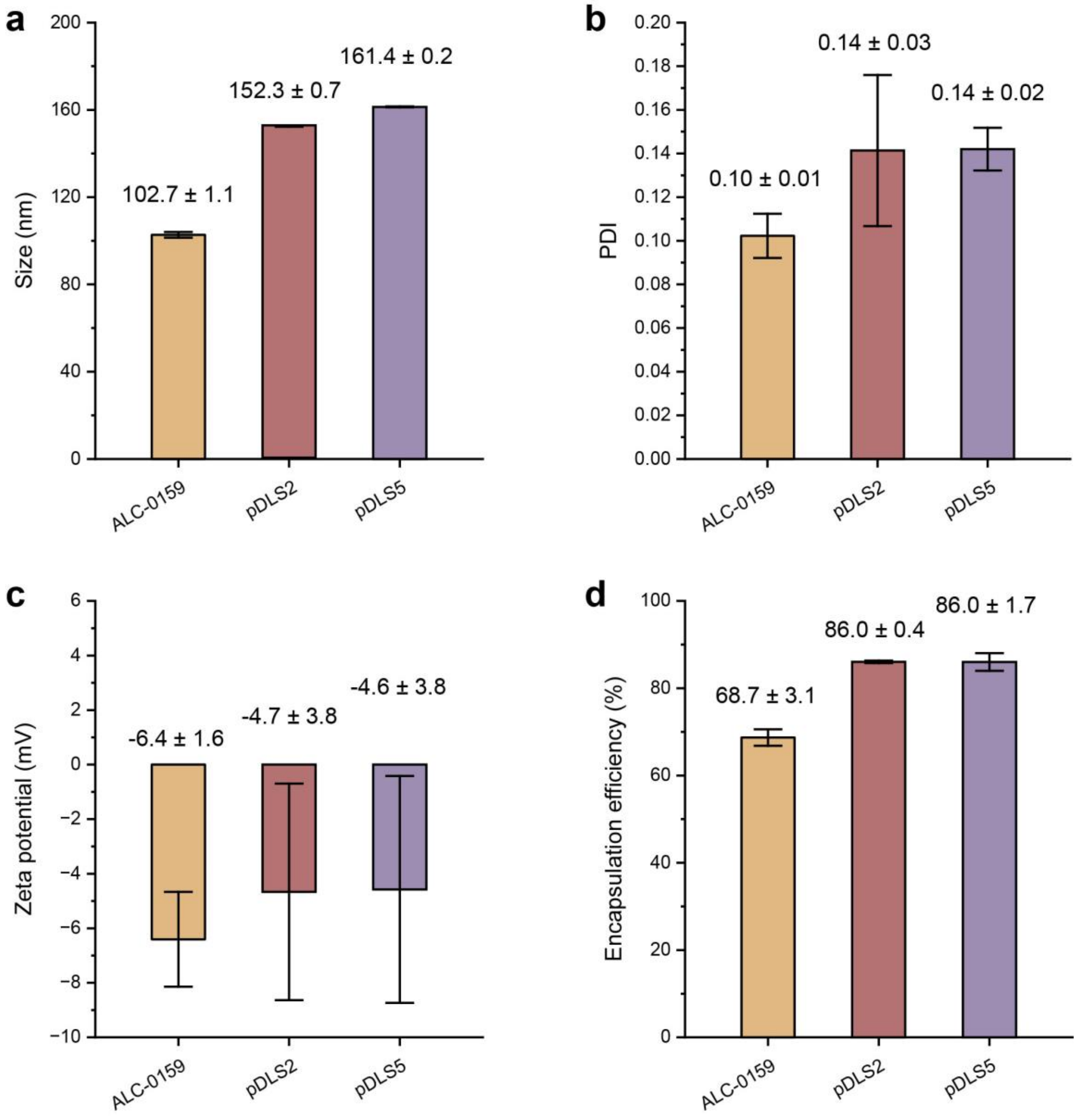

Supplementary Fig. 42 Hydrodynamic diameter (**a**), PDI (**b**), zeta potential (**c**) and siGFP encapsulation efficiency (**d**) of ALC-LNP, pDLS2-LNP and pDLS5-LNP (n = 3 independent samples; mean ± SD; FLuc mRNA-loaded LNPs were prepared via pipette mixing). Source data are provided as a Source Data file.

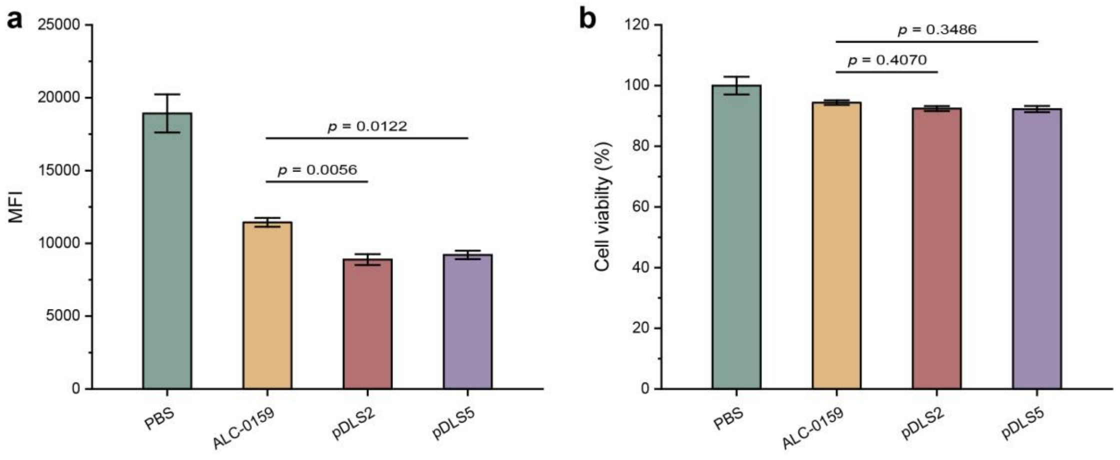

Supplementary Fig. 43 *GFP* gene knockdown efficiency (**a**) and viability (**b**) of GFP-expressing HEK293 (HEK293-GFP) cells after 48 h of treatment with siGFP-loaded LNPs (n = 3 independent biological samples; mean ± SD; siGFP-loaded LNPs were prepared via pipette mixing). Statistical analyses were performed using two-tailed one-way ANOVA with Tukey's correction (**a**, **b**). Source data are provided as a Source Data file.

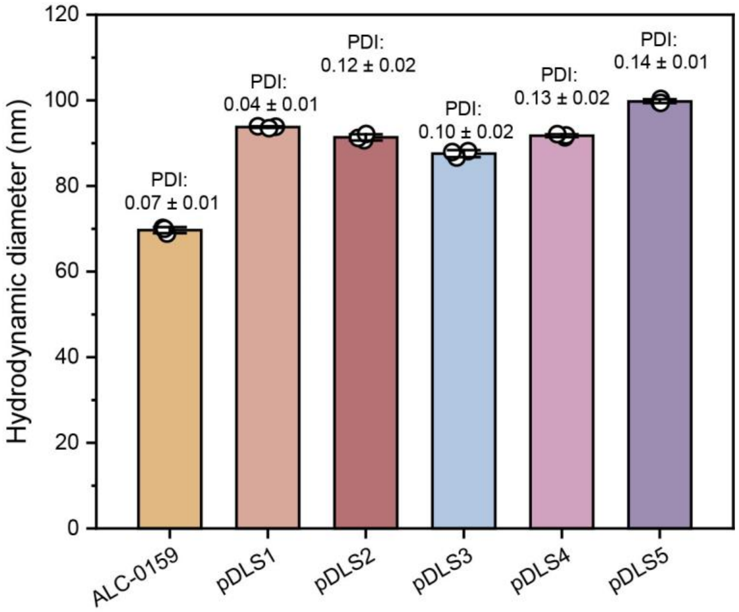

Supplementary Fig. 44 Hydrodynamic diameter and PDI of SARS-CoV-2 spike mRNA-loaded LNPs prepared via microfluidic mixing (*n* = 3 independent samples; mean ± SD). Source data are provided as a Source Data file.

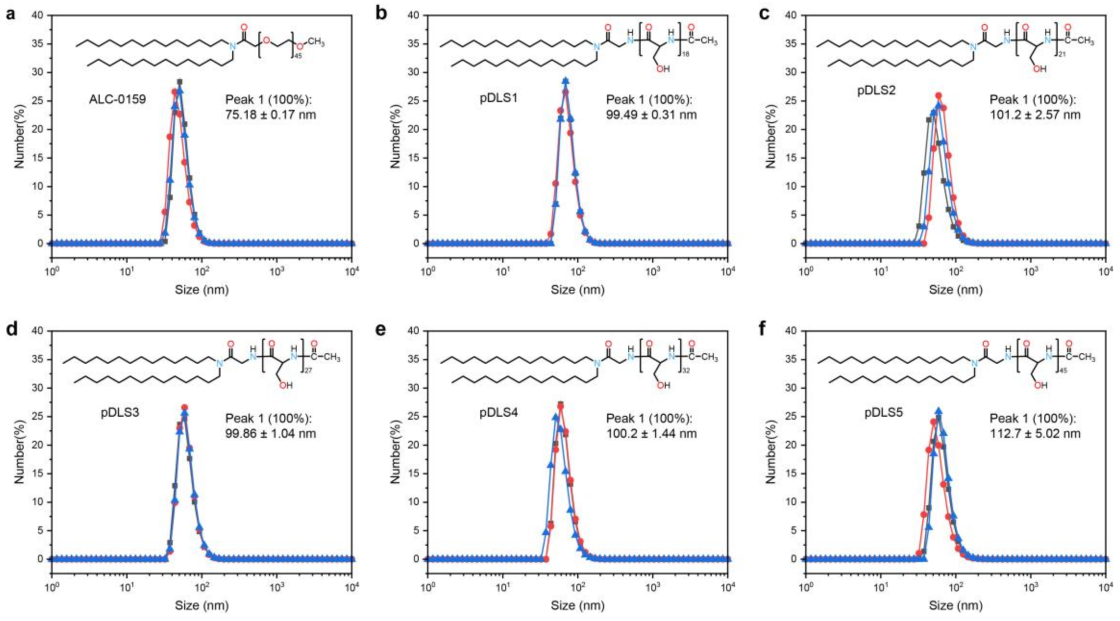

Supplementary Fig. 45 Size distributions by number of SARS-CoV-2 spike mRNA-loaded LNPs prepared via microfluidic mixing: **a** ALC-LNP; **b** pDLS1-LNP; **c** pDLS2-LNP; **d** pDLS3-LNP; **e** pDLS4-LNP; **f** pDLS5-LNP (*n* = 3 independent samples; mean ± SD). Source data are provided as a Source Data file.

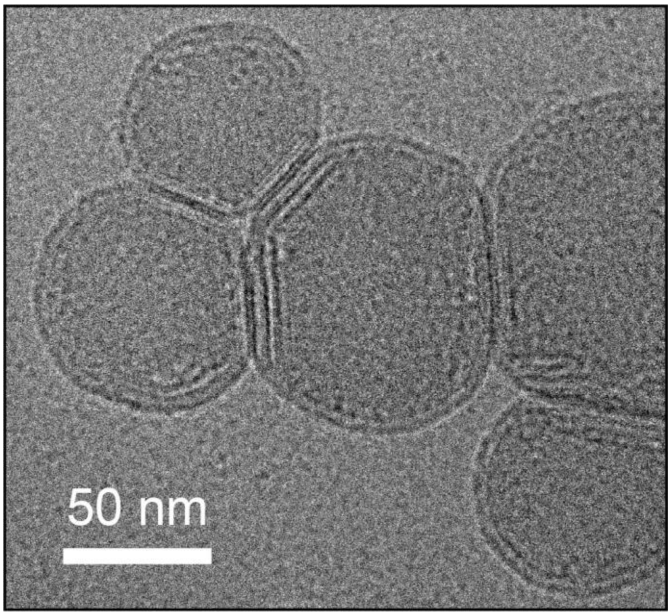

Supplementary Fig. 46 Cryo-TEM image of representative FLuc mRNA-loaded pDLS2-LNPs prepared via microfluidic mixing.

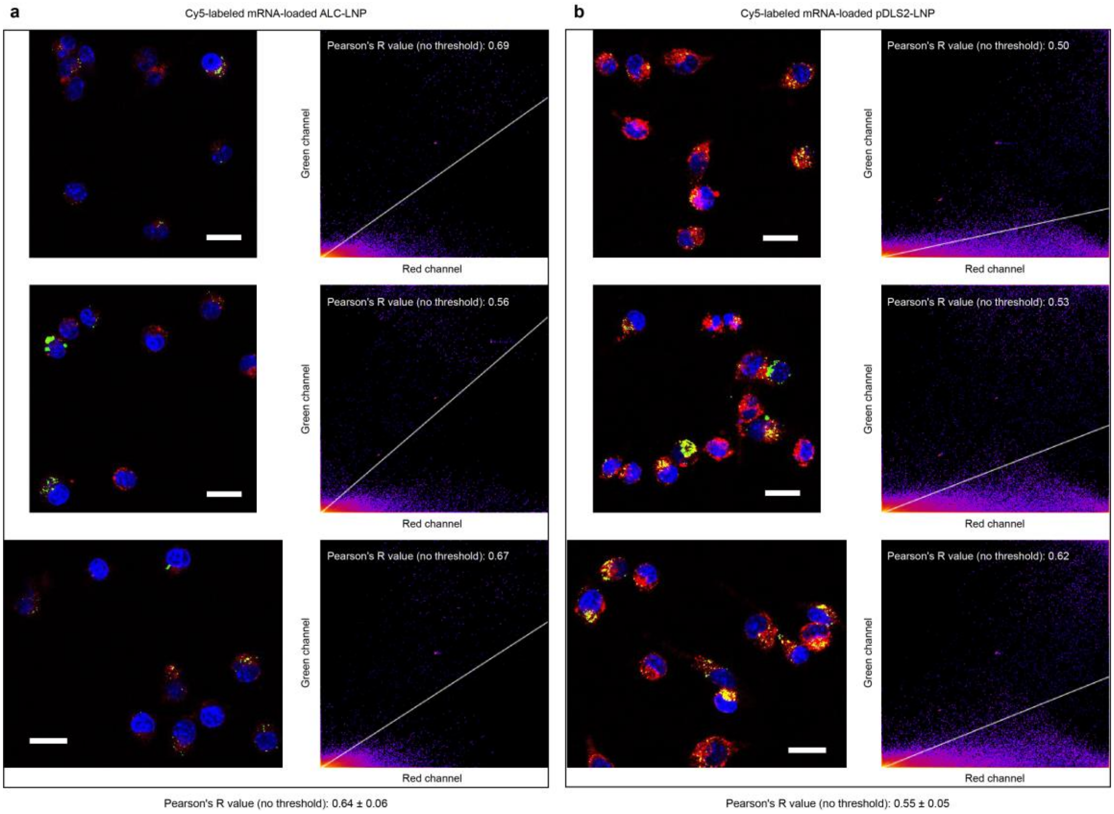

Supplementary Fig. 47 Pixel-wise colocalization analysis based on 2D intensity histogram of representative confocal images of DC2.4 cells treated with **a** Cy5 mRNA-loaded ALC-LNP, and **b** Cy5 mRNA-loaded pDLS2-LNP. (Cy5-labeled mRNA-loaded LNPs were prepared via pipette mixing). DC2.4 cells treated with LNPs encapsulated with Cy5-labelled mRNA (Red) for 4 h, followed by staining with LysoTracker Green (Green) and Hoechst 33342 (Blue). Scale bar, 20 μm. In pixel-wise colocalization analyses, the intensity of a pixel in one channel is evaluated against the corresponding pixel in the second channel of a dual-color image, generally producing a scatterplot from which a correlation coefficient is determined. The pixel-wise matching means that there must be overlap of the signal from the two channels to demonstrate positive spatial correlation (n = 3 independent biological samples; mean ± SD). Source data are provided as a Source Data file.

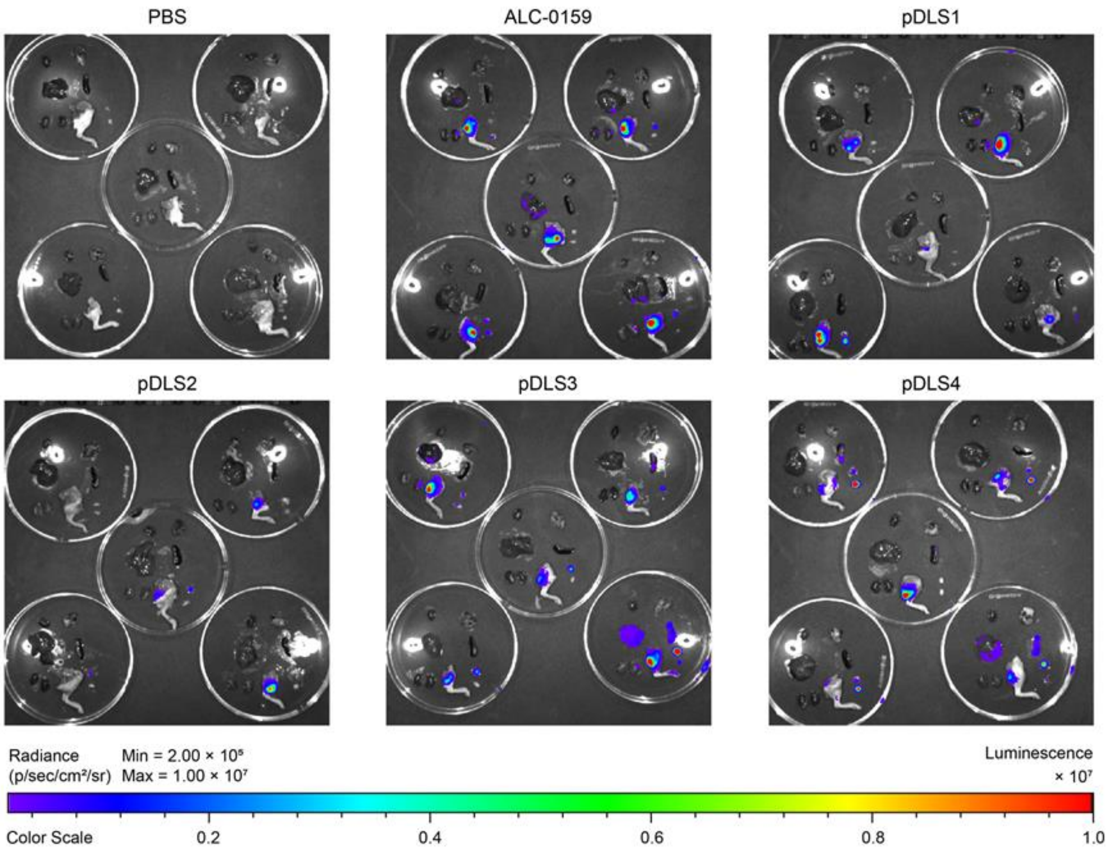

Supplementary Fig. 48 Ex vivo bioluminescence images of major organs collected at 24 h post-s.c. injection of ALC-LNP, pDLS1-, pDLS2-, pDLS3- or pDLS4-LNP.

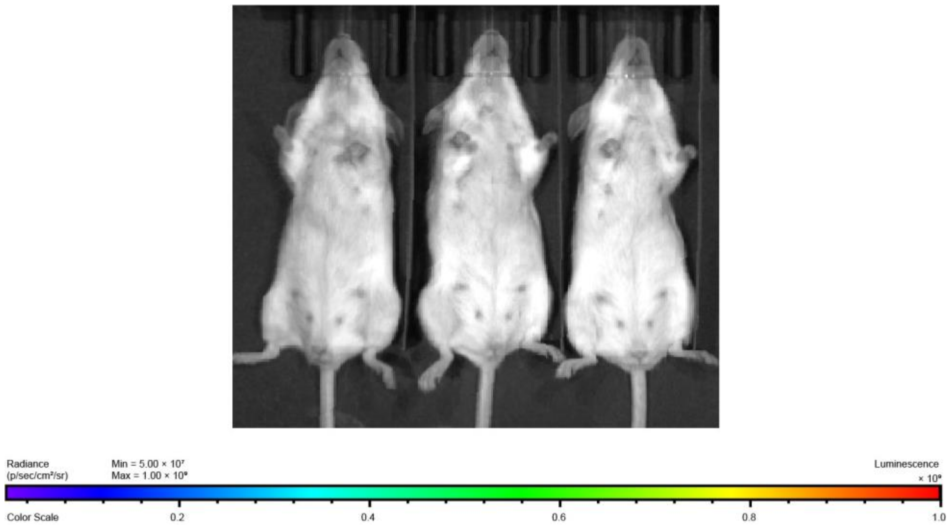

Supplementary Fig. 49 In vivo bioluminescence images at 6 h after i.v. injection of PBS (n = 3 mice for PBS group).

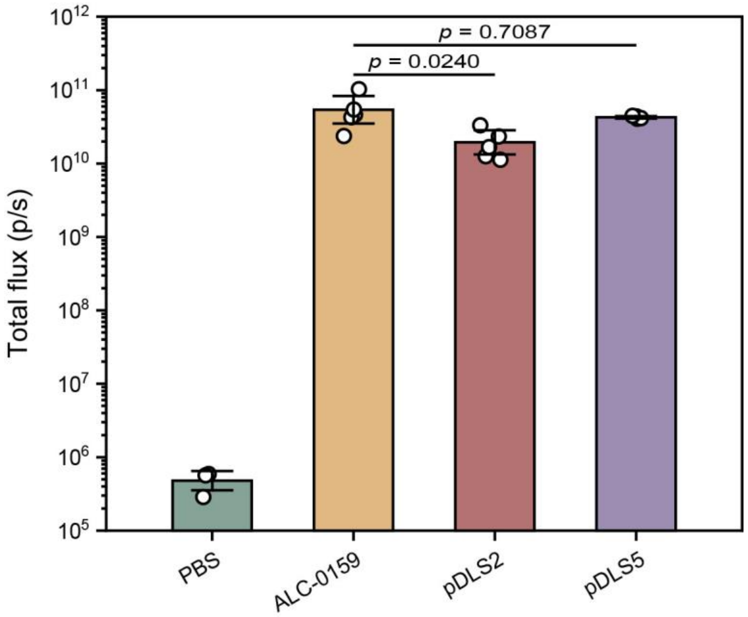

Supplementary Fig. 50 Quantification of relative bioluminescence radiance intensity (RBRI) for mice treated with different FLuc mRNA-loaded LNPs at 6 h post-i.v. administration (n = 3 mice for PBS group; n = 5 mice for ALC-LNP, pDLS2-LNP and pDLS5-LNP groups; mean ± SD). Statistical analyses were performed using two-tailed one-way ANOVA with Tukey's correction. Source data are provided as a Source Data file.

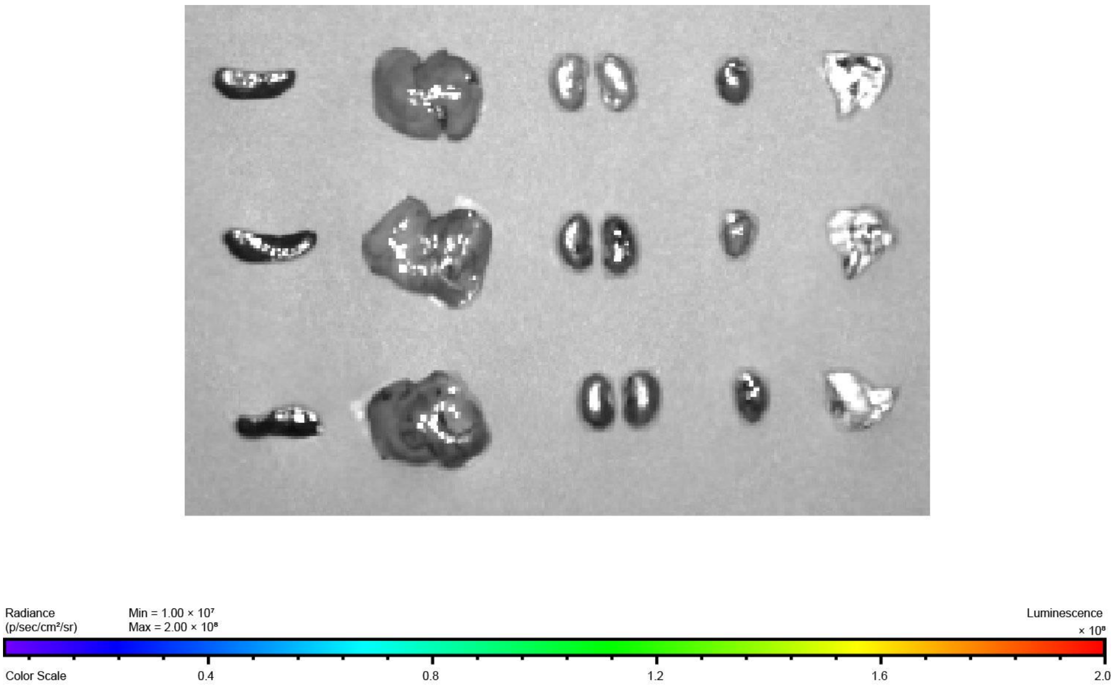

Supplementary Fig. 51 Ex vivo bioluminescence images of major organs collected at 6 h post-i.v. injection of PBS (*n* = 3 mice for PBS group).

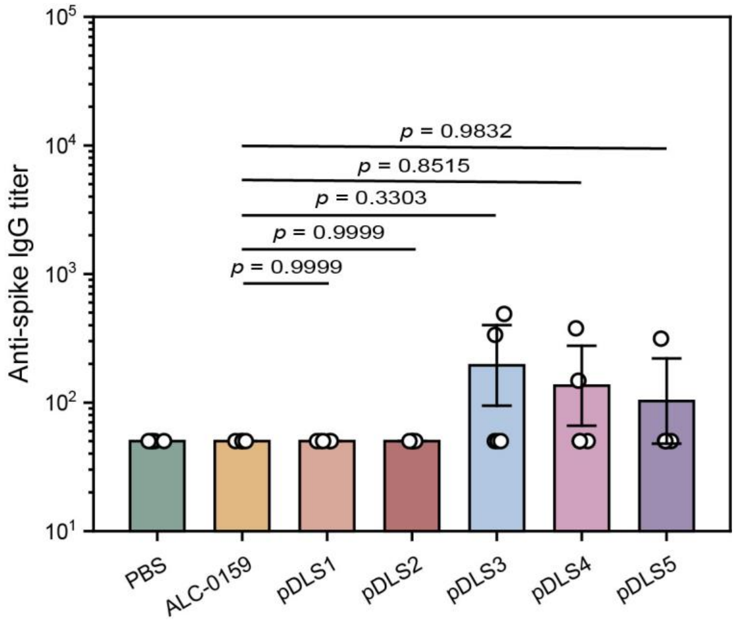

Supplementary Fig. 52 Anti-spike IgG titers in mice treated with ALC-LNP or pDLS-LNPs at week 2. BALB/c mice were s.c. immunized with SARS-CoV-2 mRNA-loaded LNPs (3 μg mRNA per mouse) at week 0 (n = 5 independent biological samples; mean ± SD). Statistical analyses were performed using two-tailed one-way ANOVA with Tukey's correction. Source data are provided as a Source Data file.

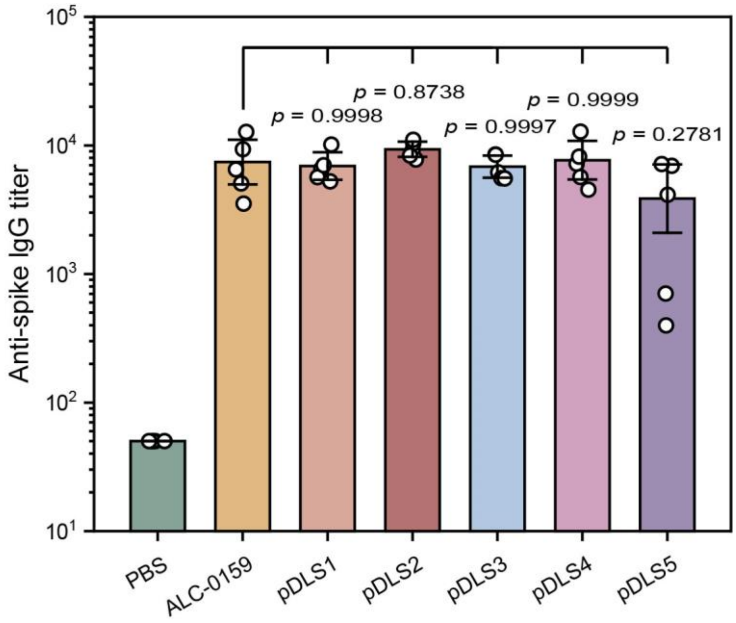

Supplementary Fig. 53 Anti-spike IgG titers at week 4. BALB/c mice were s.c. immunized with SARS-CoV-2 mRNA-loaded LNPs (3 μg mRNA per mouse) at week 0 and 3 (n = 5 independent biological samples; mean ± SD). Statistical analyses were performed using two-tailed one-way ANOVA with Tukey's correction. Source data are provided as a Source Data file.

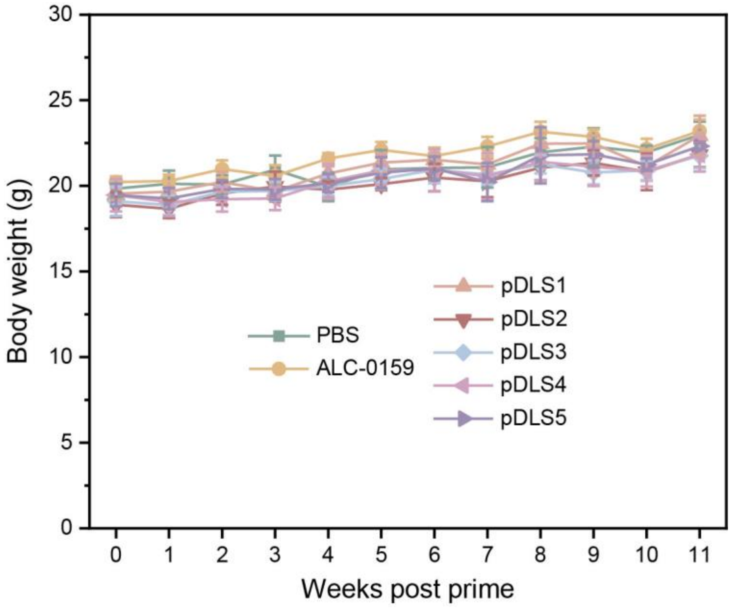

Supplementary Fig. 54 Body weight of mice monitored over time. BALB/c mice were s.c. immunized with SARS-CoV-2 mRNA-loaded LNPs (3 µg mRNA per mouse) at weeks 0 and 3 (*n* = 5 independent biological samples; mean ± SD). Source data are provided as a Source Data file.

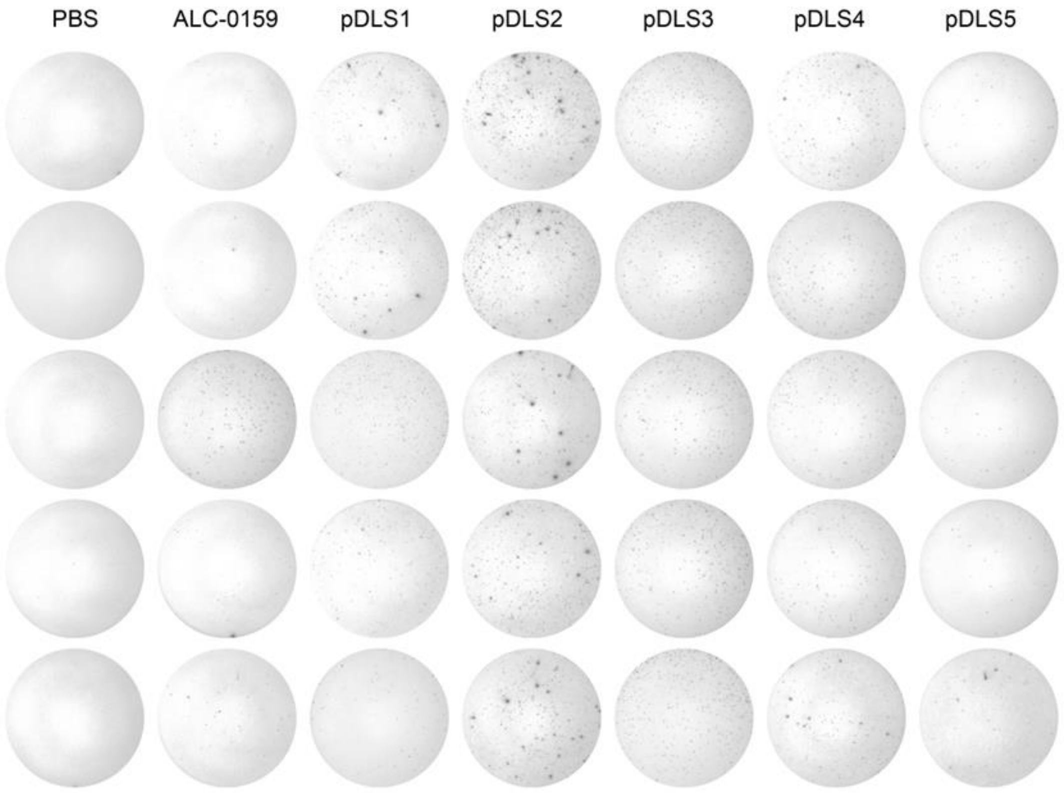

Supplementary Fig. 55 ELISpot images showing RBD-specific IgG1^{+} cells in spleens. BALB/c mice were s.c. immunized with SARS-CoV-2 mRNA-loaded LNPs (3 µg mRNA) at week 0 and 3. Spleens were collected at the end of the experiment for ELISpot analysis (*n* = 5 independent biological samples; mean ± SD).

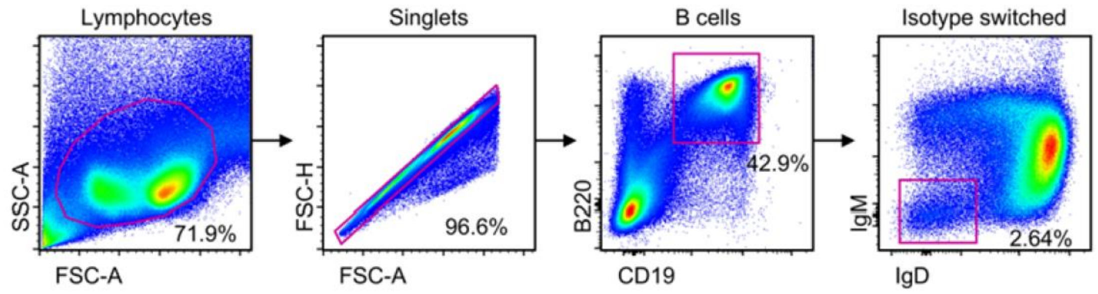

Supplementary Fig. 56 Gating scheme for the identification of IgD^{-}IgM^{-} isotype-switched B cells.

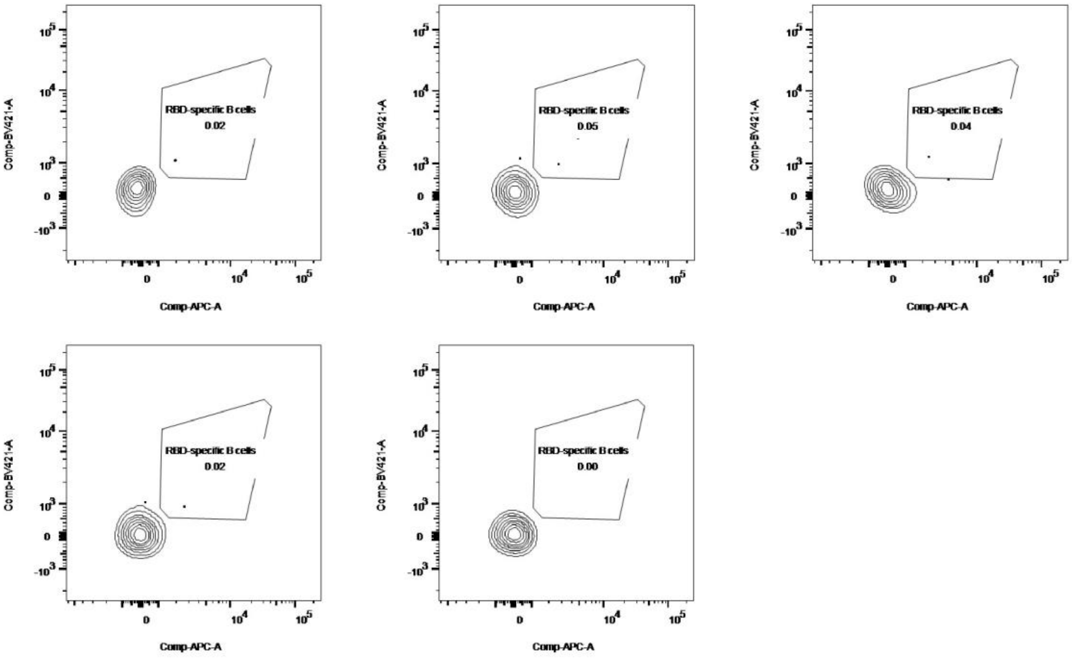

Supplementary Fig. 57 Gating analysis for the identification of $IgD^{-}IgM^{-}RBD^{+}$ B cells in spleens from the mice of PBS group.

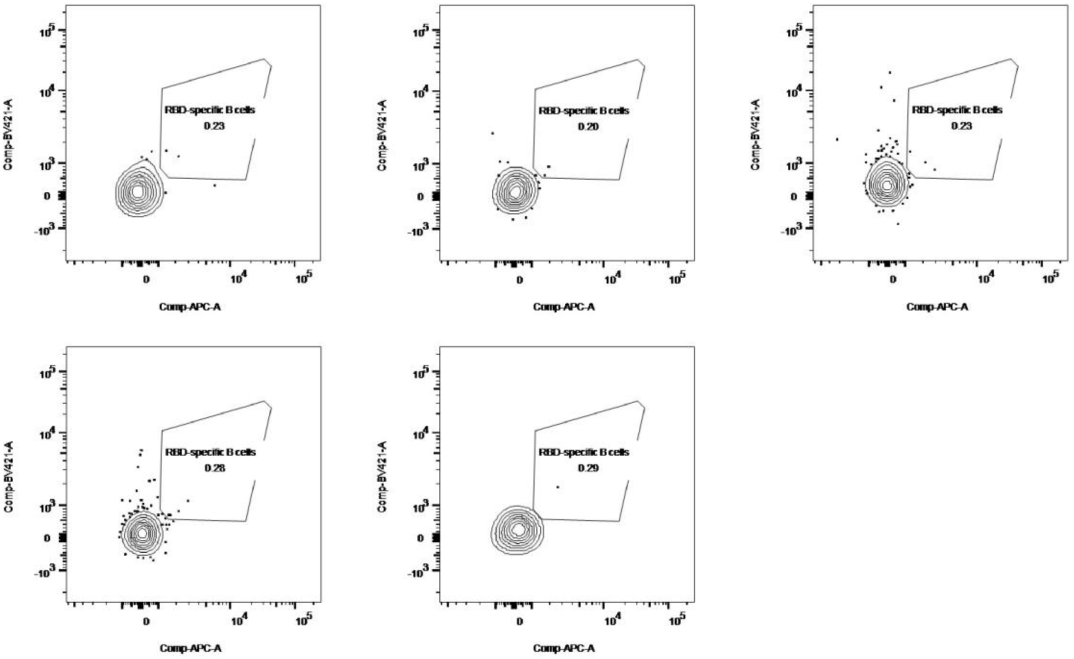

Supplementary Fig. 58 Gating analysis for the identification of $IgD^{-}IgM^{-}RBD^{+}$ B cells in spleens from the mice of ALC-LNP group.

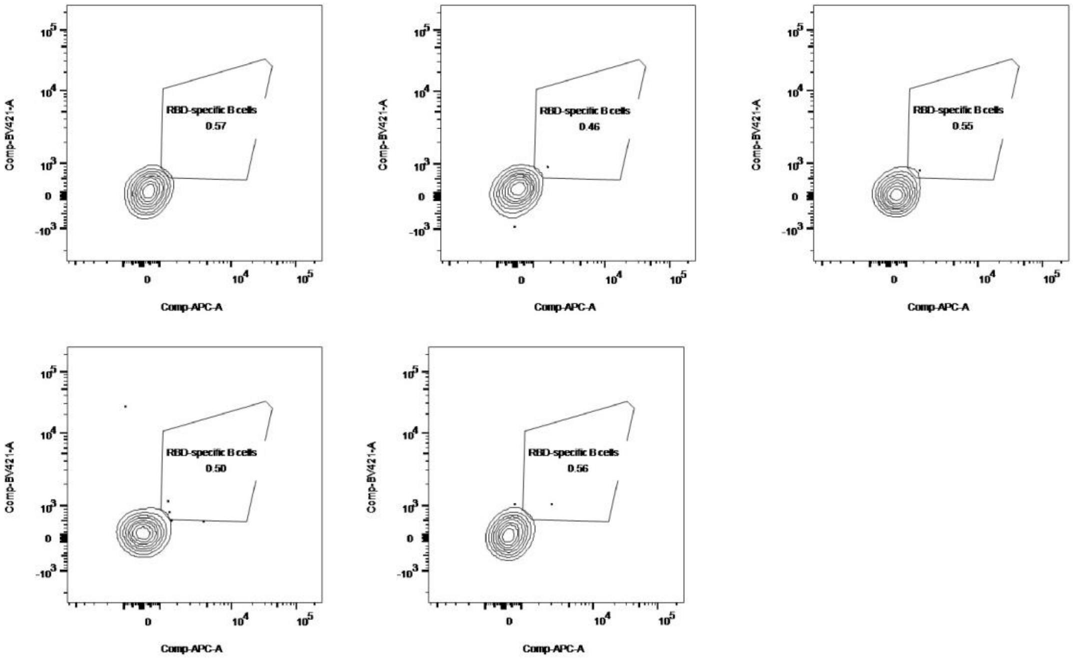

Supplementary Fig. 59 Gating analysis for the identification of IgD^{-}IgM^{-}RBD^{+} B cells in spleens from the mice of pDLS1-LNP group.

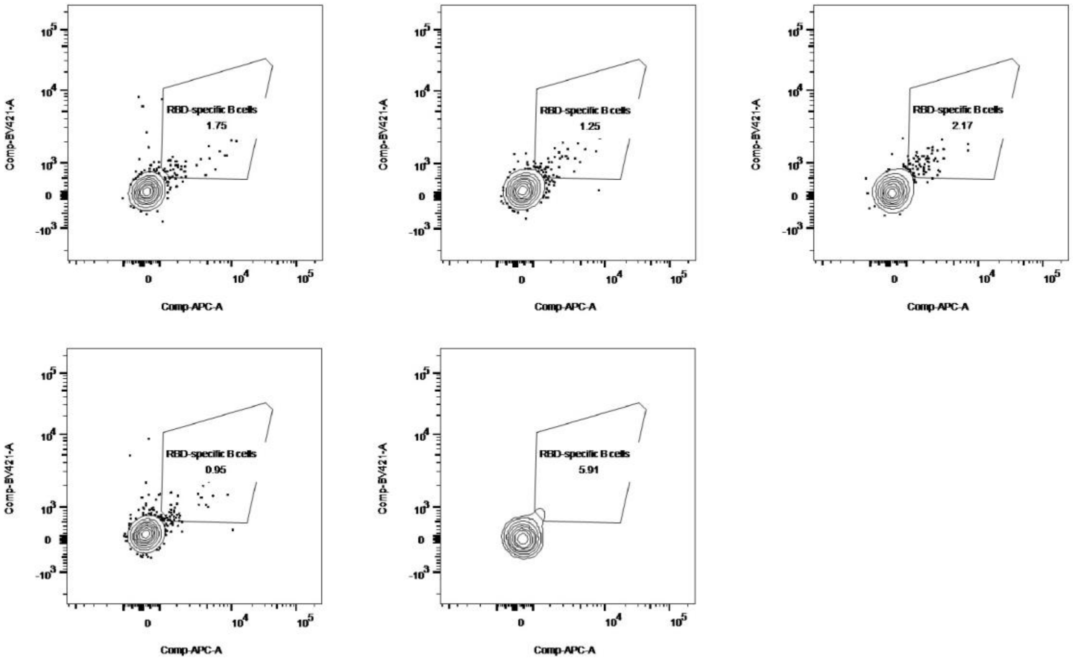

Supplementary Fig. 60 Gating analysis for the identification of IgD^{-}IgM^{-}RBD^{+} B cells in spleens from the mice of pDLS2-LNP group.

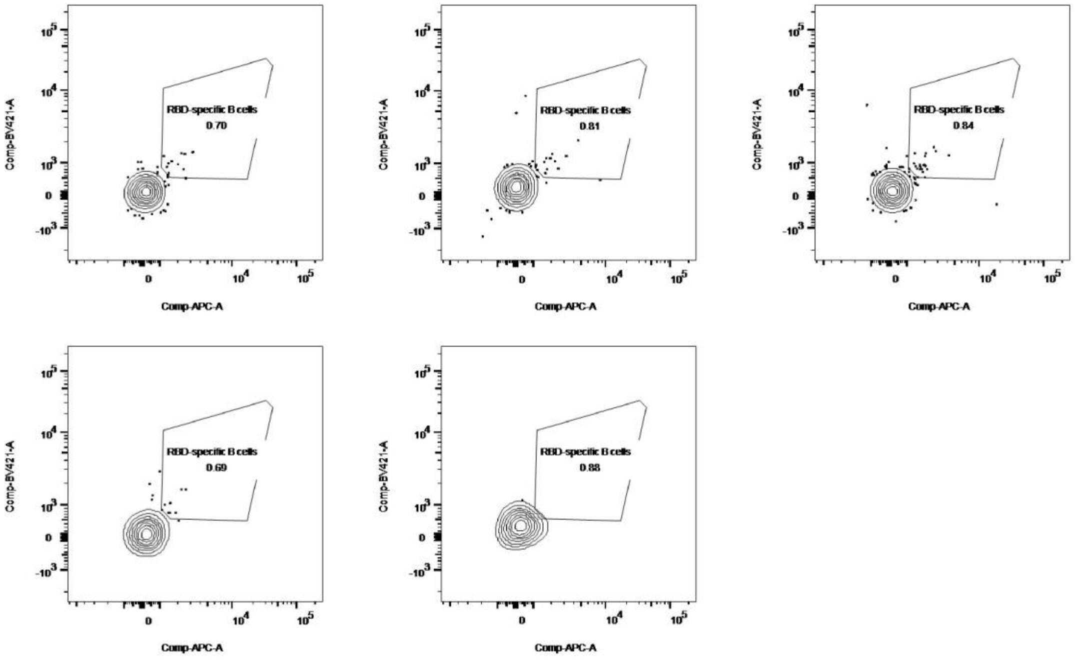

Supplementary Fig. 61 Gating analysis for the identification of $IgD^{-}IgM^{-}RBD^{+}$ B cells in spleens from the mice of pDLS3-LNP group.

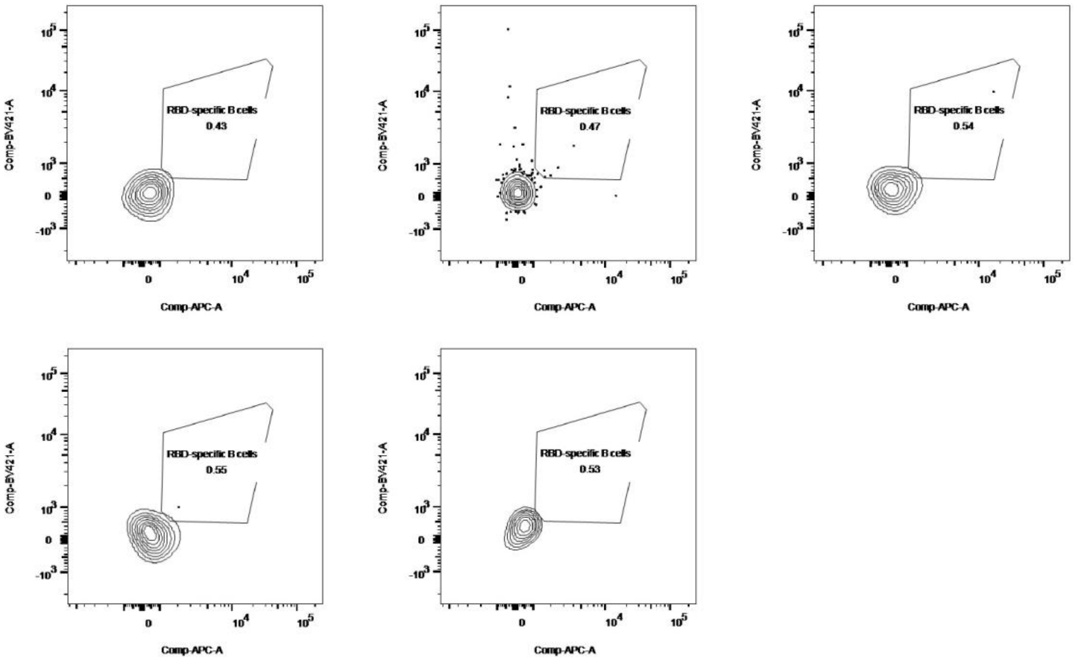

Supplementary Fig. 62 Gating analysis for the identification of $IgD^{-}IgM^{-}RBD^{+}$ B cells in spleens from the mice of pDLS4-LNP group.

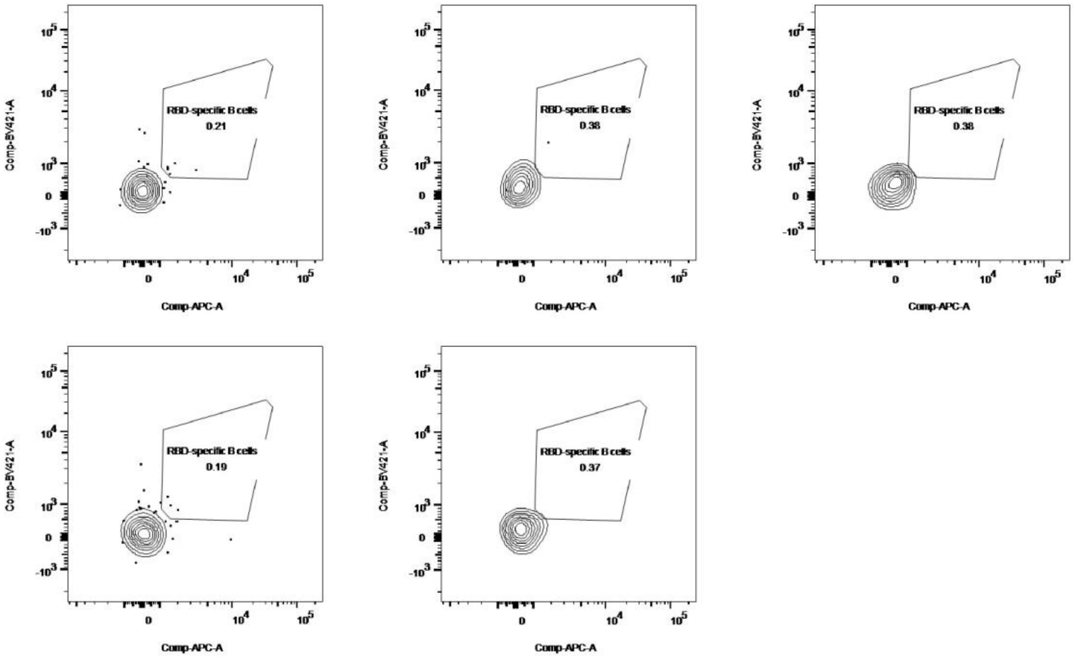

Supplementary Fig. 63 Gating analysis for the identification of IgD^{-}IgM^{-}RBD^{+} B cells in spleens from the mice of pDLS5-LNP group.

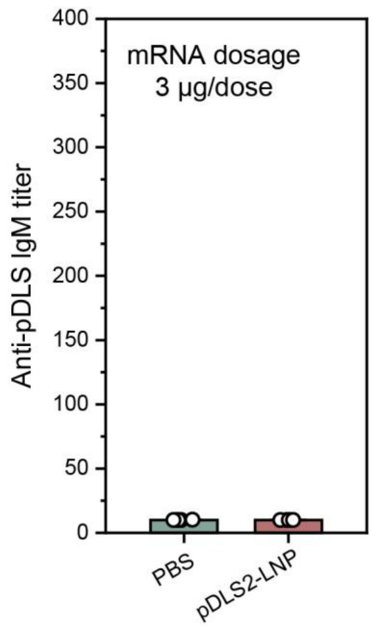

Supplementary Fig. 64 Anti-pDLS IgM titers in mice treated with SARS-CoV-2 mRNA-loaded pDLS2-LNP at week 4 (*n* = 5 independent biological samples; mean ± SD). Source data are provided as a Source Data file.

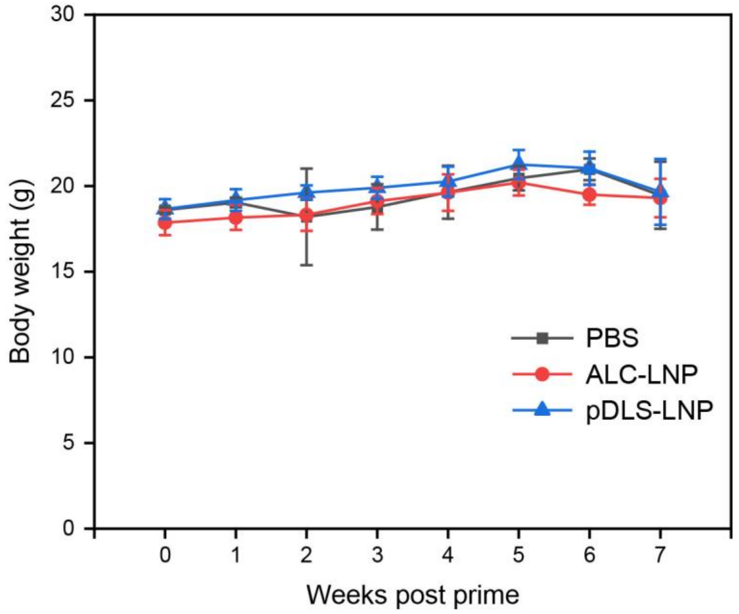

Supplementary Fig. 65 Body weight changes throughout the anti-PEG and anti-pDLS study. BALB/c mice were s.c. injected with FLuc mRNA-loaded ALC-LNP or pDLS1-LNP (1 µg mRNA per mouse) at week 0 and 3 (*n* = 5 independent biological samples; mean ± SD). Source data are provided as a Source Data file.

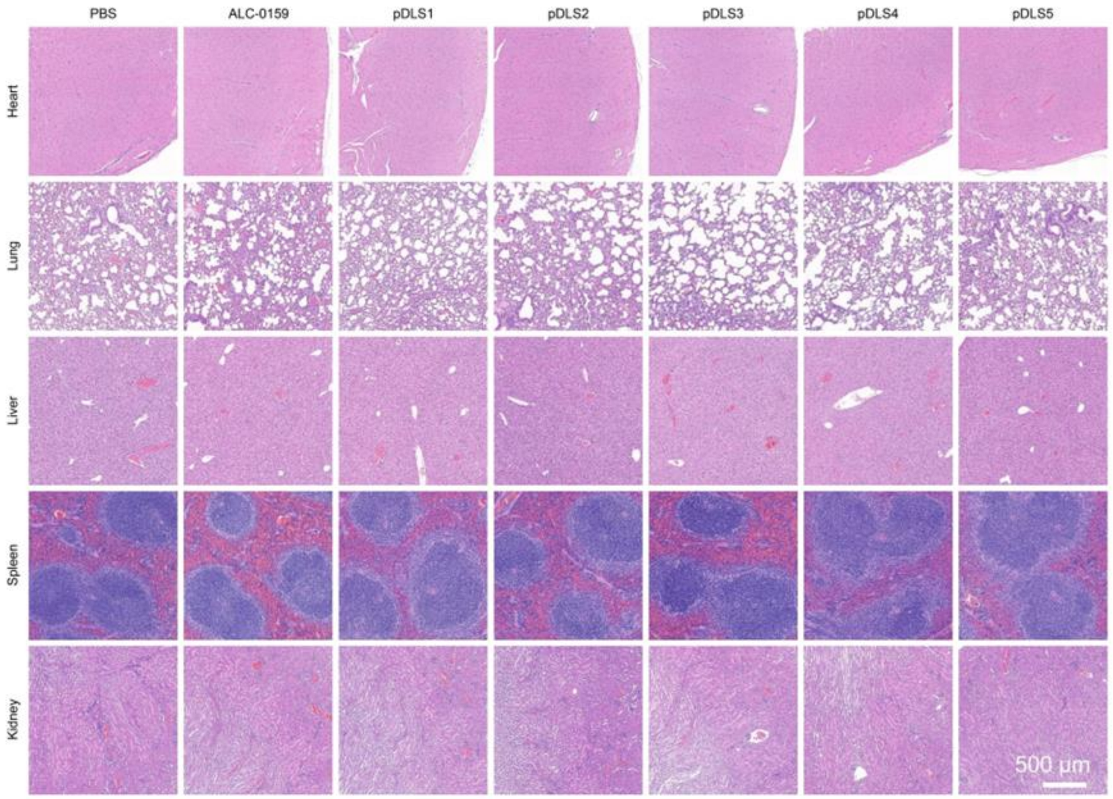

Supplementary Fig. 66 Representative H&E staining images of the heart, lung, liver, spleen, and kidney. BALB/c mice were s.c. injected with SARS-CoV-2 mRNA-loaded ALC- or pDLS-LNPs (3 μg mRNA) at week 0 and 3. The major organs were collected at the end of the experiment at week 11. Each experiment was repeated twice independently with similar results, and a representative result is shown for each.

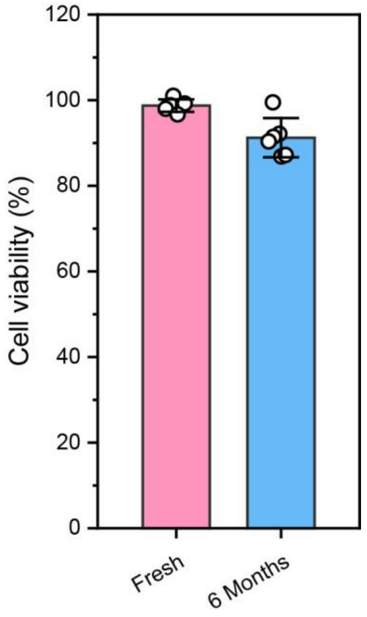

Supplementary Fig. 67 Viability of DC2.4 cells after treated with FLuc mRNA-loaded pDLS2-LNP prepared *via* microfluidic mixing following 6 months of storage at −80 °C. DC2.4 cells were treated with the LNPs at an mRNA dose of 100 ng per well in 96-well plates for 48 h (n = 6 independent biological samples; mean ± SD). Source data are provided as a Source Data file.

3. Supplementary Tables

Supplementary Table 1 Reactant feed ratios for synthesis of poly(o-benzyl-D, L-Serine) lipids.

Polypeptide lipid	Initiator Lipid (mmol)	Monomer 1 L-Ser-NCA (mmol)	Monomer 2 D-Ser-NCA (mmol)	Solvent DCM (mL)	Reaction Time (h)
pDLS1	0.1	1.5	1.5	40	48
pDLS2	0.1	2.0	2.0	40	48
pDLS3	0.1	2.5	2.5	40	48
pDLS4	0.1	3.0	3.0	40	48
pDLS5	0.1	4.0	4.0	40	48

Supplementary Table 2 Characteristics of poly(D, L-Serine) (pDLS) lipids.

Polypeptide lipid	Initiator Lipid	Degree of polymerization	M_n^{NMR} [g mol^{-1}]
pDLS1	2-amino-N,N-ditetradecylacetamide	18	2076
pDLS2	2-amino-N,N-ditetradecylacetamide	21	2338
pDLS3	2-amino-N,N-ditetradecylacetamide	27	2860
pDLS4	2-amino-N,N-ditetradecylacetamide	32	3295
pDLS5	2-amino-N,N-ditetradecylacetamide	45	4427
pDLS18D	2-amino-N,N-dioctadecylacetamide	25	2796
pDLS18S	Octadecan-1-amine	23	2313
pDLS14S	Tetradecan-1-amine	25	2430
pDLS8D	6-amino-N,N-dioctylhexanamide	30	3007

Supplementary Table 3 Mole ratio (content) of lipids used in the LNP formulations using PEGylated or pDLS lipids with different DPs.

Formulation	ALC-0315	DSPC	Cholesterol	pDLS	ALC-0159
ALC-0159-0.5	46.8	9.5	43.2	0	0.5
ALC-0159-1.0	46.6	9.4	43.0	0	1.0
ALC-0159-1.6	46.3	9.4	42.7	0	1.6
ALC-0159-2.5	45.9	9.3	42.3	0	2.5
ALC-0159-5.0	44.7	9.1	41.2	0	5.0
pDLS1-0.5	46.8	9.5	43.2	0.5	0
pDLS1-1.0	46.6	9.4	43.0	1.0	0
pDLS1-1.6	46.3	9.4	42.7	1.6	0
pDLS1-2.5	45.9	9.3	42.3	2.5	0
pDLS1-5.0	44.7	9.1	41.2	5.0	0
pDLS2-0.5	46.8	9.5	43.2	0.5	0
pDLS2-1.0	46.6	9.4	43.0	1.0	0
pDLS2-1.6	46.3	9.4	42.7	1.6	0
pDLS2-2.5	45.9	9.3	42.3	2.5	0
pDLS2-5.0	44.7	9.1	41.2	5.0	0
pDLS3-0.5	46.8	9.5	43.2	0.5	0
pDLS3-1.0	46.6	9.4	43.0	1.0	0
pDLS3-1.6	46.3	9.4	42.7	1.6	0
pDLS3-2.5	45.9	9.3	42.3	2.5	0
pDLS3-5.0	44.7	9.1	41.2	5.0	0
pDLS4-0.5	46.8	9.5	43.2	0.5	0
pDLS4-1.0	46.6	9.4	43.0	1.0	0
pDLS4-1.6	46.3	9.4	42.7	1.6	0
pDLS4-2.5	45.9	9.3	42.3	2.5	0
pDLS4-5.0	44.7	9.1	41.2	5.0	0
pDLS5-0.5	46.8	9.5	43.2	0.5	0
pDLS5-1.0	46.6	9.4	43.0	1.0	0
pDLS5-1.6	46.3	9.4	42.7	1.6	0
pDLS5-2.5	45.9	9.3	42.3	2.5	0
pDLS5-5.0	44.7	9.1	41.2	5.0	0

Supplementary Table 4 Mole ratio (content) of lipids used in the LNP formulations using pDLS lipids with different lipid structures.

Formulation	ALC-0315	DSPC	Cholesterol	pDLS	ALC-0159
pDLS18D-0.5	46.8	9.5	43.2	0.5	0
pDLS18D-1.0	46.6	9.4	43.0	1.0	0
pDLS18D-1.6	46.3	9.4	42.7	1.6	0
pDLS18D-2.5	45.9	9.3	42.3	2.5	0
pDLS18D-5.0	44.7	9.1	41.2	5.0	0
pDLS18S-0.5	46.8	9.5	43.2	0.5	0
pDLS18S-1.0	46.6	9.4	43.0	1.0	0
pDLS18S-1.6	46.3	9.4	42.7	1.6	0
pDLS18S-2.5	45.9	9.3	42.3	2.5	0
pDLS18S-5.0	44.7	9.1	41.2	5.0	0
pDLS14S-0.5	46.8	9.5	43.2	0.5	0
pDLS14S-1.0	46.6	9.4	43.0	1.0	0
pDLS14S-1.6	46.3	9.4	42.7	1.6	0
pDLS14S-2.5	45.9	9.3	42.3	2.5	0
pDLS14S-5.0	44.7	9.1	41.2	5.0	0
pDLS8D-0.5	46.8	9.5	43.2	0.5	0
pDLS8D-1.0	46.6	9.4	43.0	1.0	0
pDLS8D-1.6	46.3	9.4	42.7	1.6	0
pDLS8D-2.5	45.9	9.3	42.3	2.5	0
pDLS8D-5.0	44.7	9.1	41.2	5.0	0

Supplementary Table 5 Transfected luminescence intensity of DC2.4 cells treated with FLuc mRNA-loaded ALC-LNP and pDLS-LNPs (LNPs were formulated *via* pipette mixing).

Formulations	Sample 1	Sample 2	Sample 3
ALC-0159 MR1.6%	170589	152986	153387
pDLS1 MR0.5%	643581	620789	619160
pDLS1 MR1.0%	647722	567989	576997
pDLS1 MR1.6%	608389	576067	554148
pDLS1 MR2.5%	587097	549265	535957
pDLS1 MR5.0%	370850	363983	359153
pDLS2 MR0.5%	839925	790357	771050
pDLS2 MR1.6%	577643	599930	575733
pDLS2 MR2.5%	510176	463287	489866
pDLS2 MR5.0%	337684	356381	349563
ALC-0159 MR1.6%	288137	234905	250889
pDLS2 MR1.0%	989690	985467	874816
ALC-0159 MR1.6%	140594	151411	137835
pDLS3 MR0.5%	621082	602006	634961
pDLS3 MR1.6%	654476	630124	647125
pDLS3 MR2.5%	486333	545284	511653
pDLS3 MR5.0%	299525	305811	310234
ALC-0159 MR1.6%	118168	124550	118504
pDLS3 MR1.0%	666102	574569	603578
ALC-0159 MR1.6%	133670	126713	128227
pDLS4 MR0.5%	1018711	968327	951735
pDLS4 MR1.0%	1117528	1064928	1039048
pDLS4 MR1.6%	1392373	1289060	1290488
pDLS4 MR2.5%	1106672	973409	1028776
pDLS4 MR5.0%	314727	357406	294503
pDLS5 MR0.5%	1020998	1034941	1014886
pDLS5 MR1.0%	795930	842545	843999
pDLS5 MR1.6%	383196	339497	373423
pDLS5 MR2.5%	246499	234531	261872
pDLS5 MR5.0%	73996	75656	79625
ALC-0159 MR1.6%	283898	285931	284853
pDLS8D MR0.5%	1090350	1108033	1099538
pDLS8D MR1.0%	769143	783624	772930
pDLS8D MR1.6%	644378	612990	612815
pDLS8D MR2.5%	551739	568667	548372
pDLS8D MR5.0%	410908	421415	409109
pDLS18D MR0.5%	1317924	1303336	1293671
pDLS18D MR1.0%	1695464	1813910	1849760

pDLS18D MR1.6%	1237538	1301063	1334612
pDLS18D MR2.5%	1693431	1674341	1791956
pDLS18D MR5.0%	815846	788765	848441
ALC-0159 MR1.6%	513842	506362	468425
pDLS14S MR0.5%	1767962	1643204	1659588
pDLS14S MR1.0%	1526171	1400152	1393877
pDLS14S MR1.6%	1640874	1623648	1617711
pDLS14S MR2.5%	1341760	1419399	1409608
pDLS14S MR5.0%	761848	774491	815656
pDLS18S MR0.5%	1392033	1434606	1364244
pDLS18S MR1.0%	1481655	1514946	1519472
pDLS18S MR1.6%	1360599	1503332	1454789
pDLS18S MR2.5%	1563096	1646581	1538963
pDLS18S MR5.0%	1547961	1617235	1758193

Supplementary Table 6 Transfected luminescence intensity of HEK 293T cells treated with FLuc mRNA-loaded ALC-LNP and pDLS-LNPs (LNPs were formulated *via* pipette mixing).

Formulations	Sample 1	Sample 2	Sample 3
ALC-0159 MR1.6%	2198726	2129355	2110939
pDLS1 MR0.5%	3942194	3123921	3409061
pDLS1 MR1.0%	4197772	3618740	3787282
pDLS1 MR1.6%	5211428	4739630	5506757
pDLS1 MR2.5%	6798942	6317610	6765721
pDLS1 MR5.0%	8765089	8653182	7508502
pDLS2 MR0.5%	5915649	5520240	5239461
pDLS2 MR2.5%	4186747	4470243	4162684
pDLS2 MR5.0%	7693497	7180708	6543959
ALC-0159 MR1.6%	1772237	1568141	1378011
pDLS2 MR1.0%	3993773	4058768	3881786
pDLS2 MR1.6%	5172716	5847078	4647916
ALC-0159 MR1.6%	1293623	1328724	1229993
pDLS3 MR0.5%	4599909	3965205	3935396
pDLS3 MR1.6%	5632571	5996001	6290335
pDLS3 MR2.5%	6453933	6393969	6301764
pDLS3 MR5.0%	7897151	7895206	7787379
ALC-0159 MR1.6%	3165291	3031507	3129565
pDLS3 MR1.0%	9002212	8725494	8018797
ALC-0159 MR1.6%	239906	269542	248114
pDLS4 MR0.5%	818318	919244	909898
pDLS4 MR1.0%	1244587	1342157	1321501
pDLS4 MR1.6%	1705427	1409840	1810187
pDLS4 MR2.5%	1228310	1417158	1255544
pDLS4 MR5.0%	1172435	1421711	1348940
pDLS5 MR0.5%	768208	766907	802572
pDLS5 MR1.0%	891387	802097	868013
pDLS5 MR1.6%	614622	597036	678988
pDLS5 MR2.5%	312455	331988	314961
pDLS5 MR5.0%	95270	117348	80182
ALC-0159 MR1.6%	1318897	1291457	1266049
pDLS8D MR0.5%	1780534	1684899	1734832
pDLS8D MR1.0%	1701436	1702030	1679957
pDLS8D MR1.6%	1198328	1161066	1118172
pDLS8D MR2.5%	1390148	1391988	1339917
pDLS8D MR5.0%	1111458	1085055	1126699
pDLS18D MR0.5%	2825013	2558508	2535447
pDLS18D MR1.0%	4327305	4047353	3532058

pDLS18D MR1.6%	3605990	3452625	3156127
pDLS18D MR2.5%	5092852	4713777	4966508
pDLS18D MR5.0%	4639702	4537380	4299480
ALC-0159 MR1.6%	1326310	1360389	1286805
pDLS-14S MR0.5%	3545014	3282430	3290017
pDLS-14S MR1.0%	2237420	2059816	1946203
pDLS-14S MR1.6%	2917721	2500891	2529057
pDLS-14S MR2.5%	2310310	2283953	2318656
pDLS-14S MR5.0%	2303245	2103420	2209553
pDLS-18S MR0.5%	1693332	1581021	1592893
pDLS-18S MR1.0%	1873876	1724462	1693249
pDLS-18S MR1.6%	1441872	1300954	1041616
pDLS-18S MR2.5%	1282030	1271423	1226641
pDLS-18S MR5.0%	519617	351342	546638
