# Supplementary material for: Polypeptide-engineered lipid nanoparticles for mRNA delivery with limited immunogenicity
Source: Nat Commun. 2026 May 29;17:6986. doi: 10.1038/s41467-026-73698-6 (PMC13392015; doi:10.1038/s41467-026-73698-6)
Supplement: Supplementary file 2 — Reporting Summary [file 41467_2026_73698_MOESM2_ESM.pdf]

nature portfolio

Corresponding author(s): Yi Yan Yang

Last updated by author(s): Apr 25, 2026

Reporting Summary

Nature Portfolio wishes to improve the reproducibility of the work that we publish. This form provides structure for consistency and transparency in reporting. For further information on Nature Portfolio policies, see our Editorial Policies and the Editorial Policy Checklist.

Statistics

For all statistical analyses, confirm that the following items are present in the figure legend, table legend, main text, or Methods section.

n/a	Confirmed	
☐	☒	The exact sample size (*n*) for each experimental group/condition, given as a discrete number and unit of measurement
☐	☒	A statement on whether measurements were taken from distinct samples or whether the same sample was measured repeatedly
☐	☒	The statistical test(s) used AND whether they are one- or two-sided<br>*Only common tests should be described solely by name; describe more complex techniques in the Methods section.*
☒	☐	A description of all covariates tested
☐	☒	A description of any assumptions or corrections, such as tests of normality and adjustment for multiple comparisons
☐	☒	A full description of the statistical parameters including central tendency (e.g. means) or other basic estimates (e.g. regression coefficient) AND variation (e.g. standard deviation) or associated estimates of uncertainty (e.g. confidence intervals)
☒	☐	For null hypothesis testing, the test statistic (e.g. *F*, *t*, *r*) with confidence intervals, effect sizes, degrees of freedom and *P* value noted<br>*Give P values as exact values whenever suitable.*
☒	☐	For Bayesian analysis, information on the choice of priors and Markov chain Monte Carlo settings
☒	☐	For hierarchical and complex designs, identification of the appropriate level for tests and full reporting of outcomes
☐	☒	Estimates of effect sizes (e.g. Cohen's *d*, Pearson's *r*), indicating how they were calculated

Our web collection on statistics for biologists contains articles on many of the points above.

Software and code

Policy information about availability of computer code

Data collection	BD FACSDiva™ software, Mabtech Apex 1.1.9 software, Living Image software (PerkinElmer, USA)
Data analysis	All statistical analyses were performed on Graphpad Prism 10. Living Image software (Perkin Elmer) was used to analyze bioluminescent and fluorescent images. Image J (Version 1.48h3) was used for fluorescence-image analysis. FlowJo™ v10 was used to flow cytometry analysis.

For manuscripts utilizing custom algorithms or software that are central to the research but not yet described in published literature, software must be made available to editors and reviewers. We strongly encourage code deposition in a community repository (e.g. GitHub). See the Nature Portfolio guidelines for submitting code & software for further information.

Data

Policy information about availability of data

All manuscripts must include a data availability statement. This statement should provide the following information, where applicable:

- Accession codes, unique identifiers, or web links for publicly available datasets
- A description of any restrictions on data availability
- For clinical datasets or third party data, please ensure that the statement adheres to our policy

A data availability statement was provided.

Research involving human participants, their data, or biological material

Policy information about studies with human participants or human data. See also policy information about sex, gender (identity/presentation), and sexual orientation and race, ethnicity and racism.

Reporting on sex and gender	Not applicable.
Reporting on race, ethnicity, or other socially relevant groupings	Not applicable.
Population characteristics	Not applicable.
Recruitment	Not applicable.
Ethics oversight	Not applicable.

Note that full information on the approval of the study protocol must also be provided in the manuscript.

Field-specific reporting

Please select the one below that is the best fit for your research. If you are not sure, read the appropriate sections before making your selection.

☒ Life sciences ☐ Behavioural & social sciences ☐ Ecological, evolutionary & environmental sciences

For a reference copy of the document with all sections, see nature.com/documents/nr-reporting-summary-flat.pdf

Life sciences study design

All studies must disclose on these points even when the disclosure is negative.

Sample size	Sample sizes were determined based on previous experimental experience and published literature.
Data exclusions	No data were excluded.
Replication	All experimental findings could be reliably reproduced.
Randomization	All samples/organisms were randomly allocated into experimental groups.
Blinding	Blinding was applied to all animal experiments.

Reporting for specific materials, systems and methods

We require information from authors about some types of materials, experimental systems and methods used in many studies. Here, indicate whether each material, system or method listed is relevant to your study. If you are not sure if a list item applies to your research, read the appropriate section before selecting a response.

Materials & experimental systems

n/a	Involved in the study	
☐	☒	Antibodies
☐	☒	Eukaryotic cell lines
☒	☐	Palaeontology and archaeology
☐	☒	Animals and other organisms
☒	☐	Clinical data
☒	☐	Dual use research of concern
☒	☐	Plants

Methods

n/a	Involved in the study	
☒	☐	ChIP-seq
☐	☒	Flow cytometry
☒	☐	MRI-based neuroimaging

Antibodies

Antibodies used	The antibodies used in study were listed as follows: anti-mouse CD16/32 (BioLegend, Cat# 101301, clone 93, Lot# B460768, 1:100); BV785 anti-mouse B220 (BioLegend, Cat# 103246, clone RA3-6B2, Lot# B379487, 1:25); PE/Cy5 anti-mouse CD19 (BioLegend, Cat# 115510, clone 6D5, Lot# B409651, 1:50); PE/Dazzle594 anti-mouse IgM (BioLegend, Cat# 406530, clone RMM-1, Lot# B365242, 1:25); BV711 anti-mouse IgD (BioLegend, Cat# 405731, clone 11-26c.2a, Lot# B370139, 1:50); AF700 anti-mouse CD38 (BioLegend,

	Cat# 102742, clone 90, Lot# B380905, 1:100); PE/Cy7 anti-mouse GL7 (BioLegend, Cat# 144619, clone GL7, Lot# B376163, 1:50); BV650 anti-mouse CD80 (BioLegend, Cat# 104732, clone 16-10A1, Lot# B401937, 1:25); PE anti-mouse PD-L2 (BioLegend, Cat# 107205, clone TY25, Lot# B392057, 1:10).
Validation	All antibodies were obtained from commercial sources and have been validated by the manufacturers for the indicated applications. Each lot undergoes quality control testing by the supplier. Validation data are available on the manufacturers' websites as follows: anti-mouse CD16/32 (BioLegend, Cat# 101301, clone 93, Lot# B460768, 1:100) (https://www.biolegend.com/en-gb/products/purified-anti-mouse-cd16-32-antibody-190); BV785 anti-mouse B220 (BioLegend, Cat# 103246, clone RA3-6B2, Lot# B379487, 1:25) (https://www.biolegend.com/en-gb/products/brilliant-violet-785-anti-mouse-human-cd45r-b220-antibody-7960); PE/Cy5 anti-mouse CD19 (BioLegend, Cat# 115510, clone 6D5, Lot# B409651, 1:50); (https://www.biolegend.com/en-gb/products/pe-cyanine5-anti-mouse-cd19-antibody-1531); PE/Dazzle594 anti-mouse IgM (BioLegend, Cat# 406530, clone RMM-1, Lot# B365242, 1:25) (https://www.biolegend.com/en-gb/products/pe-dazzle-594-anti-mouse-igm-12509); BV711 anti-mouse IgD (BioLegend, Cat# 405731, clone 11-26c.2a, Lot# B370139, 1:50) (https://www.biolegend.com/en-gb/products/brilliant-violet-711-anti-mouse-igd-9572); AF700 anti-mouse CD38 (BioLegend, Cat# 102742, clone 90, Lot# B380905, 1:100) (https://www.biolegend.com/en-gb/products/alexa-fluor-700-anti-mouse-cd38-antibody-20599); PE/Cy7 anti-mouse GL7 (BioLegend, Cat# 144619, clone GL7, Lot# B376163, 1:50) (https://www.biolegend.com/en-gb/products/pe-cyanine7-anti-mouse-human-gl7-antigen-t-and-b-cell-activation-marker-antibody-17249); BV650 anti-mouse CD80 (BioLegend, Cat# 104732, clone 16-10A1, Lot# B401937, 1:25) (https://www.biolegend.com/en-gb/products/brilliant-violet-650-anti-mouse-cd80-antibody-7642); PE anti-mouse PD-L2 (BioLegend, Cat# 107205, clone TY25, Lot# B392057, 1:10) (https://www.biolegend.com/en-gb/products/pe-anti-mouse-cd273-b7-dc-pd-l2-antibody-2547).

Eukaryotic cell lines

Policy information about cell lines and Sex and Gender in Research

Cell line source(s)	The following cell lines were used in this work: The mouse dendritic cell line DC2.4 (Merck, Cat# SCC142M), the human embryonic kidney cell line HEK293T (ATCC, Cat# CRL-3216), and the HEK293-GFP stable cells (GeneTarget, Cat# SC001).
Authentication	Each cell line we used was authenticated by the original source and morphologically verified upon receipt according to the source specifications.
Mycoplasma contamination	All cell lines were tested for mycoplasma contamination. No mycoplasma contamination was found.
Commonly misidentified lines (See ICLAC register)	No commonly misidentified cell lines were used.

Animals and other research organisms

Policy information about studies involving animals; ARRIVE guidelines recommended for reporting animal research, and Sex and Gender in Research

Laboratory animals	BALB/c mice (6-8 weeks old, female) were used in this study and purchased from InVivos Pte Ltd.
Wild animals	The study did not involve wild animals studies.
Reporting on sex	Female BALB/c mice were used in this study.
Field-collected samples	The study did not involve filed-collection samples.
Ethics oversight	All animals were maintained in a specific-pathogen-free facility and received care in compliance with the Guidelines for Care and Use of Laboratory Animals. The facility has a 12-hour light/dark cycle, with a standard environmental temperature of 20-24 °C and humidity of 40-60%. All animal procedures were performed per the approved protocol from the Institutional Animal Care and Use Committee (IACUC) at the Biological Resource Centre of A*STAR, Singapore (IACUC protocol number: 221681 and its renewed approval: 251905.

Note that full information on the approval of the study protocol must also be provided in the manuscript.

Plants

Seed stocks	Not applicable.
Novel plant genotypes	Not applicable.
Authentication	Not applicable.

Flow Cytometry

Plots

Confirm that:

☒ The axis labels state the marker and fluorochrome used (e.g. CD4-FITC).

☒ The axis scales are clearly visible. Include numbers along axes only for bottom left plot of group (a 'group' is an analysis of identical markers).

☒ All plots are contour plots with outliers or pseudocolor plots.

☒ A numerical value for number of cells or percentage (with statistics) is provided.

Methodology

Sample preparation	Spleens were collected and dissociated into single-cell suspensions using RPMI 1640 medium. The suspensions were filtered through 70 µm cell strainers, and centrifuged at 1000 g for 5 min at 4 °C. Red blood cells were subsequently lysed by treating the suspensions with ACK Lysing Buffer. single-cell suspensions were obtained and stained with antibodies according to the manufacturer's protocols, and then analyzed by flow cytometry.
Instrument	BD FACSymphony A3
Software	FlowJo v10
Cell population abundance	At least 500,000 cells were collected and analyzed for fluorescence intensity within the defined gate.
Gating strategy	Cells were initially gated based on FSC/SSC, followed by singlet discrimination using FSC-H versus FSC-A. Subsequent gating for surface and intracellular antigens was performed as described in the Supplementary Information.

☒ Tick this box to confirm that a figure exemplifying the gating strategy is provided in the Supplementary Information.
