# Supplementary material for: Polypeptide-engineered lipid nanoparticles for mRNA delivery with limited immunogenicity
Source: Nat Commun. 2026 May 29;17:6986. doi: 10.1038/s41467-026-73698-6 (PMC13392015; doi:10.1038/s41467-026-73698-6)
Supplement: Supplementary file 3 — Transparent Peer Review file [file 41467_2026_73698_MOESM3_ESM.pdf]

nature portfolio

Peer Review File

Polypeptide-engineered lipid nanoparticles for mRNA delivery with limited immunogenicity

Corresponding Author: Dr Yi Yan Yang

This file contains all reviewer reports in order by version, followed by all author rebuttals in order by version.

Version 0:

Reviewer comments:

Reviewer #2

(Remarks to the Author)
The presented manuscript, “Polypeptide-engineered lipid nanoparticles achieve safe and efficacious mRNA delivery with low immunogenicity”, is of significant interest and relevance to researchers in the fields of drug delivery and nanomedicine. It provides a detailed investigation into how polypeptides can replace PEG in LNPs, reducing the induction of anti-PEG antibodies and thereby enhancing therapeutic safety and enabling more efficient redosing. The authors have conducted an extensive screening of various LNP formulations incorporating different pDLS variants as PEG alternatives. A comprehensive set of assays was employed to quantify cellular uptake, endosomal escape, and gene expression, offering valuable mechanistic insights.
My main concern relates to the lack of appropriate control samples, particularly in the in vitro experiments. Including such controls would strengthen the reliability of the presented data and conclusions. Furthermore, it is not always clear from the supporting information which characterization data correspond to which set of experiments. Additionally, while the manuscript primarily focuses on mRNA delivery, the inclusion of other therapeutic payloads—such as siRNA—could broaden the scope and impact of the study. Overall, the manuscript addresses a timely and important topic and contributes meaningful findings to the field. With the inclusion of the aforementioned controls and consideration of additional payloads, the work would be significantly strengthened.
Before publication I would recommend following revisions:

1) The publication highlights PEG replacement in the context of mRNA delivery. To broaden the impact, it would be beneficial to include a cell-based experiment (or even mice studies) demonstrating the efficient delivery of a different type of cargo, such as siRNA, sgRNA, or even Cas9-sgRNA ribonucleoprotein (RNP) complexes.

2) In the Abstract, it is stated:” …with polyethylene glycol (PEG) lipids playing a critical role in modulating colloidal stability, biodistribution, pharmacokinetics, and storage performance.” This is a good summary of the role of PEG lipids, but it should also be mentioned that PEG contributes to controlling the size of the resulting LNPs.

3) The authors write:”…cholesterol and mRNA yields colloidally stable PEG-free pDLS-LNPs with good size distribution, near-neutral zeta potential and comparable mRNA encapsulation efficiency (Fig. 1c 1d.). I assume the authors meant to refer to Figure 2 instead?

4) The authors mention that “random copolymerization” was used Is there any data available on batch-to-batch variation?

5) The authors write:”…which features a double C14 alkyl chain, consistently demonstrated higher encapsulation efficiencies across all tested molar ratios compared to LNPs formulated with other lipid tail architectures…” For clarity and ease of reading, please include the actual encapsulation efficiency range in the text.

6) In figure 2a the luminescence of the reference sample is not shown. Including this is essential to allow the reader to draw validated conclusions from the experiment.

7) The in vitro LNPs were formulated via pipette mixing, whereas the samples for in vivo studies were prepared using microfluidic mixing. How does this difference affect the results? Could you show that the formulating method doesn’t matter for your outcome? Since recent studies show that there are differences between pipette mixing and microfluidic mixing. See:

The mixing method used to formulate lipid nanoparticles affects mRNA delivery efficacy and organ tropism - ScienceDirect

8) Furthermore, do you have cryo-EM images of the LNPs used for the in vivo studies? The internal structure appears unstructured in the LNPs formulated via pipette mixing, which may not be the case for LNPs prepared by microfluidic mixing. Including these images would help clarify structural differences.

9) Supplementary Figures 22–35 are missing data for the control samples. Since the focus of the paper is on PEG replacement, including the reference (PEG-containing) samples is essential to allow proper comparison and interpretation.

10) Supplementary Figures 26 and 27 do not specify which mRNA was encapsulated. This information is important, as Fluc mRNA is significantly larger in size and therefore more difficult to encapsulate. Additionally, please indicate whether these LNPs were prepared via pipette mixing or microfluidic mixing.

11) In the experimental section describing cellular uptake, it is stated:" On the next day, the cells were exposed to LNPs containing Cy5-labeled mRNA at a dose of 1000 ng." This is inconsistent with the cytotoxicity study, which was performed using a 100 ng dose. I recommend either repeating the in vitro study to assess uptake and protein expression at 100 ng, or performing a cytotoxicity assay at 1000 ng to ensure consistency and correlation.

12) Regarding the hemolysis assay, how does the lipid composition of the red blood cell (RBC) membrane compare to that of endosomal membranes? Adding one sentence comparison both would help to interpret the physiological relevance of the assay.

13) How were the LNPs dosed for the ex vivo biodistribution experiment shown in Figure 5? Was the injected dose normalized to the body weight of the mice?

14) I understand that the scope of the paper is vaccination related and that's the reason for the s.c. administration route. However, do you have any data on i.V. administration and if it changes biodistribution?

Reviewer #4

(Remarks to the Author)
I co-reviewed this manuscript with one of the reviewers who provided the listed reports. This is part of the Nature Communications initiative to facilitate training in peer review and to provide appropriate recognition for Early Career Researchers who co-review manuscripts.

Version 1:

Reviewer comments:

Reviewer #2

(Remarks to the Author)
Dear Editor,
I have reviewed the revised version of this manuscript and the accompanying responses to my comments.
The authors have addressed very detailed all of my previous comments. The revisions have substantially improved the clarity, methodological transparency, and overall quality of the manuscript. I appreciate the responses provided in the rebuttal letter, and I am satisfied that the concerns raised during the initial review have been adequately resolved.
In its current form, I believe the manuscript is suitable for publication and recommend acceptance.

Reviewer #4

(Remarks to the Author)
I co-reviewed this manuscript with one of the reviewers who provided the listed reports. This is part of the Nature Communications initiative to facilitate training in peer review and to provide appropriate recognition for Early Career Researchers who co-review manuscripts.

Reviewer #5

(Remarks to the Author)
I co-reviewed this manuscript with one of the reviewers who provided the listed reports. This is part of the Nature Communications initiative to facilitate training in peer review and to provide appropriate recognition for Early Career Researchers who co-review manuscripts.

Furthermore, my personal comments are as follows;

Main comments;

The authors developed mRNA-LNP using biodegradable poly(D, L-serine) (pDLS) lipids as PEG-lipid alternatives. It should be mentioned that there are several mRNA-LNP, which have been developed using other hydrophilic lipid instead of PEGylated lipid (Polym Rev, 63(2) (2023) 394-436, DOI; 10.1080/15583724.2022.2106490)

Most researchers prepared mRNA-LNPs containing 1.0–2.5 mol% PEGylated lipid, which suppressed LNP aggregation and led to the control of a homogeneous particle size distribution between 50 and 180 nm. When the fraction of PEGylated lipid increases, the inflammatory secretion of cytokines is reduced. However, the expression of specific proteins from mRNA transcription was also found to decrease (Mol. Ther. Nucleic Acids. 2014, 3, e210. DOI: 10.1038/mtna.2014.61). Therefore, several PEG alternatives have been developed to avoid the disadvantage of using PEGylated nanoparticles, such as sugar-based molecules, polypeptides, polyoxazolines, and polyglycerols (ACS Appl. Nano Mater. 2020, 3, 10634–10645. DOI: 10.1021/acsanm.0c01834.; Angew. Chem. Int. Ed. Engl. 2010, 49, 6288–6308. DOI: 10.1002/anie.200902672; Polym Rev, 63(2) (2023) 394-436, DOI: 10.1080/15583724.2022.2106490). Polysarcosine is a polypeptoid based on an endogenous amino acid (N-methylated glycine) of sarcosine, which is reported to have potent stealth properties (Chem. Rev. 2016, 116, 1753–1802. DOI: 10.1021/acs.chemrev.5b00201; Macromol. Biosci. 2015, 15, 881–891. DOI: 10.1002/mabi.201500023; ACS Nano. 2018, 12, 8646–8661. DOI: 10.1021/acsnano.8b04433). Feneroli et al. reported that polysarcosine lipids do not show an acute immune response in mice and zebrafish embryos (ACS Nano. 2018, 12, 8646–8661. DOI: 10.1021/acsnano.8b04433.). Therefore, Nogueira et al. designed polysarcosine-conjugated lipids with different lengths (ACS Appl. Nano Mater. 2020, 3, 10634–10645. DOI: 10.1021/acsanm.0c01834). mRNA-LNPs prepared with the polysarcosine-conjugated lipid with different polymeric chain lengths and molar fractions enabled the control of the physicochemical characteristics of the mRNA-LNPs, such as their internal structure, morphology and particle size. mRNA-LNPs prepared with the polysarcosine-conjugated lipid with the optimal length showed decreased cytokine inductions with higher specific protein production by mRNA compared with those prepared with DMG-PEG2000 (ACS Appl. Nano Mater. 2020, 3, 10634–10645. DOI: 10.1021/acsanm.0c01834). This study indicated that not only PEGylated lipids but also the development of other types of hydrophilic lipids, such as polysarcosine-conjugated lipids with an appropriate length (polymerization number (n)=23 in this study), are important for the efficient development of mRNA-LNPs (Polym Rev, 63(2) (2023) 394-436, DOI: 10.1080/15583724.2022.2106490).

As we can see most of researchers used a polysarcosine-conjugated lipid instead of PEGylated lipid, whereas the authors in this study used poly(D, L-serine) (pDLS)-conjugated lipid instead of PEGylated lipid. The authors should show or explain the merit of the usage of pDLS-conjugated lipids instead of polysarcosine-conjugated lipids.

The merit of polysarcosine-conjugated lipids is that polysarcosine is not a natural component, which would has slow biodegradable speed in vivo, whereas L-serine site on poly(D, L-serine) (pDLS)-conjugated lipid is expected to be degradated in vivo very fast, probably around 10 min. That means the mRNA-LNP developed in this study pDLS site will be immediately digested (disappeared) in treatment in vivo. Although in vitro results showed good using (pDLS)-conjugated lipid, in vivo study should show similar results showing control without PEGlated lipid. One suggestion from the reviewer is that poly(D-serine)-conjugated lipid would be valuable to generate mRNA-LNP for the author's project goal, because poly(D-serine)-conjugated lipid will not be degradated immediately in vivo environment.

Another comments

1. mRNA-LNP using pDLS usage showed negative zeta potential compared to mRNA-LNP using PEGylated lipid (Fig. 2i). When mRNA-LNP using polysarcosine-lipid, zeta potential will not show negative value because of chemical structure of polysarcosine.
2. In Fig 2 caption; "LNPs were prepared via pipette mixing" should be revised as "mRNA-LNPs were prepared via pipette mixing".
3. In Fig. 5, 6 and 8, the results of mRNA-pDLS-LNPs are almost the same to mRNA-PEGylated-LNPs after i.v. injection in vivo. Then why pDLS2-LNP or other pDLS-LNPs encapsulated with SARS-CoV-2 mRNA vaccines could elicit significantly higher robust or durable cellular and humoral immune responses in mice than ALC-LNP in Fig. 7?
4. In the description of the ex vivo bioluminescence images (panels j–o), it is unclear whether there is an additional panel following "o." If so, the corresponding sample name is missing. If not, the labeling should be corrected. This needs clarification.
5. In Fig. 5, only three formulations (PEGylated ALC-LNP, pDLS2-LNP, and pDLS5-LNP) were evaluated following intravenous administration. The rationale for selecting these specific candidates is not provided. The selection criteria must be clearly stated. Furthermore, the inclusion of tail-vein administration appears disconnected from the primary focus on subcutaneous delivery. The authors should explicitly justify the purpose of the i.v. study and clarify how it strengthens the overall conclusions. As presented, its placement disrupts the logical flow of the manuscript.
6. For safety assessment, the authors selected a pDLS-LNP formulated with a lipid of DP 23, described as being close to pDLS2. The rationale for this choice is insufficiently justified. If DP 23 is merely structurally similar to pDLS2, the added value of introducing this additional formulation is unclear. The authors must provide a clear, data-driven justification for selecting this candidate for safety evaluation.
7. The fluorescence distribution pattern shown in Fig. 8i appears markedly different from that presented in Fig. 4. The anatomical localization and signal distribution do not seem consistent between the two figures. Please clarify whether the animals in Fig. 8i and Fig. 4 were treated using the same injection route (e.g., s.c. vs i.v.), injection site, dose, and imaging time point. If the administration conditions differed, this must be clearly stated. If the conditions were intended to be identical, the discrepancy in biodistribution requires explanation.

Reviewer #6

(Remarks to the Author)
I co-reviewed this manuscript with one of the reviewers who provided the listed reports. This is part of the Nature Communications initiative to facilitate training in peer review and to provide appropriate recognition for Early Career Researchers who co-review manuscripts.

Reviewer #7

(Remarks to the Author)
Summary:
This study reports the development of polypeptide-based lipid nanoparticles (pDLS-LNPs) as alternatives to PEGylated LNPs for mRNA delivery. The authors introduce biodegradable, nonionic poly(D, L-serine) lipids to replace PEG, aiming to reduce anti-PEG antibody formation and improve redosing potential. Through systematic structural screening, optimized pDLS formulations achieved stable nanoparticles with high mRNA encapsulation and strong transfection efficiency. Compared with the clinically used ALC-LNP formulation, pDLS-LNPs demonstrated enhanced in vitro delivery and robust immune responses in vivo when delivering SARS-CoV-2 spike mRNA, without detectable systemic toxicity. Notably, repeated dosing induced minimal anti-pDLS IgM production, and the lead formulation maintained stability under frozen storage for over six months. Overall, the work positions polypeptide-engineered LNPs as a potential alternative platform for safer and effective mRNA delivery.
Though the study is robust with both in vitro and in vivo experimentation, there are certain concerns that need to be addressed by the authors before it is publishable in Nature Communications.
Major Concern
1. In the current version of the manuscript, the timeline for repeated dose testing is twice, which is minimal, and generally acceptable is at least 3, if not 4. While I understand that rerunning experiments at this point is resource constrained, I suggest providing a rationale and supporting it with additional research on studies that have been completed with a similar repeat dosage regime.

2. Clarification on the Pipette mixing versus the microfluidic mixing
The manuscript currently uses pipette mixing and microfluidic mixing approaches interchangeably across multiple sections, which significantly dilutes the impact and clarity of the work. It is harder for the reader to connect the result to those obtained with pipette mixing versus those obtained with microfluidic mixing. The absence of a clear rationale for switching between preparation methods makes it difficult for the reader to understand why a single approach was not consistently used. While I understand the possible reason for the authors is due to facile sample preparation and quick initial testing, these have to be mentioned in the manuscript to give the same context for the readers as well. If not in the main text, consider including that in the SI text.
3. Formulation Rationale:
Is there a specific rationale for selecting ALC-0315 and not SM-102 as the comparator lipid? Given that SM-102 is widely used in clinically validated mRNA-LNP systems, the choice of ALC should be justified explicitly. So, in the future follow-up works can also bank on such an explanation.
4. Figure-Specific Comments
Figure 2i:
Both sets of error bars should be clearly included in the figure. The variability appears substantial, particularly in the zeta potential measurements. Notably, pDLS1 and pDLS5 exhibit considerable fluctuation in zeta potential. The authors should address the implications of this variability, explain why it was observed in the tested samples, and clarify how variability in surface charge may affect stability, biodistribution, or biological interpretation.
Figure 2k:
The manuscript states “Comparable structural characteristics…”. However, one formulation (ALC) is described as more amorphous, while the pDLS particles appear at least twice as large as ALC-LNPs. Despite the use of microfluidic mixing, particle sizes remain substantially larger than in the control. The statement should therefore be moderated to reflect these structural differences. In addition, the authors should provide a rationale for the larger particle sizes observed in the pDLS formulations. It would also be important to clarify whether the in vitro testing showed variable transfection due to the larger size. Furthermore, the lack of size convergence despite microfluidic mixing requires explanation, and possible rationales should be included.
Figure 2c–2h (DLS Analysis):
The manuscript states “Narrow size distributions (PDI < 0.14).” While a PDI < 0.2 is generally considered acceptable, a value of 0.14 does not necessarily indicate a narrow monodisperse system and may still represent a mixed population. The authors should moderate this claim, provide additional supporting data, such as number-weighted versus intensity-weighted distributions, and clarify how “narrow” is defined in this context.
Figure 3 – Erythrocyte Model Concerns:
The use of erythrocytes to demonstrate delivery and cytotoxicity raises several questions. First, regarding biological context, it is unclear whether the erythrocytes were characterized for maturity or subtype and whether any assay was performed to confirm uniformity.
a. The rationale for choosing erythrocytes as the target cell type should also be clarified.
b. Second, mature erythrocytes lack nuclei, so it is unclear how Hoechst staining was observed and whether this is consistent with the cell type used. This requires clarification in the Materials and Methods section, particularly if there is a

specific explanation.
c. Third, fluorescence localization alone does not confirm endosomal escape. To substantiate this claim, 3D confocal reconstruction and colocalization with endosomal markers would be required. The current imaging appears insufficient to support spatial escape claims.
d. Finally, the plot profile analysis based on a single line scan is not representative of overall intensity, can introduce user bias, and does not provide quantitative robustness. Intensity quantification across multiple independent images should be performed, replicate analysis should be included, statistical treatment should be provided, and overinterpretation based on line-scan profiles should be avoided.
Minor comments:
5. Citation concerns on PEG Immunogenicity Discussion (Page 3)
a. The statement, “The high prevalence of PEG-specific antibodies in the general population has raised significant safety concerns…”, is currently supported by a single citation that is not directly relevant to mRNA vaccines. While PEG immunogenicity is an important topic, the manuscript appears to overstate the prevalence issue, base a strong claim on limited and non-specific evidence, and rely on review articles rather than original research. In particular, References 25 and 26 are review articles rather than primary research, and the cited article supporting the claim of “high prevalence” does not directly relate to mRNA-LNP vaccines. Although there is a broader trend in RNA therapeutics toward exploring PEG alternatives, the clinical implications have not yet been conclusively established, and instances of immunogenic outcomes are generally associated with repeated use.

6. It is premature to conclude that the storage conditions have been fully optimized without a systematic evaluation of excipients and comprehensive time-resolved stability studies. Therefore, the authors should moderate their claims regarding long-term stability at –80 °C and avoid implying that the formulation is fully optimized based on the current data.

Reviewer #8

(Remarks to the Author)
I co-reviewed this manuscript with one of the reviewers who provided the listed reports. This is part of the Nature Communications initiative to facilitate training in peer review and to provide appropriate recognition for Early Career Researchers who co-review manuscripts.

Version 2:

Reviewer comments:

Reviewer #5

(Remarks to the Author)
The authors revised the manuscript following reviewer's comments. Now the manuscript is acceptable for the publication in Nature Communications.

Reviewer #6

(Remarks to the Author)
I co-reviewed this manuscript with one of the reviewers who provided the listed reports. This is part of the Nature Communications initiative to facilitate training in peer review and to provide appropriate recognition for Early Career Researchers who co-review manuscripts.

Reviewer #7

(Remarks to the Author)
The authors have addressed all our comments, and the manuscript can be published in its current, latest submitted form.

Reviewer #8

(Remarks to the Author)
I co-reviewed this manuscript with one of the reviewers who provided the listed reports. This is part of the Nature Communications initiative to facilitate training in peer review and to provide appropriate recognition for Early Career Researchers who co-review manuscripts.

Open Access This Peer Review File is licensed under a Creative Commons Attribution 4.0 International License, which permits use, sharing, adaptation, distribution and reproduction in any medium or format, as long as you give appropriate credit to the original author(s) and the source, provide a link to the Creative Commons license, and indicate if changes were made.
In cases where reviewers are anonymous, credit should be given to 'Anonymous Referee' and the source.
The images or other third party material in this Peer Review File are included in the article's Creative Commons license, unless indicated otherwise in a credit line to the material. If material is not included in the article's Creative Commons license and your intended use is not permitted by statutory regulation or exceeds the permitted use, you will need to obtain permission directly from the copyright holder.
To view a copy of this license, visit https://creativecommons.org/licenses/by/4.0/

Responses to the reviewer's comments:

Reviewer #1:

The presented manuscript, "Polypeptide-engineered lipid nanoparticles achieve safe and efficacious mRNA delivery with low immunogenicity", is of significant interest and relevance to researchers in the fields of drug delivery and nanomedicine. It provides a detailed investigation into how polypeptides can replace PEG in LNPs, reducing the induction of anti-PEG antibodies and thereby enhancing therapeutic safety and enabling more efficient redosing. The authors have conducted an extensive screening of various LNP formulations incorporating different pDLS variants as PEG alternatives. A comprehensive set of assays was employed to quantify cellular uptake, endosomal escape, and gene expression, offering valuable mechanistic insights.

My main concern relates to the lack of appropriate control samples, particularly in the in vitro experiments. Including such controls would strengthen the reliability of the presented data and conclusions. Furthermore, it is not always clear from the supporting information which characterization data correspond to which set of experiments. Additionally, while the manuscript primarily focuses on mRNA delivery, the inclusion of other therapeutic payloads—such as siRNA—could broaden the scope and impact of the study. Overall, the manuscript addresses a timely and important topic and contributes meaningful findings to the field. With the inclusion of the aforementioned controls and consideration of additional payloads, the work would be significantly strengthened.

Before publication I would recommend following revisions:

Response: We thank the reviewer for the constructive comments. The manuscript has been revised according to the comments.

1) The publication highlights PEG replacement in the context of mRNA delivery. To broaden the impact, it would be beneficial to include a cell-based experiment (or even mice studies) demonstrating the efficient delivery of a different type of cargo, such as siRNA, sgRNA, or even Cas9-sgRNA ribonucleoprotein (RNP) complexes.

Response: Following the reviewer's suggestion, we explored polypeptide-engineered LNPs (pDLS-LNPs) for small interfering RNA (siRNA) delivery to broaden the scope and impact of the study. siRNA for silencing green fluorescent protein (GFP) (siGFP) was loaded into LNPs via pipette mixing. It's found that all formulations including ALC-LNP, pDLS2-LNP and pDLS5-LNP, showed nanosizes (< 200 nm), low polydispersity indices (PDI < 0.2), and near-neutral zeta potentials (within ±10 mV). Notably, both pDLS2-LNP and pDLS5-LNP exhibited much higher siGFP encapsulation efficiency compared to ALC-LNP (**Supplementary Fig. 42**). To evaluate GFP gene knockdown efficiency, we used flow cytometry to study LNP-mediated gene knockdown in GFP-expressing HEK293 (HEK293-GFP) cells. HEK293-GFP cells were incubated with LNPs containing siGFP for 48 h. siGFP-loaded LNPs formulated with pDLS2 or pDLS5 achieved significantly lower GFP expression compared to

PEGylated ALC-LNP, suggesting more effective siRNA delivery for gene silence using pDLS-LNPs (**Supplementary Fig. 43a**). siGFP-loaded LNPs were not toxic to HEK293-GFP cells (**Supplementary Fig. 43b**). Additional discussion has been added in the revised manuscript.

2) In the Abstract, it is stated:" …with polyethylene glycol (PEG) lipids playing a critical role in modulating colloidal stability, biodistribution, pharmacokinetics, and storage performance." This is a good summary of the role of PEG lipids, but it should also be mentioned that PEG contributes to controlling the size of the resulting LNPs.

Response: Following the reviewer's suggestion, an amendment has been added to the sentence in the abstract of the revised manuscript.

3) The authors write:"…cholesterol and mRNA yields colloidally stable PEG-free pDLS-LNPs with good size distribution, near-neutral zeta potential and comparable mRNA encapsulation efficiency (Fig. 1c 1d.). I assume the authors meant to refer to Figure 2 instead?

Response: We thank the reviewer for pointing this out. An amendment has been made accordingly in the revised manuscript.

4) The authors mention that "random copolymerization" was used. Is there any data available on batch-to-batch variation?

Response: Following the reviewer's suggestion, we evaluated the physicochemical properties and mRNA delivery efficiency of LNPs formulated with different synthetic batches of pDLS lipid. Taking pDLS2 as a representative example, pDLS2 lipid from two different batches exhibited a closely matched degree of polymerization (DP), as verified by ^{1}H NMR, with a batch-to-batch variation of 3 units (**Supplementary Fig. 22**). In addition, FLuc mRNA-loaded LNPs formulated with different batches of pDLS2 exhibited similar physicochemical properties and comparable mRNA encapsulation efficiencies (**Supplementary Fig. 37**). Moreover, FLuc mRNA-loaded LNPs formulated with pDLS2 from batch 1 or batch 2 mediated comparable mRNA transfection efficiency with cytocompatibility in both HEK 293T and DC2.4 cells (**Supplementary Fig. 38**). Additional discussion has been added in the revised manuscript.

5) The authors write:"…which features a double C14 alkyl chain, consistently demonstrated higher encapsulation efficiencies across all tested molar ratios compared to LNPs formulated with other lipid tail architectures…" For clarity and ease of reading, please include the actual encapsulation efficiency range in the text.

Response: Following the reviewer's suggestion, we have added the encapsulation efficiency range in the revised manuscript. "LNPs incorporating pDLS14D (pDLS3),

which features a double C14 alkyl chain, consistently demonstrated higher encapsulation efficiencies ranging from 73% to 84% across all tested molar ratios compared to LNPs formulated with other lipid tail architectures (**Supplementary Fig. 28**).”

6) In figure 2a the luminescence of the reference sample is not shown. Including this is essential to allow the reader to draw validated conclusions from the experiment.

Response: Fig. 2a and Fig. 2b showed the fold change in luminescence intensity mediated by Fluc mRNA-loaded pDLS-LNPs relative to that mediated by the PEGylated ALC-LNP control. Indeed, including raw luminescence data is essential to allow the reader to draw validated conclusions from the experiment. Following the reviewer’s suggestion, we have added the raw luminescence intensity data from FLuc mRNA-loaded pDLS-LNPs, and PEGylated ALC-LNP (N/P ratio, 6) control in the revised manuscript (**Supplementary Tables 5** and **6**).

7) The in vitro LNPs were formulated via pipette mixing, whereas the samples for in vivo studies were prepared using microfluidic mixing. How does this difference affect the results? Could you show that the formulating method doesn’t matter for your outcome? Since recent studies show that there are differences between pipette mixing and microfluidic mixing. See: The mixing method used to formulate lipid nanoparticles affects mRNA delivery efficacy and organ tropism – ScienceDirect

Response: Indeed, the mixing method used to formulate lipid nanoparticles affects mRNA delivery efficacy and in vivo distribution. The use of pipette mixing for in vitro screening versus microfluidic mixing for in vivo studies reflects a standard progression in LNP development. Pipette mixing is ideal for initial, small-volume formulation screening due to its simplicity and low material consumption, allowing for rapid parallel testing of various lipid compositions. However, for in vivo studies, which require robust reproducibility, narrow size distribution, and high batch-to-batch consistency to ensure reliable pharmacokinetics and biodistribution, microfluidic mixing is essential. This technique enables precise, controlled mixing that yields homogeneous, monodisperse LNPs suitable for systemic administration and scalability toward clinical translation (*Int. J. Pharm.* 2021, **601**, 120586; *Mol. Ther.* 2017, **25**, 1467-1475). In this work, LNPs for in vitro screening were formulated via pipette mixing, whereas the LNP samples for in vivo studies were prepared using microfluidic mixing. A similar approach was also used in the literature reports (*Nat. Biomed. Eng.* 2025, **9**, 2181-2195; *Nat. Mater.* 2025, **24**, 1852-1861; *Nat. Biomed. Eng.* 2024, **8**, 1412-1424).

Following the reviewer’s suggestion, we compared the physicochemical properties and mRNA transfection efficiency of LNPs prepared by two methods: pipette mixing (manual) and microfluidic mixing. The size, polydispersity index (PDI), and zeta potential of LNPs were quite similar regardless of manual or microfluidic mixing (**Supplementary Fig. 39a-c**). However, microfluidic mixing yielded consistently

higher encapsulation efficiencies for FLuc mRNA compared to manual mixing (**Supplementary Fig. 39d**). In DC2.4 cells, all microfluidically formulated LNPs exhibited significantly lower FLuc mRNA transfection activity than their manually mixed counterparts (**Supplementary Fig. 40a**). A similar phenomenon was also observed in HEK 293T cells, except for pDLS5 LNPs (**Supplementary Fig. 40b**). LNPs formulated via pipette or microfluidic mixing were not toxic to HEK 293T and DC2.4 cells (**Supplementary Fig. 40c, 40d**).

8) Furthermore, do you have cryo-EM images of the LNPs used for the in vivo studies? The internal structure appears unstructured in the LNPs formulated via pipette mixing, which may not be the case for LNPs prepared by microfluidic mixing. Including these images would help clarify structural differences.

Response: Shown in Figure 2j and k are cryo-TEM images of mRNA-loaded ALC-LNP and pDLS4-LNP, which were prepared by microfluidic mixing. We are sorry for not specifying this in the caption of the figure. The information has now been added to the caption of the figure in the revised manuscript. In addition, we have performed cryo-TEM analysis for pDLS2-LNP prepared via microfluidic mixing. Both pDLS2-LNP and pDLS4-LNP exhibited a lamellar structure on the particle surface and a relatively amorphous particle core (**Fig. 2k** and **Supplementary Fig. 45**). In contrast, ALC-LNP displayed an electron-dense core with no apparent lamellar structure, enclosed by a lipid bilayer-like envelope (**Fig. 2j**). Similar LNP structures (a lamellar shell with an amorphous core) were also reported in the literature for other types of LNPs (*Nat. Commun.* 2020, **11**, 983; *Nat. Chem.* 2024, **16**, 1687-1697).

9) Supplementary Figures 22-35 are missing data for the control samples. Since the focus of the paper is on PEG replacement, including the reference (PEG-containing) samples is essential to allow proper comparison and interpretation.

Response: In the functional screening and characterization of pDLS-LNPs, we used ALC-LNP as a control. All LNPs were prepared via pipette mixing. The particle size, zeta potential, mRNA encapsulation efficiency and cell viability for ALC-LNP have been added to the corresponding figures (**Supplementary Fig. 23-28**, **33-36**). Given the large number of pDLS lipids involved, the number of formulations that could be screened in each time was limited. Therefore, ALC-0159 at 1.6% was included as a positive control in every in vitro transfection experiment. Due to variability introduced by manual mixing, the transfected luminescence intensity of the manually prepared ALC-0159 formulation varied between batches. Therefore, the luminescence intensity of pDLS LNPs was normalized to that of ALC-LNP prepared at 1.6% (**Supplementary Fig. 29-32**). A similar normalization approach was also used in the literature (*Nat. Mater*. 2025, 24, 1852-1861). The original luminescence intensity results of pDLS-LNPs and their corresponding ALC-LNP are provided in **Supplementary Table 5** and **Table 6** for reference.

10) Supplementary Figures 26 and 27 do not specify which mRNA was encapsulated. This information is important, as Fluc mRNA is significantly larger in size and therefore more difficult to encapsulate. Additionally, please indicate whether these LNPs were prepared via pipette mixing or microfluidic mixing.

Response: In the functional screening study, FLuc mRNA was used and mRNA-loaded LNPs were prepared by pipette mixing. We have now added this information to the corresponding figures including **Supplementary Figures 26** and **27** (**Supplementary Figures 27** and **28** in the revised manuscript).

11) In the experimental section describing cellular uptake, it is stated:" On the next day, the cells were exposed to LNPs containing Cy5-labeled mRNA at a dose of 1000 ng." This is inconsistent with the cytotoxicity study, which was performed using a 100 ng dose. I recommend either repeating the in vitro study to assess uptake and protein expression at 100 ng, or performing a cytotoxicity assay at 1000 ng to ensure consistency and correlation.

Response: To evaluate the transfection efficiency and cytocompatibility of mRNA-loaded pDLS-LNPs *in vitro*, DC2.4 or HEK 293T cells were cultured in 96-well plates (1×10^4 per well) and incubated overnight. Cells were then treated with LNPs containing 100 ng of Fluc mRNA or SARS-CoV-2 mRNA per well. For cellular uptake study, DC2.4 cells were plated in 24-well plates (1×10^5 per well) and incubated overnight. The cells were then treated with LNPs containing Cy5-labeled mRNA at a dose of 1000 ng per well. Since the cell density per well in 24-well plates is 10-fold higher than that in 96-well plates, we used a proportionally increased mRNA dose.

Following the reviewer's suggestion, we assessed the cytotoxicity of ALC-LNP and pDLS2-LNP encapsulating Cy5-labeled mRNA at doses of 1000 ng and 2000 ng per well in 24-well plates. Both LNPs were prepared by pipette mixing. DC2.4 or HEK 293T cells were seeded in 24-well plates at a density of 1×10^5 cells per well and incubated overnight. The following day, cells were treated with LNPs containing Cy5-labeled mRNA at a dose of 1000 ng or 2000 ng per well. In both DC2.4 and HEK 293T cells, all LNPs maintained cell viability levels comparable to the untreated control ($p <$ 0.05, **Supplementary Fig. 41**). This result indicated that pDLS2-LNP and ALC-LNP were cytocompatible even at high doses.

12) Regarding the hemolysis assay, how does the lipid composition of the red blood cell (RBC) membrane compare to that of endosomal membranes? Adding one sentence comparison both would help to interpret the physiological relevance of the assay.

Response: Following the reviewer's suggestion, additional information about the physiological relevance of the hemolysis assay has been added to the revised manuscript. The relative capacity for LNP-mediated endosomal escape was simulated using an RBC hemolysis assay. RBC hemolysis was used as a surrogate assay for

endosomal escape due to similarities in their lipid bilayer (phospholipid and cholesterol) and glycocalyx compositions (*Biochem. J* 1985, **232**, 33-36). However, we note that this model is limited because the protein compositions of endosomes and RBCs do differ and endosomes do not have a membrane skeleton that is present in RBCs. This assay was performed at both physiological pH of 7.4 and endosomal pH of 5.5 (*News Physiol. Sci.* 2002, **17**, 1-5). Membrane lysis at pH 7.4 is an indication of toxicity, and lysis at 5.5 is a model for the ability of the LNPs to escape vesicular structures (e.g., endosomes/lysosomes) upon acidification. A similar approach was also used to study endosomal escape of LNPs in the literature reports (*Nat. Mater.* 2025, **24**, 1852-1861; *Nat. Mater*. 2021, 20, 701-710; *Nat. Commun.* 2020, **11**, 2424; *Proc. Natl. Acad. Sci. USA* 2013, **110**, 12881-12886).

13) How were the LNPs dosed for the ex vivo biodistribution experiment shown in Figure 5? Was the injected dose normalized to the body weight of the mice?

Response: For the ex vivo biodistribution experiment, the LNPs were dosed via subcutaneous injection at the dose of 2 μg per BALB/c mouse without normalizing it to the body weight of the mice. A similar experimental approach and administration route were also used in the literature reports on bioimaging studies (*Nat. Nanotechnol.* 2023, 18, 1105-1114; *Adv. Healthc. Mater.* 2023, 12, e2202528; *Cell*. 2020, 182, 1271-1283). Relevant experimental detail has been provided in the methods section of the revised manuscript.

14) I understand that the scope of the paper is vaccination related and that's the reason for the s.c. administration route. However, do you have any data on i.v. administration and if it changes biodistribution?

Response: Following the reviewer's suggestion, we have assessed the in vivo transfection efficiency and tissue distribution of pDLS-LNPs following intravenous (i.v.) administration. To evaluate the systemic mRNA delivery performance of pDLS-LNPs, we intravenously administered FLuc mRNA-loaded LNPs to mice and performed bioluminescence imaging at 6 h post-injection. Based on the whole-body imaging, both pDLS2-LNP and pDLS5-LNP exhibited bioluminescence patterns comparable to ALC-LNP (**Fig. 5a** and **Supplementary Fig. 47**). Quantification of relative bioluminescence radiance intensity (RBRI) demonstrated that pDLS5-LNP achieved systemic transfection efficiency equivalent to that of ALC-LNP (**Supplementary Fig. 48**). We further performed ex vivo bioluminescence imaging of harvested major organs at 6 h to assess mRNA transfection profiles and tissue distribution (**Fig. 5b-5d** and **Supplementary Fig. 49**). Similar to ALC-LNP, PEG-free pDLS-LNPs including pDLS2-LNP and pDLS5-LNP, primarily accumulated in the liver and spleen (**Fig. 5b-5d**). RBRI analysis indicated that pDLS5-LNP mediated hepatic expression at a level comparable to ALC-LNP, but significantly higher than that of pDLS2-LNP (**Fig. 5e**). In the spleen, both pDLS-LNPs showed RBRI levels similar to ALC-LNP (**Fig. 5e**). In the heart, kidneys, and lungs, the RBRI values of ALC-LNP

were comparable to those of pDLS5-LNP, but significantly exceeded the levels observed with pDLS2-LNP (**Fig. 5e**). These results demonstrated that pDLS lipids can effectively substitute PEGylated ALC-0159 in LNP formulations for efficient systemic mRNA delivery via i.v. administration.

Reviewer #2:

I co-reviewed this manuscript with one of the reviewers who provided the listed reports. This is part of the Nature Communications initiative to facilitate training in peer review and to provide appropriate recognition for Early Career Researchers who co-review manuscripts.

Response: We thank the reviewer for providing the constructive comments. Following the reviewer's suggestions, the manuscript has been revised thoroughly.

Response to the reviewer's comments:

Reviewer #2:

Dear Editor,

I have reviewed the revised version of this manuscript and the accompanying responses to my comments.

The authors have addressed very detailed all of my previous comments. The revisions have substantially improved the clarity, methodological transparency, and overall quality of the manuscript. I appreciate the responses provided in the rebuttal letter, and I am satisfied that the concerns raised during the initial review have been adequately resolved.

In its current form, I believe the manuscript is suitable for publication and recommend acceptance.

Response: We sincerely thank the reviewer for their careful evaluation of the revised manuscript and for the positive feedback. We are pleased that the revisions have addressed the concerns raised in the initial review and that the reviewer considers the manuscript suitable for publication.

Reviewer #4:

I co-reviewed this manuscript with one of the reviewers who provided the listed reports. This is part of the Nature Communications initiative to facilitate training in peer review and to provide appropriate recognition for Early Career Researchers who co-review manuscripts.

Response: We thank the co-reviewer for their time and contribution to the evaluation of our manuscript as part of the peer-review training initiative. We appreciate their careful assessment of our work.

Reviewer #5:

I co-reviewed this manuscript with one of the reviewers who provided the listed reports. This is part of the Nature Communications initiative to facilitate training in peer review and to provide appropriate recognition for Early Career Researchers who co-review manuscripts.

Furthermore, my personal comments are as follows;

Main comments:

1. The authors developed mRNA-LNP using biodegradable poly(D, L-serine) (pDLS) lipids as PEG-lipid alternatives. It should be mentioned that there are several mRNA-LNP, which have been developed using other hydrophilic lipid instead of PEGylated lipid (Polym Rev, 63(2) (2023) 394-436, DOI; 10.1080/15583724.2022.2106490)

Response: We thank the co-reviewer for their constructive comments and for highlighting relevant prior work. Following this suggestion, we have cited the recommended literature on the use of alternative hydrophilic lipids in mRNA-LNP formulations (Ref. 37, 38, 41, 42, 48) and have revised the manuscript accordingly.

2. Most researchers prepared mRNA-LNPs containing 1.0–2.5 mol% PEGylated lipid, which suppressed LNP aggregation and led to the control of a homogeneous particle size distribution between 50 and 180 nm. When the fraction of PEGylated lipid increases, the inflammatory secretion of cytokines is reduced. However, the expression of specific proteins from mRNA transcription was also found to decrease (Mol. Ther. Nucleic Acids. 2014, 3, e210. DOI: 10.1038/mtna.2014.61). Therefore, several PEG alternatives have been developed to avoid the disadvantage of using PEGylated nanoparticles, such as sugar-based molecules, polypeptides, polyoxazolines, and polyglycerols (ACS Appl. Nano Mater. 2020, 3, 10634-10645. DOI: 10.1021/acsanm.0c01834.; Angew. Chem. Int. Ed. Engl. 2010, 49, 6288-6308. DOI: 10.1002/anie.200902672; Polym Rev, 63(2) (2023) 394-436, DOI: 10.1080/15583724.2022.2106490). Polysarcosine is a polypeptoid based on an endogenous amino acid (N-methylated glycine) of sarcosine, which is reported to have potent stealth properties (Chem. Rev. 2016, 116, 1753–1802. DOI: 10.1021/acs.chemrev.5b00201; Macromol. Biosci. 2015, 15, 881-891. DOI: 10.1002/mabi.201500023; ACS Nano. 2018, 12, 8646-8661. DOI: 10.1021/acsnano.8b04433). Feneroli et al. reported that polysarcosine lipids do not show an acute immune response in mice and zebrafish embryos (ACS Nano. 2018, 12, 8646-8661. DOI: 10.1021/acsnano.8b04433.). Therefore, Nogueira et al. designed polysarcosine-conjugated lipids with different lengths (ACS Appl. Nano Mater. 2020, 3, 10634-10645. DOI: 10.1021/acsanm.0c01834). mRNA-LNPs prepared with the polysarcosine-conjugated lipid with different polymeric chain lengths and molar fractions enabled the control of the physicochemical characteristics of the mRNA-LNPs, such as their internal structure, morphology and particle size. mRNA-LNPs prepared with the polysarcosine-conjugated lipid with the optimal length showed decreased

cytokine inductions with higher specific protein production by mRNA compared with those prepared with DMG-PEG2000 (ACS Appl. Nano Mater. 2020, 3, 10634-10645. DOI: 10.1021/acsanm.0c01834). This study indicated that not only PEGylated lipids but also the development of other types of hydrophilic lipids, such as polysarcosine-conjugated lipids with an appropriate length (polymerization number (n)=23 in this study), are important for the efficient development of mRNA-LNPs (Polym Rev, 63(2) (2023) 394-436, DOI: 10.1080/15583724.2022.2106490).

Response: We thank the reviewer for the insightful and comprehensive comments and for highlighting prior work on PEG alternatives, including polysarcosine-conjugated lipids. We agree that several series of amphiphilic lipids have been explored to replace PEGylated lipids in mRNA-LNP formulations, with the aim of maintaining colloidal stability while reducing immunogenicity and preserving translational efficiency.

Polysarcosine-conjugated lipids represent an important class of PEG substitutes due to their stealth properties and low acute immunogenicity. In comparison, the polyserine (pDLS)-based lipids reported in this study provide a complementary structural approach with several potential advantages. First, polyserine contains pendant hydroxyl groups on every repeat unit, conferring higher intrinsic hydrophilicity than polysarcosine, which bears N-methylated amide side chains. This increased hydrophilicity may promote a dense hydration layer and effective steric stabilization, potentially enhancing stealth behavior and colloidal stability. Second, the hydroxyl functionality offers a versatile chemical handle for further functionalization, enabling conjugation of imaging agents or targeting ligands without introducing additional linker chemistries. Third, although sarcosine itself is a naturally occurring metabolite in mammalian systems, polysarcosine is a synthetic peptoid polymer whose N-substituted backbone is not produced biologically and is generally resistant to proteolytic degradation. In contrast, polyserine is a true polypeptide composed of a canonical amino acid and is therefore more likely to undergo enzymatic processing into metabolizable products.

Taken together, while polysarcosine lipids are effective PEG alternatives, polyserine (pDLS)-based lipids expand the design space by offering enhanced hydrophilicity, functional versatility, and potential biodegradability derived from a natural amino acid polymer. We have revised the manuscript to better acknowledge prior developments in alternative hydrophilic lipids and to clarify how the present work complements these approaches, with advantages, toward the rational design of PEG-lipid substitutes for mRNA-LNP vaccines and therapeutics.

3. As we can see most of researchers used a polysarcosine-conjugated lipid instead of PEGylated lipid, whereas the authors in this study used poly(D, L-serine) (pDLS)-conjugated lipid instead of PEGylated lipid. The authors should show or explain the merit of the usage of pDLS-conjugated lipids instead of polysarcosine-conjugated lipids.

Response: We thank the reviewer for this important question. We agree that polysarcosine (pSar)-conjugated lipids represent a well-studied class of PEG alternatives. Indeed, recent studies have shown that direct substitution of PEG lipids in current U.S. Food and Drug Administration (FDA)-approved LNP formulations with pSar-conjugated lipids can retain or even enhance mRNA delivery efficiency, while maintaining immunogenicity profiles comparable to conventional PEGylated LNPs (*Bioact. Mater.* 2024, **37**, 86-93). These findings validate the feasibility of replacing PEG with alternative hydrophilic polymers. However, the development of PEG-free LNPs that simultaneously achieve biosafety, delivery efficacy, and storage stability remains an unmet challenge for nucleic acid therapeutics.

In this context, poly(D,L-serine) (pDLS)-conjugated lipids offer a distinct and complementary design strategy with advantages. pDLS is a water-soluble, biodegradable, and biocompatible polypeptide with multiple pendant hydroxyl groups. Unlike peptoid-based polymers such as polysarcosine, pDLS forms a true polypeptide backbone composed of naturally occurring amino acid units, which may enable more predictable enzymatic degradability and metabolic compatibility. In addition, pDLS is nonionic and contains abundant hydroxyl functionalities that can serve as versatile chemical handles for further modification or conjugation. pDLS also allows control over chirality (D/L ratio) and degradation behavior, providing additional tunability for biomedical applications (*ACS Appl. Mater. Interfaces* 2021, **13**, 18454-18461).

In the present study, we demonstrate that a series of pDLS-conjugated lipids can directly replace PEG lipids in the clinically relevant LNP formulation. The resulting pDLS-LNPs exhibit favorable delivery performance, safety, and storage stability. Collectively, these results highlight pDLS lipids as a promising PEG-free alternative and expand the available design space beyond polysarcosine-based systems toward next-generation mRNA delivery nanocarriers.

4. The merit of polysarcosine-conjugated lipids is that polysarcosine is not a natural component, which would has slow biodegradable speed in vivo, whereas L-serine site on poly(D, L-serine) (pDLS)-conjugated lipid is expected to be degradated in vivo very

fast, probably around 10 min. That means the mRNA-LNP developed in this study pDLS site will be immediately digested (disappeared) in treatment in vivo. Although in vitro results showed good using (pDLS)-conjugated lipid, in vivo study should show similar results showing control without PEGlated lipid. One suggestion from the reviewer is that poly(D-serine)-conjugated lipid would be valuable to generate mRNA-LNP for the author's project goal, because poly(D-serine)-conjugated lipid will not be degradated immediately in vivo environment.

Response: We thank the reviewer for the constructive comments. Prior studies compared the critical aggregate concentration of PEG or polysarcosine (pSar) and found that these two polymers behave similarly, which suggests that pSar can serve similar functionality as PEG for antifouling applications (*Polymer* 2015, **67**, 240-248; *Langmuir* 2012, **28**, 16099-16107). Polysarcosine has been previously investigated as a PEG substitute for therapeutic protein conjugation (*Bioconjugate Chem.* 2018, **29**, 2232-2238). Other groups have investigated pSar as a surrogate to PEG by assessing its functionality to impart stealth-like properties in delivery systems such as liposomes (*Small*, 2019, **15**, e1904716; *J. Contr. Release.* 2020, **322**, 209-216), lipoplexes (*J. Contr. Release.* 2023, **356**, 1-13), and LNPs (*ACS Appl. Nano Mater.* 2020, **3**, 10634-10645; *Pharmaceutics* 2023,**15**, 2068). Many research studies have reported in recent years that the biocompatible, noncytotoxic, and stealth-like pSar does well in biomedicine. It should be noted, however, that pSar is a peptoid polymer that is largely resistant to enzymatic degradation, and the biodegradability of pSar has not been demonstrated up to date (*Polym. Chem.* 2011, **2**, 1900-1918; *Biomacromolecules* 2022, **23**, 1757-1764).

In contrast, poly(D,L-serine) (pDLS) is a biodegradable polypeptide whose degradation rate can be tuned by adjusting the D/L ratio (*ACS Appl. Mater. Interfaces* 2021, **13**, 18454-18461). Previous studies have shown that pDLS copolymers are not rapidly degraded; for example, pDLS with a D/L ratio of 5:5 exhibited less than 30% enzymatic degradation over 12 hours (*ACS Appl. Mater. Interfaces* 2021, **13**, 18454-18461). The presence of D-serine residues reduces susceptibility to proteolysis, enabling controlled rather than immediate degradation. Therefore, the pDLS used in this study is expected to provide sufficient stability in vivo while retaining the advantage of eventual biodegradability.

Following the reviewer's suggestion, we attempted to synthesize poly(D-serine)-conjugated lipid. However, poly(D-serine) lipid was found to be insoluble in water and

common organic solvents, which is consistent with the literature reports (*Biochemistry* 1963, **2**, 228-237; *J. Am. Chem. Soc.* 1968, **90**, 2760-2765; *ACS Appl. Mater. Interfaces* 2021, **13**, 18454-18461). This poor solubility makes poly(D-serine) unsuitable for LNP formulation.

Taken together, pDLS provides a balanced profile between stability and biodegradability: it is sufficiently stable to maintain LNP integrity during delivery while avoiding the long-term persistence associated with PEG-like polymers. Our in vivo data further demonstrate that pDLS-LNPs achieve effective mRNA delivery, supporting their functional suitability as PEG-free alternatives.

Another comments:

1. mRNA-LNP using pDLS usage showed negative zeta potential compared to mRNA-LNP using PEGylated lipid (Fig. 2i). When mRNA-LNP using polysarcosine-lipid, zeta potential will not show negative value because of chemical structure of polysarcosine.

Response: We thank the reviewer for this comment. Slightly negative or near-neutral zeta potentials are commonly observed for mRNA-LNPs, including clinically relevant formulations, and values within approximately ±6 mV are generally considered typical (*Nat. Biomed. Eng.* 2024, **8**, 544-560; *Angew. Chem. Int. Ed.* 2024, **63**, e202318515). The surface charge of mRNA-LNPs is determined by the overall formulation rather than by the hydrophilic polymer alone. Key factors include the chemical structure and protonation behavior of the ionizable lipid, the N/P ratio, and the environmental pH (*Int. J. Pharm.* 2025, **682**, 125941; *Commun. Biol.* 2021, **4**, 1-15; *Int. J. Pharm.* 2025, **680**, 125796; *J. Control. Release* 2025, **377**, 162-173). Additionally, the preparation process (especially post-processing steps) can also alter their surface charge characteristics.

Consistent with this, LNPs formulated with polysarcosine-conjugated lipids do not universally exhibit neutral or positive zeta potentials. For example, ALC-0315-based LNPs containing pSar lipids were reported to be slightly negatively charged, except for TETAMINE-pSar25 (*Bioact. Mater.* 2024, **37**, 86-93). However, the zeta potentials of SM-102-based LNPs were all slightly positive. These findings indicate that the ionizable lipid framework and formulation parameters dominate the resulting surface charge.

Therefore, the slightly negative zeta potential observed for pDLS-LNPs in our study is within the expected range for mRNA-LNP systems and is not inconsistent with

the use of alternative amphiphilic lipids.

2. In Fig 2 caption; "LNPs were prepared via pipette mixing" should be revised as "mRNA-LNPs were prepared via pipette mixing".

Response: We thank the reviewer for pointing this out. The amendments have been made accordingly in the revised manuscript.

3. In Fig. 5, 6 and 8, the results of mRNA-pDLS-LNPs are almost the same to mRNA-PEGylated-LNPs after i.v. injection in vivo. Then why pDLS2-LNP or other pDLS-LNPs encapsulated with SARS-CoV-2 mRNA vaccines could elicit significantly higher robust or durable cellular and humoral immune responses in mice than ALC-LNP in Fig. 7?

Response: We thank the reviewer for this important question. While IVIS bioimaging after a single injection shows that pDLS-LNPs and PEGylated ALC-LNPs exhibit broadly similar whole-body biodistribution profiles, comparable organ-level luciferase signals do not necessarily translate into identical immunological outcomes. Vaccine efficacy depends not only on bulk distribution but also on factors such as cellular uptake within immune-relevant compartments, persistence of antigen expression, and the effects of repeated dosing.

First, the IVIS studies (**Figs. 5**, **6**, and **8**) evaluated biodistribution following a single injection, whereas the vaccination experiments involved two doses. Repeated administration can amplify differences between formulations that are not apparent after a single exposure. In particular, anti-PEG antibodies are known to be induced after PEG-LNP vaccination and can reduce delivery efficiency upon subsequent dosing (Ref. 22). Consistent with this, anti-PEG antibodies were detected in mice receiving ALC-LNP, whereas no anti-pDLS2 antibodies were observed in pDLS2-LNP-immunized animals (Supplementary Fig. 64). This likely contributes to the enhanced cellular and humoral immune responses observed with pDLS-LNPs.

Second, immunological readouts demonstrate that pDLS-LNP vaccines induce strong adaptive responses. SARS-CoV-2 spike mRNA-loaded pDLS-LNPs elicited robust anti-spike IgG titers (**Fig. 7b** and **Supplementary Fig. 52, 53**) and neutralizing activity (**Fig. 7c**) comparable to ALC-LNP after two doses (**Fig. 7b**, **c**), with persistence over 11 weeks. Notably, pDLS2-LNP induced significantly higher IFN-γ secretion (**Fig. 7d**, **e**), indicating enhanced cellular immunity. In addition, IgG1 ELISpot assays and

flow cytometry analysis showed that pDLS2- and pDLS3-LNPs generated significantly greater numbers of IgG1-secreting (**Fig. 7f** and **Supplementary Fig. 55**) and RBD-specific (**Fig. 7g**) B cells than ALC-LNP, suggesting improved induction of memory B-cell response. Furthermore, most RBD-specific B cells exhibited a memory phenotype, co-expressing the memory markers PD-L2 and CD80 (**Fig. 7h**), suggesting their potential for rapid differentiation into antibody-secreting cells upon antigen reexposure..

Taken together, these results indicate that although overall biodistribution after a single injection is similar, pDLS-LNPs exhibit advantages during repeated vaccination, likely due to the absence of anti-PEG immunity and formulation-dependent effects on immune activation. Additional discussion has been added to the revised manuscript.

4. In the description of the ex vivo bioluminescence images (panels j–o), it is unclear whether there is an additional panel following "o." If so, the corresponding sample name is missing. If not, the labeling should be corrected. This needs clarification.

Response: Figs. 4j-o showed representative ex vivo bioluminescence images of major organs of mice treated with FLuc mRNA-loaded LNPs at 24 h after s.c. administration: **j**, PEGylated ALC-LNP; **k**, pDLS1-LNP; **l**, pDLS2-LNP; **m**, pDLS3-LNP; **n**, pDLS4-LNP; **o**, pDLS5-LNP. Panels **4j–n** exhibited representative ex vivo bioluminescence images of the major organs from one mouse, while those from the remaining four mice in these groups can be found in **Supplementary Fig. 48**. Notably, **Fig. 4o** showed ex vivo bioluminescence images of major organs of five mice (n = 5). To make them easier to distinguish, we have used clearly colored borders to differentiate each group in the revised manuscript.

5. In Fig. 5, only three formulations (PEGylated ALC-LNP, pDLS2-LNP, and pDLS5-LNP) were evaluated following intravenous administration. The rationale for selecting these specific candidates is not provided. The selection criteria must be clearly stated. Furthermore, the inclusion of tail-vein administration appears disconnected from the primary focus on subcutaneous delivery. The authors should explicitly justify the purpose of the i.v. study and clarify how it strengthens the overall conclusions. As presented, its placement disrupts the logical flow of the manuscript.

Response: We thank the reviewer for this important comment. The intravenous (i.v.) study in **Fig. 5** was included to provide a complementary assessment of systemic transfection performance and was conducted in response to a request from another

reviewer.

The specific formulations (PEGylated ALC-LNP, pDLS2-LNP, and pDLS5-LNP) were selected as representative candidates spanning the range of polymerization degrees (DPs) evaluated in this work. pDLS2 represents low-DP pDLS lipids, whereas pDLS5 represents high-DP pDLS lipids, which exhibited robust performance in the subcutaneous (s.c.) studies. These two formulations therefore capture the structural extremes of the pDLS series while keeping the number of animal experiments minimal. PEGylated ALC-LNP was included as a clinically relevant control.

Although the primary focus of this study is s.c. delivery for vaccination, evaluation after i.v. administration provides important information on whether pDLS-LNPs retain functional mRNA delivery capability under systemic conditions, where biodistribution differs markedly. Demonstrating comparable transfection profiles to ALC-LNP following i.v. injection supports the broader applicability of pDLS lipids and confirms that replacing PEG does not compromise systemic delivery performance.

We agree that this rationale was not sufficiently explained in the original manuscript. The text has been revised to clarify the selection criteria for the tested formulations, the purpose of the i.v. study, and its relationship to the primary s.c. investigations, thereby improving the logical flow.

6. For safety assessment, the authors selected a pDLS-LNP formulated with a lipid of DP 23, described as being close to pDLS2. The rationale for this choice is insufficiently justified. If DP 23 is merely structurally similar to pDLS2, the added value of introducing this additional formulation is unclear. The authors must provide a clear, data-driven justification for selecting this candidate for safety evaluation.

Response: We thank the reviewer for this important comment. The safety assessment was performed using the same pDLS2 formulation employed in the majority of the functional studies. The storage studies were performed using a second synthetic batch of the pDLS2 lipid because the initial batch used for earlier experiments had been fully consumed during the course of the study. The lipid in this batch (DP ≈ 23) was selected to match as closely as possible the degree of polymerization of the original pDLS2 lipid, thereby maintaining continuity with the main dataset.

Precise control of polymerization degree during synthesis is inherently challenging; therefore, we verified batch equivalence experimentally. ^{1}H NMR analysis confirmed that pDLS2 lipid from the two batches exhibited closely matched DPs, with variations

of ≤ 3 units (**Supplementary Fig. 22**). LNPs prepared from these batches showed similar physicochemical properties, mRNA encapsulation efficiencies (**Supplementary Fig. 37**), and in vitro transfection performance in both HEK293T and DC2.4 cells (**Supplementary Fig. 38**), demonstrating that the small DP difference does not materially affect functional outcomes.

Accordingly, the DP ≈ 23 lipid represents a reproducible batch of the same pDLS2 formulation used throughout the study. Using this batch enabled storage evaluation at the scale required while preserving comparability with prior results. Using this representative formulation, pDLS-LNPs exhibited excellent stability after 6 months at -80 °C, maintaining size distribution, near-neutral zeta potential, encapsulation efficiency, and in vivo transfection performance (**Fig. 8l-8p** and **Supplementary Fig. 67**).

7. The fluorescence distribution pattern shown in Fig. 8i appears markedly different from that presented in Fig. 4. The anatomical localization and signal distribution do not seem consistent between the two figures. Please clarify whether the animals in Fig. 8i and Fig. 4 were treated using the same injection route (e.g., s.c. vs i.v.), injection site, dose, and imaging time point. If the administration conditions differed, this must be clearly stated. If the conditions were intended to be identical, the discrepancy in biodistribution requires explanation.

Response: We thank the reviewer for pointing this out. The injection sites in the mice shown in **Fig. 4** and **Fig. 8i** were completely different, which resulted in distinct luminescence signal distribution patterns. Following the reviewer's suggestion, relevant experimental detail has been provided in the methods of the revised manuscript. In **Fig. 4**, LNPs encapsulating 1.0 μg of FLuc mRNA (FLuc mRNA-LNPs were prepared via microfluidic mixing) **were s.c. injected into the left hind legs** of female BALB/c mice (n = 5 per group) for evaluating transfection efficiency and biodistribution in vivo. At 6 h or 24 h post-injection, mice received intraperitoneal (i.p.) injections with 200 μL of 15 mg/mL VivoGlo luciferin (Promega, USA). After 10 min, luminescence signals from mice were detected using IVIS Spectrum imaging system (PerkinElmer, USA).

Fig. 8i showed the bioluminescence images of mice that were subcutaneously injected with cryopreserved or fresh FLuc mRNA-loaded pDLS-LNP for comparing the *in vivo* transfection efficiency. Female BALB/c mice **were subcutaneously**

administered at the tail base with cryopreserved or fresh FLuc mRNA-loaded pDLS-LNP (FLuc mRNA-LNPs were prepared *via* microfluidic mixing) at a dose of 1.0 μg mRNA per mouse (n = 3 per group). After 6 h, mice were intraperitoneally injected with 200 μL of 15 mg/mL VivoGlo luciferin (Promega, USA). After 10 min, luminescence signal from mice were detected using IVIS Spectrum imaging system (PerkinElmer, USA). The total flux values of whole mouse body were measured using Living Image software (PerkinElmer, USA).

Reviewer #6:

I co-reviewed this manuscript with one of the reviewers who provided the listed reports. This is part of the Nature Communications initiative to facilitate training in peer review and to provide appropriate recognition for Early Career Researchers who co-review manuscripts.

Response: We thank the co-reviewer for their time and contribution to the evaluation of our manuscript as part of the peer-review training initiative. We appreciate their careful assessment of our work.

Reviewer #7:

Summary:

This study reports the development of polypeptide-based lipid nanoparticles (pDLS-LNPs) as alternatives to PEGylated LNPs for mRNA delivery. The authors introduce biodegradable, nonionic poly(D, L-serine) lipids to replace PEG, aiming to reduce anti-PEG antibody formation and improve redosing potential. Through systematic structural screening, optimized pDLS formulations achieved stable nanoparticles with high mRNA encapsulation and strong transfection efficiency. Compared with the clinically used ALC-LNP formulation, pDLS-LNPs demonstrated enhanced in vitro delivery and robust immune responses in vivo when delivering SARS-CoV-2 spike mRNA, without detectable systemic toxicity. Notably, repeated dosing induced minimal anti-pDLS IgM production, and the lead formulation maintained stability under frozen storage for over six months. Overall, the work positions polypeptide-engineered LNPs as a potential alternative platform for safer and effective mRNA delivery.

Though the study is robust with both in vitro and in vivo experimentation, there are certain concerns that need to be addressed by the authors before it is publishable in Nature Communications.

Response: We thank the reviewer for the constructive comments. Following the reviewer's suggestions, the manuscript has been revised accordingly.

Major Concern:

1. In the current version of the manuscript, the timeline for repeated dose testing is twice, which is minimal, and generally acceptable is at least 3, if not 4. While I understand that rerunning experiments at this point is resource constrained, I suggest providing a rationale and supporting it with additional research on studies that have been completed with a similar repeat dosage regime.

Response: We thank the reviewer for this thoughtful comment regarding the repeat-dosing timeline. We agree that a three- or four-dose regimen can provide a more comprehensive assessment of repeated administration effects. However, a two-dose schedule was chosen to reflect clinically approved and widely implemented vaccination regimens for mRNA-LNP-based vaccines.

Pivotal clinical trials for FDA-approved mRNA vaccines, such as mRNA-1273 and BNT162b2, successfully utilized a two-dose primary series to establish foundational safety and immunogenicity (*N. Engl. J. Med.* 2021, **385**, 239-250; *N. Engl. J. Med.* 2021, **384**, 403-416). Recent pre-clinical studies specifically designed to investigate the impact of repeat administration on LNP performance have also used a two-dose regimen. For example, a two-dose regimen of mRNA-LNP vaccines has been shown to substantially boost anti-PEG antibody levels in humans, which can subsequently affect the efficacy of repeat administration (*ACS Nano* 2022, **16**, 11769-11780). Following the reviewer's suggestion, we have clarified the rationale for the two-dose schedule in the Methods section and cited relevant supporting studies.

2. Clarification on the Pipette mixing versus the microfluidic mixing

The manuscript currently uses pipette mixing and microfluidic mixing approaches interchangeably across multiple sections, which significantly dilutes the impact and clarity of the work. It is harder for the reader to connect the result to those obtained with pipette mixing versus those obtained with microfluidic mixing. The absence of a clear rationale for switching between preparation methods makes it difficult for the reader to understand why a single approach was not consistently used. While I understand the possible reason for the authors is due to facile sample preparation and quick initial testing, these have to be mentioned in the manuscript to give the same

context for the readers as well. If not in the main text, consider including that in the SI text.

Response: We thank the reviewer for this insightful and important observation. We agree that the rationale for using both pipette mixing and microfluidic mixing should be clearly articulated to avoid confusion. Following the reviewer's suggestion, we have added a detailed explanation in the **Supplementary experimental methods 1.5**, and clarified the preparation method used in the Methods section and the figure legends.

The use of pipette mixing and microfluidic mixing approaches for mRNA-LNP formulations is a common strategy to balance the needs of different experimental phases. Pipette mixing was used for the initial, functional screening of pDLS-LNP formulation parameters (e.g., pDLS lipid structures, mole ratios of pDLS lipids) as well as in vitro studies. This method allowed for rapid preparation with minimal material consumption, which was critical for efficiently identifying lead pDLS-LNP formulations. Once lead pDLS-LNP formulations were identified, microfluidic mixing was used for all subsequent *in vivo* studies. This method was chosen for its superior control over particle size, low polydispersity, and high batch-to-batch reproducibility, which are essential for translational relevance. This staged approach (rapid manual screening followed by controlled microfluidic preparation for in vivo validation) is also adopted in the literature reports (*Nat. Biomed. Eng.* 2025, **9**, 2181-2195; *Nat. Mater.* 2025, **24**, 1852-1861; *Nat. Biomed. Eng.* 2024, **8**, 1412-1424).

3. Formulation Rationale:

Is there a specific rationale for selecting ALC-0315 and not SM-102 as the comparator lipid? Given that SM-102 is widely used in clinically validated mRNA-LNP systems, the choice of ALC should be justified explicitly. So, in the future follow-up works can also bank on such an explanation.

Response: We thank the reviewer for this important question. In this study, ALC-0315 was selected as the ionizable lipid because our primary objective was to evaluate pDLS lipids as substitutes for the PEG-lipid component while keeping the rest of the formulation constant. Using a single, clinically validated ionizable lipid allowed us to attribute observed differences directly to the pDLS substitution without confounding effects from changes in the ionizable lipid chemistry.

ALC-0315, the ionizable lipid used in the BNT162b2 vaccine, represents one of the two major clinically approved mRNA-LNP formulations and is clinically validated

(*N. Engl. J. Med.* 2020, **383**, 2603-2615). This made it an appropriate benchmark for evaluating whether pDLS-conjugated lipids can replace PEG-lipids. We agree that SM-102, the ionizable lipid used in the mRNA-1273 vaccine (*N. Engl. J. Med.* 2021, **384**, 403-416), is equally important and clinically relevant. However, including multiple ionizable lipids would have introduced additional variables beyond the scope of the present study.

We agree that evaluating pDLS lipids across different ionizable lipids would be valuable to determine the generality of this approach. Such head-to-head comparisons between ALC-0315- and SM-102-based LNP formulations are an important direction for future work and could further support the translational potential of pDLS-LNPs.

4. Figure-Specific Comments

Figure 2i:

Both sets of error bars should be clearly included in the figure. The variability appears substantial, particularly in the zeta potential measurements. Notably, pDLS1 and pDLS5 exhibit considerable fluctuation in zeta potential. The authors should address the implications of this variability, explain why it was observed in the tested samples, and clarify how variability in surface charge may affect stability, biodistribution, or biological interpretation.

Response: We thank the reviewer for pointing this out. Following the reviewer's suggestion, both sets of error bars have been included in the **Fig. 2i** in the revised manuscript. Indeed, pDLS1 and pDLS5 exhibited considerable fluctuation in zeta potential. We note that this fluctuation was observed in a single measurement set for these specific formulations. Across all replicates and independent experiments, the average values of zeta potential for all pDLS-LNPs remained consistently **within ± 5.5 mV**, which is well within the typical range observed for mRNA-LNP systems (*Nat. Protoc.* 2025, **20**, 2618-2651; *J. Pharm. Sci.* 2022, **111**, 690-698; *Nat. Biomed. Eng.* 2024, **8**, 544-560; *Angew. Chem. Int. Ed.* 2024, **63**, e202318515). We attribute the isolated fluctuation to instrumental variability, such as minor electrode drift or transient electrical noise during that particular measurement session, rather than an inherent instability of the formulations. Importantly, this fluctuation was not reproduced in subsequent independent experiments, where pDLS5 exhibited stable and consistent zeta potential values (within ± 5.5 mV) comparable to other formulations (**Supplementary Fig. 39c**).

Figure 2k:

The manuscript states "Comparable structural characteristics…". However, one formulation (ALC) is described as more amorphous, while the pDLS particles appear at least twice as large as ALC-LNPs. Despite the use of microfluidic mixing, particle sizes remain substantially larger than in the control. The statement should therefore be moderated to reflect these structural differences. In addition, the authors should provide a rationale for the larger particle sizes observed in the pDLS formulations. It would also be important to clarify whether the in vitro testing showed variable transfection due to the larger size. Furthermore, the lack of size convergence despite microfluidic mixing requires explanation, and possible rationales should be included.

Response: We thank the reviewer for pointing this out. We agree that the original statement should be moderated to reflect the observed structural differences, and the manuscript has been revised accordingly.

Cryo-TEM imaging revealed distinct internal morphologies for the two formulations. ALC-LNP displayed an electron-dense core without apparent lamellar ordering, enclosed by a lipid bilayer-like envelope (**Fig. 2j**). In contrast, both pDLS2-LNP and pDLS4-LNP exhibited a lamellar structure on the surface and a relatively amorphous core (**Fig. 2k** and **Supplementary Fig. 46**). Consistent with these structural differences, pDLS-LNPs showed larger particle sizes (~87-100 nm) than ALC-LNP (~70 nm). This is likely attributable to the fundamental structural differences between PEG and poly(D, L-serine) (pDLS). Unlike the highly flexible, random-coil conformation of PEG (*Polymer* 2008, **49**, 1132-1136; *J. Phys. Chem. B* 2008, **112**, 719-723; *Soft Matter* 2011, **7**, 6515-6521), polypeptides typically exhibit **more rigid secondary structures** due to potential intramolecular hydrogen bonding along their backbone (*ACS Cent. Sci.* 2019, **5**, 229-236; *Polymer* 2022, **263**, 125499; *Biomacromolecules* 2023, **24**, 3580-3588). During self-assembly, the relatively more rigid polypeptide chains may occupy a larger excluded volume at the LNP surface compared to the compact, coiled PEG chains. Previous studies revealed that the rigidity of polymer chains has been shown to critically influence their packing behavior and the resulting nanoparticle structure (*Phys. Rev. E* 2016, **94**, 032504; *J. Polym. Sci., Part A: Polym. Chem.* 2011, **49**, 118-126).

The absence of size convergence despite microfluidic mixing likely reflects intrinsic formulation properties rather than processing limitations. Microfluidic mixing

controls nucleation and growth conditions but cannot fully compensate for differences in polymer architecture, interfacial packing, and steric stabilization mechanisms. A similar phenomenon was also observed by other groups. For example, although microfluidic mixing was used, the particle size of PEG-free LNPs stabilized with PEG alternatives (e.g., poly(carboxybetaine), polysarcosine) is generally larger than that of PEGylated LNPs (*Nat. Mater.* 2025, **24**, 1852-1861; *Bioact. Mater.* 2024, **37**, 86-93).

Regarding functional consequences, in vitro studies showed that pDLS-LNPs with relatively larger particle sizes (~87-100 nm) mediated significantly higher mRNA transfection efficiency (**Fig. 2m**). This enhancement likely arises from a combination of particle size, lipid composition, and internal structure rather than size alone. Importantly, pDLS-LNPs also achieved robust mRNA delivery in vivo.

Additional discussion has been added to the revised manuscript.

Figure 2c–2h (DLS Analysis):

The manuscript states "Narrow size distributions (PDI < 0.14)." While a PDI < 0.2 is generally considered acceptable, a value of 0.14 does not necessarily indicate a narrow monodisperse system and may still represent a mixed population. The authors should moderate this claim, provide additional supporting data, such as number-weighted versus intensity-weighted distributions, and clarify how "narrow" is defined in this context.

Response: We thank the reviewer for pointing this out. We agree that a PDI < 0.14, while indicative of relatively homogeneous nanoparticle populations, does not necessarily imply a strictly monodisperse system. Accordingly, the term "narrow" has been moderated in the revised manuscript to reflect the commonly accepted interpretation for LNP formulations.

Following the reviewer's suggestion, we have provided number-weighted size distributions in the revised manuscript. As shown in **Supplementary Fig. 45a-f**, the number-weighted size distributions closely match the intensity-weighted size distributions (**Fig. 2c–2h**) for all formulations and exhibit single peaks, with no evidence of additional peaks or larger aggregates.

Figure 3 – Erythrocyte Model Concerns:

The use of erythrocytes to demonstrate delivery and cytotoxicity raises several questions. First, regarding biological context, it is unclear whether the erythrocytes

were characterized for maturity or subtype and whether any assay was performed to confirm uniformity.

a. The rationale for choosing erythrocytes as the target cell type should also be clarified.

Response: In this study, the membrane fusion activity of ALC-LNP and pDLS2-LNP was evaluated using a hemolysis assay, where hemoglobin release from red blood cells (RBCs) serves as a proxy for membrane disruption (**Fig. 3d**). This surrogate assay was used due to the comparable lipid bilayer (phospholipid and cholesterol) and glycocalyx compositions shared between RBCs and endosomal membranes (*Biochem. J.* 1985, **232**, 33-36; *Proc. Natl Acad. Sci. USA* 2013, **110**, 12881-12886). It should be noted, however, that the model has inherent limitation, as endosomal and RBC membranes differ in protein content and RBCs contain a membrane skeleton absent in endosomes. This assay was conducted at both physiological pH (7.4) and endosomal pH (5.5). Hemolysis observed at pH 7.4 reflects potential toxicity, whereas lysis at pH 5.5 serves as an indicator of LNP-mediated membrane disruption under acidic conditions, modeling escape potential from acidic vesicles such as endosomes and lysosomes (*News Physiol. Sci.* 2002, **17**, 1-5). A similar approach was also used to study endosomolytic activity of RNA-LNPs in the literature reports (*Nat. Mater.* 2025, **24**, 1852-1861; *Nat. Mater.* 2021, **20**, 701-710; *Nat. Commun.* 2020, **11**, 2424; *Proc. Natl. Acad. Sci. USA* 2013, **110**, 12881-12886).

b. Second, mature erythrocytes lack nuclei, so it is unclear how Hoechst staining was observed and whether this is consistent with the cell type used. This requires clarification in the Materials and Methods section, particularly if there is a specific explanation.

Response: We thank the reviewer for this comment and would like to clarify that **Hoechst staining was not performed on erythrocytes**. The endosomal escape studies shown in **Fig. 3e-k** were conducted using **DC2.4 cells**, an immortalized murine dendritic cell line, which possess nuclei and are therefore compatible with Hoechst nuclear staining. DC2.4 cells were also employed for the in vitro mRNA transfection studies described in this work. To avoid confusion, we have revised the legend of **Fig. 3d-g** to explicitly state the cell type used.

c. Third, fluorescence localization alone does not confirm endosomal escape. To substantiate this claim, 3D confocal reconstruction and colocalization with endosomal

markers would be required. The current imaging appears insufficient to support spatial escape claims.

Response: We thank the reviewer for this important comment. We agree that fluorescence localization alone cannot definitively prove endosomal escape. In this study, conventional 2D confocal laser scanning microscopy was used to evaluate intracellular distribution of Cy5-labeled mRNA delivered by LNPs. As shown in **Fig. 3e-g**, DC2.4 cells treated with Cy5-labeled mRNA-loaded LNPs exhibited diffuse and bright red fluorescence (Cy5-mRNA) throughout the cytoplasm, alongside punctate green fluorescence from endosomes labeled with LysoTracker Green. The intensity-based pixel matching colocalization analysis revealed that, in DC2.4 cells treated with pDLS-LNP for 4 h, the red fluorescence signal was found outside the green-labeled endosomal compartments, suggesting successful release of Cy5-mRNA into the cytoplasm. The plot profile based on a single line scan is performed for intensity-based pixel matching colocalization analyses. The pixel values in the image are directly used in the evaluation of spatial correlation. To further evaluate colocalization between Cy5-labeled mRNA and endosomal compartments, we have performed pixel-wise colocalization analysis across multiple independent images, and Pearson's correlation coefficient was calculated using ImageJ/Fiji (**Supplementary Fig. 47**). It was found that pDLS2-LNP exhibited a lower Pearson's R value (no threshold) of 0.55 ± 0.05 compared to 0.64 ± 0.06 for ALC-LNP, indicating a reduced degree of colocalization. These analyses support enhanced endosomal escape by pDLS-LNPs relative to ALC-LNPs. Colocalization analysis using statistical methods such as Pearson's or Mander's coefficients has also been used to quantify the degree of colocalization in the literature reports (*Sci. Signal.* 2017, **10**, eaal1683; *Am. J. Physiol. Cell Physiol.* 2011, **300**, C723-42; *Methods Cell Biol.* 2014, **123**, 395-408; *J. Microsc.* 2006, **224**, 213-232; *Cytometry, Part A* 2010, **77**, 733-742; *J. Microsc.* 2006, 224, 213-232).

We acknowledge that 3D reconstruction and additional endosomal markers could provide further spatial resolution; however, such experiments were beyond the scope of the present study. To avoid overinterpretation, the manuscript has been revised to clarify that the imaging data indicate cytosolic distribution consistent with endosomal escape rather than providing definitive proof.

d. Finally, the plot profile analysis based on a single line scan is not representative of overall intensity, can introduce user bias, and does not provide quantitative robustness.

Intensity quantification across multiple independent images should be performed, replicate analysis should be included, statistical treatment should be provided, and overinterpretation based on line-scan profiles should be avoided.

Response: We thank the reviewer for this important comment. We agree that a single line scan is not suitable for quantitative analysis, and can introduce bias. The line-scan profile was used solely for intensity-based pixel matching colocalization within a single representative image, rather than for quantifying overall fluorescence intensity. In this study, we performed the flow cytometry analysis to quantify Cy5 fluorescence intensity across large cell populations, The results, presented in **Fig. 3b** and **3c**, represent mean fluorescence intensity from three independent experiments with statistical analysis, thereby providing population-level evidence of mRNA-LNP uptake.

To further evaluate colocalization between cy5-labeled mRNA and endosomal compartments, we performed pixel-wise colocalization analysis across multiple independent confocal images, and Pearson's correlation coefficient was calculated using ImageJ/Fiji (**Supplementary Fig. 47**). It was found that pDLS2-LNP exhibited a lower Pearson's R value (no threshold) of 0.55 ± 0.05 compared to 0.64 ± 0.06 for ALC-LNP, indicating a reduced degree of colocalization. These analyses support enhanced endosomal escape by pDLS-LNPs relative to ALC-LNPs. Colocalization analysis using statistical methods such as Pearson's or Mander's coefficients has also been used to quantify the degree of colocalization in the literature reports (*Sci. Signal.* 2017, **10**, eaal1683; *Am. J. Physiol. Cell Physiol.* 2011, **300**, C723-42; *Methods Cell Biol.* 2014, **123**, 395-408; *J. Microsc.* 2006, **224**, 213-232).

Minor comments:

5. Citation concerns on PEG Immunogenicity Discussion (Page 3)

a. The statement, "The high prevalence of PEG-specific antibodies in the general population has raised significant safety concerns…", is currently supported by a single citation that is not directly relevant to mRNA vaccines. While PEG immunogenicity is an important topic, the manuscript appears to overstate the prevalence issue, base a strong claim on limited and non-specific evidence, and rely on review articles rather than original research. In particular, References 25 and 26 are review articles rather than primary research, and the cited article supporting the claim of "high prevalence" does not directly relate to mRNA-LNP vaccines. Although there is a broader trend in RNA therapeutics toward exploring PEG alternatives, the clinical implications have not

yet been conclusively established, and instances of immunogenic outcomes are generally associated with repeated use.

Response: We thank the reviewer for this important observation. The manuscript has been revised to provide a more balanced and evidence-based description supported by primary research studies.

Pre-existing anti-PEG antibodies have been detected in 98-99% of unvaccinated individuals, with approximately 3-4% of the population identified as "supercarriers" exhibiting markedly elevated levels (*Vaccine* 2023, **41**, 4561-4570). These antibodies likely arise from widespread exposure to PEG-containing products including foods, cosmetics, and pharmaceuticals (*J. Pharm. Sci.* 2024, **113**, 555-578). While the clinical implications of pre-existing anti-PEG antibodies are still under investigation, studies have shown that individuals with high antibody levels may be at increased risk for hypersensitivity reactions, including rare allergic response to PEGylated mRNA-LNP vaccination (*Vaccine* 2023, **41**, 4561-4570; *J. Allergy Clin. Immunol.* 2021, **147**, 2075.e2-2082.e2).

We have moderated the text to avoid overstating the prevalence or clinical consequences while acknowledging that PEG immunogenicity has motivated ongoing research into alternative stealth materials for mRNA delivery systems (*Nat. Mater.* 2025, **24**, 1665; *Sci. Adv.* 2025,**11**, eady6460).

6. It is premature to conclude that the storage conditions have been fully optimized without a systematic evaluation of excipients and comprehensive time-resolved stability studies. Therefore, the authors should moderate their claims regarding long-term stability at -80 °C and avoid implying that the formulation is fully optimized based on the current data.

Response: We thank the reviewer for pointing this out. An amendment has been made accordingly in the revised manuscript. Our results suggested that pDLS-LNP showed promising cryopreserved stability even after 6 months of storage at -80 °C; good size distribution, near-neutral zeta potential, comparable encapsulation efficiency, and effective *in vivo* transfection compared with fresh pDLS-LNP (**Fig. 8l-8p** and **Supplementary Fig. 67**).

Reviewer #8:

I co-reviewed this manuscript with one of the reviewers who provided the listed reports.

This is part of the Nature Communications initiative to facilitate training in peer review and to provide appropriate recognition for Early Career Researchers who co-review manuscripts.

Response: We thank the co-reviewer for their time and contribution to the evaluation of our manuscript as part of the peer-review training initiative. We appreciate their careful assessment of our work.

Response to the reviewers' comments:

Reviewer #5:

The authors revised the manuscript following reviewer's comments. Now the manuscript is acceptable for the publication in Nature Communications.

Response: We sincerely thank the reviewer for their careful evaluation of the revised manuscript and for the positive feedback. We are pleased that the revisions have addressed the concerns raised in the initial review and that the reviewer considers the manuscript suitable for publication.

Reviewer #6:

I co-reviewed this manuscript with one of the reviewers who provided the listed reports. This is part of the Nature Communications initiative to facilitate training in peer review and to provide appropriate recognition for Early Career Researchers who co-review manuscripts.

Response: We thank the co-reviewer for their time and contribution to the evaluation of our manuscript as part of the peer-review training initiative. We appreciate their careful assessment of our work.

Reviewer #7:

The authors have addressed all our comments, and the manuscript can be published in its current, latest submitted form.

Response: We sincerely thank the reviewer for their careful evaluation of the revised manuscript and for the positive feedback. We are pleased that the revisions have addressed the concerns raised in the initial review and that the reviewer considers the manuscript suitable for publication.

Reviewer #8:

I co-reviewed this manuscript with one of the reviewers who provided the listed reports. This is part of the Nature Communications initiative to facilitate training in peer review and to provide appropriate recognition for Early Career Researchers who co-review manuscripts.

Response: We thank the co-reviewer for their time and contribution to the evaluation of our manuscript as part of the peer-review training initiative. We appreciate their careful assessment of our work.
